# Trackable Tolerogenic Macrophages Integrate PD‐L1 and Rapamycin Signaling to Suppress Alloimmune Responses in Transplantation

**DOI:** 10.1002/advs.202520420

**Published:** 2026-02-08

**Authors:** Yihui Wang, Yuan Song, Yuji Xie, Junmin Zhang, Jing Liu, Wenyuan Wang, Ying Xu, Wenqian Wu, Wuqi Zhou, Qiaofeng Jin, Jing Wang, Yali Yang, Li Zhang, Tang Gao, Mingxing Xie

**Affiliations:** ^1^ Department of Ultrasound Medicine Union Hospital Tongji Medical College Huazhong University of Science and Technology Wuhan China; ^2^ Clinical Research Center for Medical Imaging in Hubei Province Wuhan China; ^3^ Hubei Province Key Laboratory of Molecular Imaging Wuhan China; ^4^ Key Laboratory of Biological Targeted Therapy Huazhong University of Science and Technology, Ministry of Education Wuhan China

**Keywords:** allograft rejection, alloreactive T cell, macrophage, programmed death ligand‐1

## Abstract

Cell‐based immunotherapies represent a promising strategy for transplant tolerance, yet challenges remain in graft targeting, in vivo tracking, and comprehensive regulation of alloreactive T cells. Here, we developed trackable tolerogenic macrophages (**TTMs**), engineered to overexpress PD‐L1, incorporate azide groups for bioorthogonal labeling, and encapsulate rapamycin nanoparticles (RAPA NPs). Following intravenous administration in murine allografts, **TTMs** preferentially homed to inflamed grafts and were visualized in vivo. **TTMs** reduced graft inflammation and prolonged allograft survival up to 35 days, significantly longer than PD‐L1 or RAPA monotherapy groups. Mechanistic studies showed that PD‐L1 ligation of PD‐1 suppressed Th1 differentiation and CD8^+^ T‐cell activation, reducing IFN‐γ/TNF‐α and thereby attenuating complement activation and PI3K/Akt/mTOR signaling. This enhanced rapamycin‐mediated mTOR inhibition and promoted Foxp3^+^ Treg induction with increased IL‐10 production. The elevated IL‐10 further strengthened PD‐1/PD‐L1 signaling, forming a feedback loop that maintained a tolerogenic microenvironment. Together, these findings highlight **TTMs** as multifunctional therapeutic cells that integrate graft homing, real‐time tracking, and dual‐pathway immunoregulation to advance precision immunotherapy in transplantation.

## Graphical abstract

We developed a macrophage‐based therapeutic platform, termed trackable tolerogenic macrophages (**TTM**), for dual‐function immunomodulation and visualization in vivo. **TTM** cells were engineered to overexpress PD‐L1, incorporate bioorthogonal cell surface tags, and load rapamycin for sustained release. This platform demonstrated excellent biocompatibility, minimal toxicity, and selectively targeted graft rejection sites, suppressing Th1/CD8+ T‐cell activation and promoting Treg differentiation via PD‐L1/RAPA signaling. Our findings highlight the translational potential of **TTM** as a precise, trackable immune‐modulating therapy, offering a novel strategy for inducing durable transplant tolerance in clinical settings.

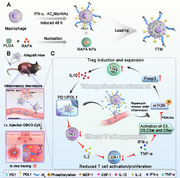

## 1 Introduction

Organ transplantation is the most effective treatment for patients with end‐stage organ failure. Nevertheless, transplant rejection remains a formidable barrier, significantly restricting graft survival and compromising patient quality of life [1, 2]. Widely used immunosuppressants, such as tacrolimus (FK506) and Cyclosporine A (CsA), effectively control acute rejection but provide limited protection against chronic immune activation and may lead to adverse effects, including infection, malignancy, and nephrotoxicity [3, 4]. In addition, these drugs disrupt the function and stability of regulatory T cells (Tregs), which are essential for maintaining immune tolerance [3]. There is an urgent need for strategies that prevent rejection without compromising Treg function.

Alloreactive T cells are central mediators of graft rejection, making them key targets for immunoregulation [4, 5]. The programmed death‐1 (PD‐1) receptor and its ligand PD‐L1 constitute a central inhibitory checkpoint that enforces peripheral tolerance [6]. Through PD‐1 ligation, PD‐L1 suppresses effector T‐cell responses and promotes the differentiation and stability of regulatory T cells [7, 8, 9]. In contrast, disruption of PD‐1/PD‐L1 signaling via genetic deletion or checkpoint blockade enhances T cell proliferation, derives Th1 polarization, and accelerates graft loss [10, 11]. Therapeutically enhancing PD‐1/PD‐L1 interactions, such as through systemic delivery of PD‐L1‐Ig fusion protein, has been shown to significantly prolong cardiac graft survival in both CD28^–/–^ and wild‐type mice treated with immunosuppressants [12, 13]. However, soluble PD‐L1‐Ig displays limited receptor clustering and immunosuppressive efficacy relative to its membrane‐bound form, resulting in systemic immune suppression and related toxicity [14].

To address these limitations, cell‐based approaches have been developed to enhance PD‐L1–mediated immunoregulation. Human embryonic stem cells [15, 16], hematopoietic stem cells [17], mesenchymal stem cells [18, 19], dendritic cells [20], and stem cells derived from extracellular vesicles [21, 22, 23, 24] have been engineered to express high levels of membrane‐bound PD‐L1. These platforms offer advantages such as sustained PD‐L1 presentation, the capacity to suppress effector T‐cell activation, and the potential to promote local immune tolerance in transplant settings. Although preclinical outcomes are promising, challenges remain in achieving precise graft targeting, multilevel regulation of alloreactive T‐cell activity, and real‐time traceability, which limit mechanistic understanding and clinical translation [12, 13].

Rapamycin (RAPA), a potent mTOR inhibitor, enhances PD‐L1‐mediated checkpoint immunoregulation by simultaneously restricting effector T cell proliferation and promoting Foxp3^+^ Treg differentiation [25, 26]. PD‐L1 attenuates TCR signaling at the membrane, and RAPA disrupts intracellular metabolic and cell‐cycle programs required for T‐cell activation [27, 28]. Integration of these complementary pathways within the graft microenvironment may achieve coordinated inhibition of effector T‐cell responses while enhancing regulatory T‐cell differentiation, thereby fostering immune tolerance [29, 30]. Yet, delivering both signals in a localized and coordinated manner remains a considerable challenge. Macrophages, owing to their intrinsic homing to inflamed tissues and prolonged persistence in vivo [31], represent promising candidates for this role, but their potential as programmable and traceable therapeutic cells has not been fully realized.

**FIGURE 1:**
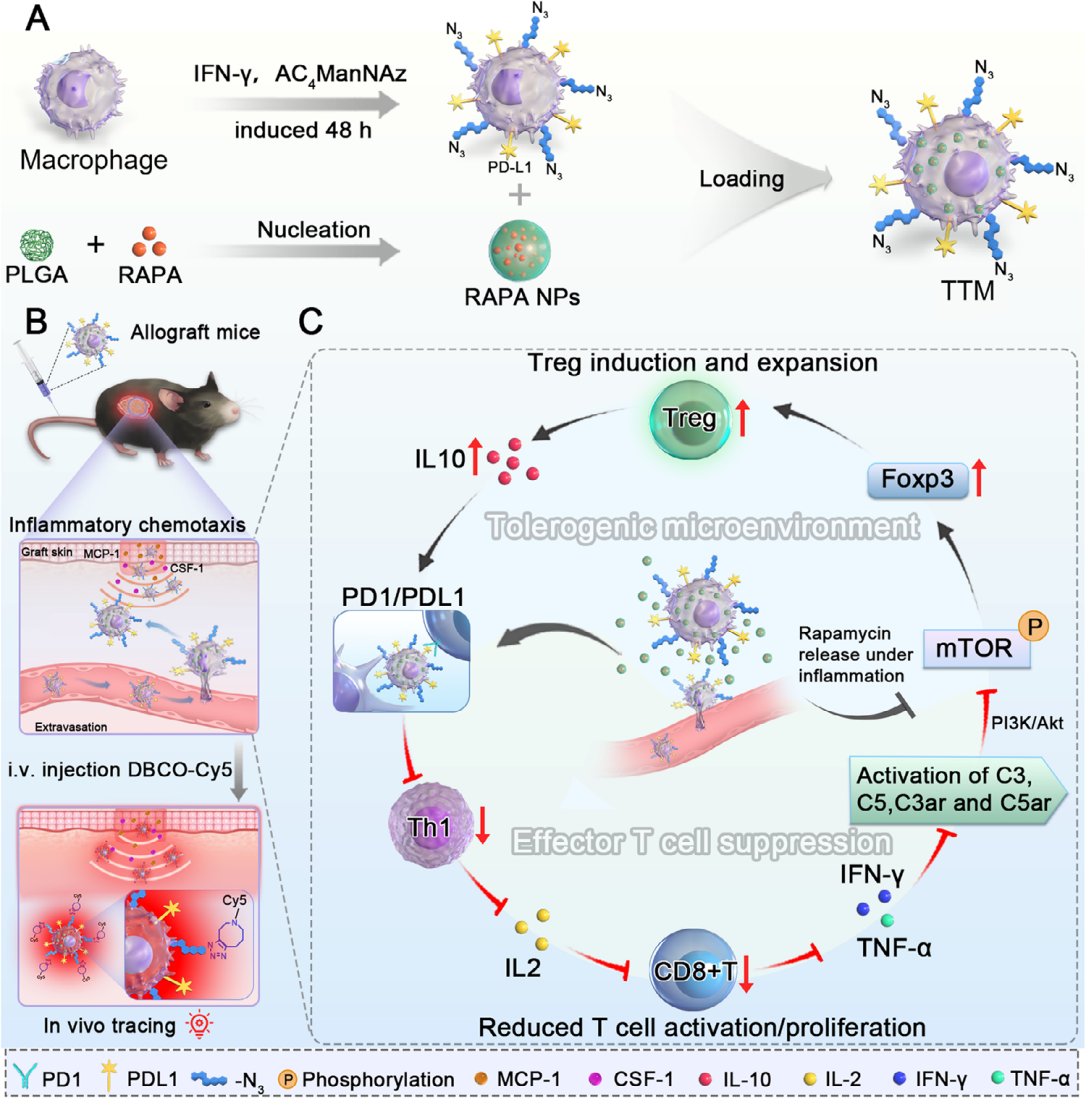
Design, graft targeting, and immunoregulatory mechanism of trackable tolerogenic macrophages (**TTMs**). (A) **TTMs** were generated by IFN‐γ stimulation and azide‐based metabolic labeling to upregulate PD‐L1 expression and enable bioorthogonal tagging, followed by phagocytic loading of rapamycin nanoparticles (RAPA NPs); (B) After intravenous injection, **TTMs** migrated to inflamed allografts in response to chemotactic cues and were visualized in vivo using DBCO‐Cy5 labeling; (C) Within grafts, PD‐L1/PD‐1 signaling suppressed Th1 differentiation and CD8^+^ T‐cell activation, reducing IFN‐γ/TNF‐α and subsequent complement activity. This alleviated PI3K/Akt/mTOR signaling, enhanced rapamycin‐mediated inhibition, and promoted Foxp3^+^ Treg induction. Treg‐derived IL‐10 further reinforced PD‐1/PD‐L1 activity, suggesting a feedback loop that stabilized a tolerogenic microenvironment.

In this study, we developed trackable tolerogenic macrophages (**TTMs**), a macrophage‐based system designed for dual‐pathway immunoregulation and in vivo imaging. **TTMs** were engineered in two steps: in the first step, macrophages were simultaneously stimulated with IFN‐γ to induce PD‐L1 expression and metabolically labeled with azido sugars to introduce bioorthogonal groups; in the second step, the cells phagocytosed rapamycin‐loaded PLGA nanoparticles (RAPA NPs) (Figure 1A). After intravenous administration in a murine skin allograft model, **TTMs** selectively homed to graft sites, as verified by in vivo click labeling with DBCO‐Cy5 (Figure 1B). Within grafts, PD‐L1/PD‐1 signaling suppressed Th1 differentiation and CD8^+^ T‐cell activation, leading to decreased IFN‐γ and TNF‐α production and consequent attenuation of complement activation (C3, C5, C3aR, C5aR). This reduced complement activity further downregulated PI3K/Akt/mTOR signaling, thereby enhancing the inhibitory effect of rapamycin on mTOR. This cooperative cascade promoted Foxp3 expression and Treg induction, while Treg‐derived IL‐10 strengthened PD‐1/PD‐L1 signaling, forming a positive feedback loop that maintained a tolerogenic graft microenvironment (Figure 1C). Collectively, these findings support a macrophage‐based therapeutic platform that integrates immune regulation with real‐time tracking for targeted immunomodulation in transplantation.

## 2 Results

### 2.1 Preparation and Characterization of Trackable Tolerogenic Macrophages (TTMs)

**FIGURE 2:**
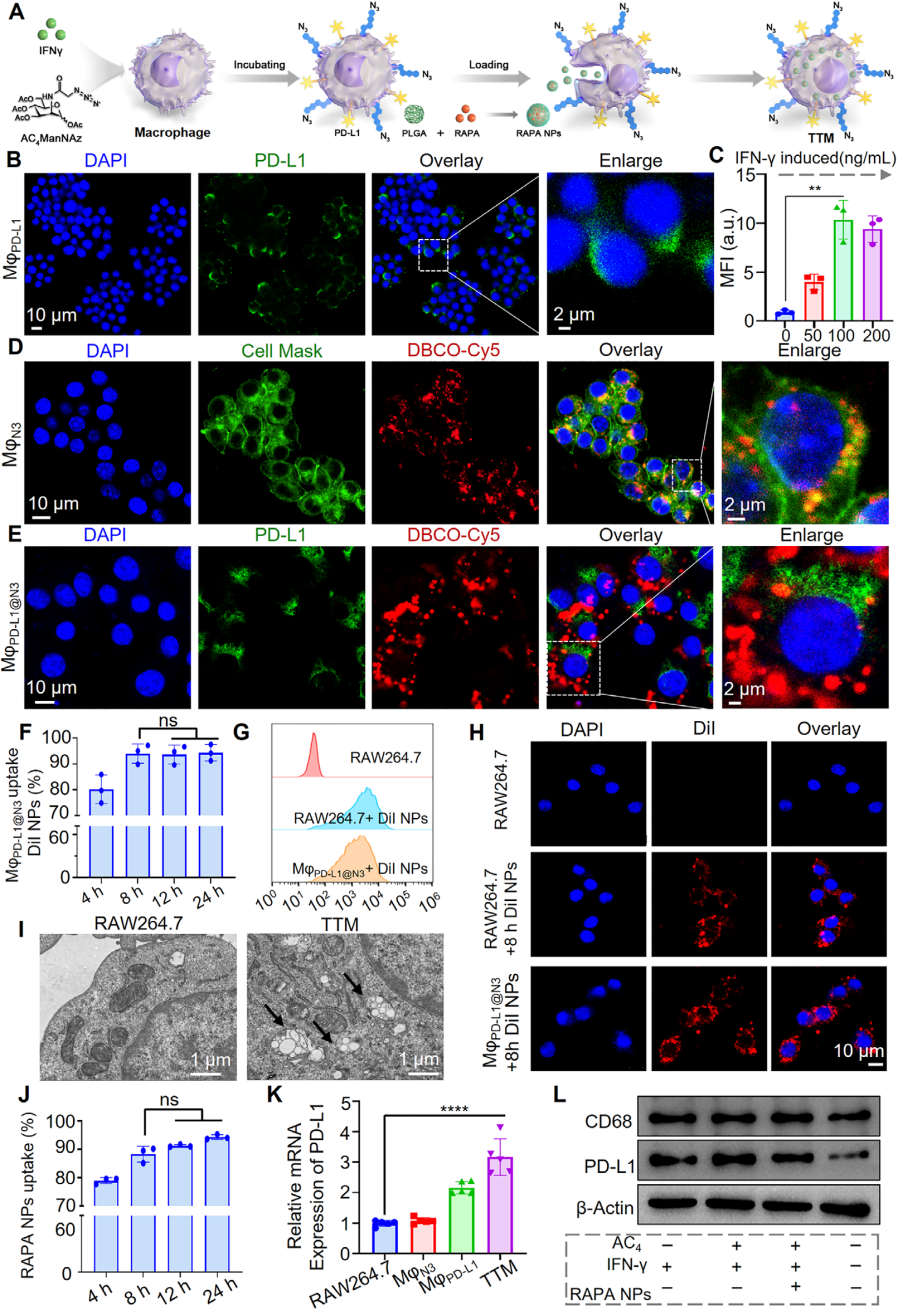
Preparation and characterization of trackable tolerogenic macrophage (**TTM**). (A) Schematic of the dual‐modification and nanoparticle‐loading strategy used to generate **TTMs**; (B–C) IFN‐γ stimulation induced PD‐L1 expression in RAW264.7 macrophages, shown by immunofluorescence and quantified by mean fluorescence intensity (MFI); (D) Azide groups (─N_3_) were metabolically incorporated on the cell surface, verified by colocalization of DBCO‐Cy5 (red) with membrane dye (green); (E) Confocal imaging Mφ_PD‐L1@N3_ cells showed spatial separation between PD‐L1 (green) and ─N_3_ (red) on the cell membrane. Scale bar: 10 µm; Enlarged: 2 µm; (F–H) Flow cytometry and confocal microscopy demonstrated preserved uptake of DiI‐labeled nanoparticles in Mφ_PD‐L1@N3_ compared to RAW264.7 cells (*n* = 3). Scale bar: 10 µm; (I) Transmission electron microscopy (TEM) confirmed intracellular localization of RAPA NPs (arrows). Scale bar: 1 µm; (J) HPLC analysis indicated stable nanoparticle uptake over 24 h (*n* = 3, ns = not significant); (K) qPCR revealed increased PD‐L1 expression in **TTMs** compared with controls (*n* = 5, ****p < 0.0001); (L) Western blot analysis showed that engineering modifications did not alter CD68 expression while increasing PD‐L1. AC_4_:AC_4_ManNAz. Data are presented as mean ± SEM. Repeated‐measures one‐way ANOVA with Dunnett's post‐hoc test for (C), (F), (J), and (K).

To construct **TTMs**, macrophages were simultaneously stimulated with IFN‐γ to upregulate PD‐L1 and metabolically labeled with N‐azidoacetylmannosamine‐tetraacylated (AC_4_ManNAz) to introduce azide groups, followed by phagocytic loading of rapamycin‐encapsulated PLGA nanoparticles (Figure 2A). Optimization of surface engineering conditions for **TTM** construction was first performed. IFN‐γ is a well‐established inducer of PD‐L1 through JAK‐STAT signaling [32, 33, 34], and immunofluorescence confirmed a dose‐dependent increase, with 100 ng/mL yielding an ∼11.5‐fold elevation of membrane PD‐L1 (Figure 2B,C; Figure S1). For metabolic labeling, azide groups were introduced by incorporating AC_4_ManNAz into membrane sialic acids, enabling subsequent bioorthogonal conjugation with DBCO–Cy5 [35, 36]. Labeling efficiencies increased with AC_4_ManNAz concentration but reached a plateau above 50 µm, which was therefore selected as the optimal dose (Figure S2). Confocal imaging showed colocalization of Cy5 fluorescence with membrane markers, confirming selective surface incorporation (Figure 2D; Figure S3). Flow cytometry further demonstrated stable and uniform labeling, with 93.7% of cells remaining positive after 48 h (Figures S4–S6). Under these optimized conditions, macrophages were simultaneously stimulated with IFN‐γ and labeled with AC_4_ManNAz to generate dual‐modified cells (Mφ_PD‐L1@N3_). Dual‐modified macrophages (Mφ_PD‐L1@N3_) exhibited distinct, non‐overlapping membrane localization of PD‐L1 and azide groups (Figure 2E; Figure S7). This indicated that the modifications did not sterically interfere, thereby likely preserving PD‐L1's functionality for checkpoint engagement.

Given that phagocytic capacity is essential for nanoparticle loading, we next evaluated whether dual surface modifications affected this intrinsic function. Flow cytometry showed that Mφ_PD‐L1@N3_ internalized >90% of DiI‐labeled PLGA nanoparticles within 8 h, which was selected as the incubation period for nanoparticle loading in **TTM** preparation (Figure 2F). Moreover, Mφ_PD‐L1@N3_ exhibited uptake comparable to macrophages, as confirmed by flow cytometry and confocal microscopy (Figure 2G,H).

We next incorporated rapamycin nanoparticles to generate **TTMs**. Rapamycin‐loaded PLGA nanoparticles (RAPA NPs) were ∼230 nm in diameter, carried a negative zeta potential (–9.29 mV), and remained stable for at least 30 days (Figure S8). HPLC showed a gradual release profile, with cumulative release reaching 10.2% at 24 h and 15.0% at 72 h (Figure S9). Transmission electron microscopy confirmed intracellular uptake of RAPA NPs by **TTMs**, with loading efficiencies of ∼94% at 8 h and ∼95% at 24 h (Figure 2I,J). Western blot and quantitative Polymerase Chain Reaction (qPCR) demonstrated that nanoparticle loading did not alter CD68 expression, while PD‐L1 remained upregulated by about 3.2‐fold compared with untreated macrophages (Figure 2K,L). Time‐course analysis further showed that PD‐L1 expression was sustained for 48 h but declined at 96 h (Figure S10A,B). As shown in Figure S10C, PD‐L1 mRNA levels remained stable and robust for at least 48 h post‐induction (showing no statistically significant decline compared to the peak induction level at 0 h). However, a significant decrease (∼25%) was observed by 96 h. This quantitative data confirms that the therapeutic window of our engineered **TTMs** extends to at least 48 h, providing a solid biological rationale for the 48‐h dosing interval employed in our in vivo efficacy studies.

### 2.2 Chemotactic Function, Inflammation‐Responsive Drug Release, and Biosafety of TTMs

**FIGURE 3:**
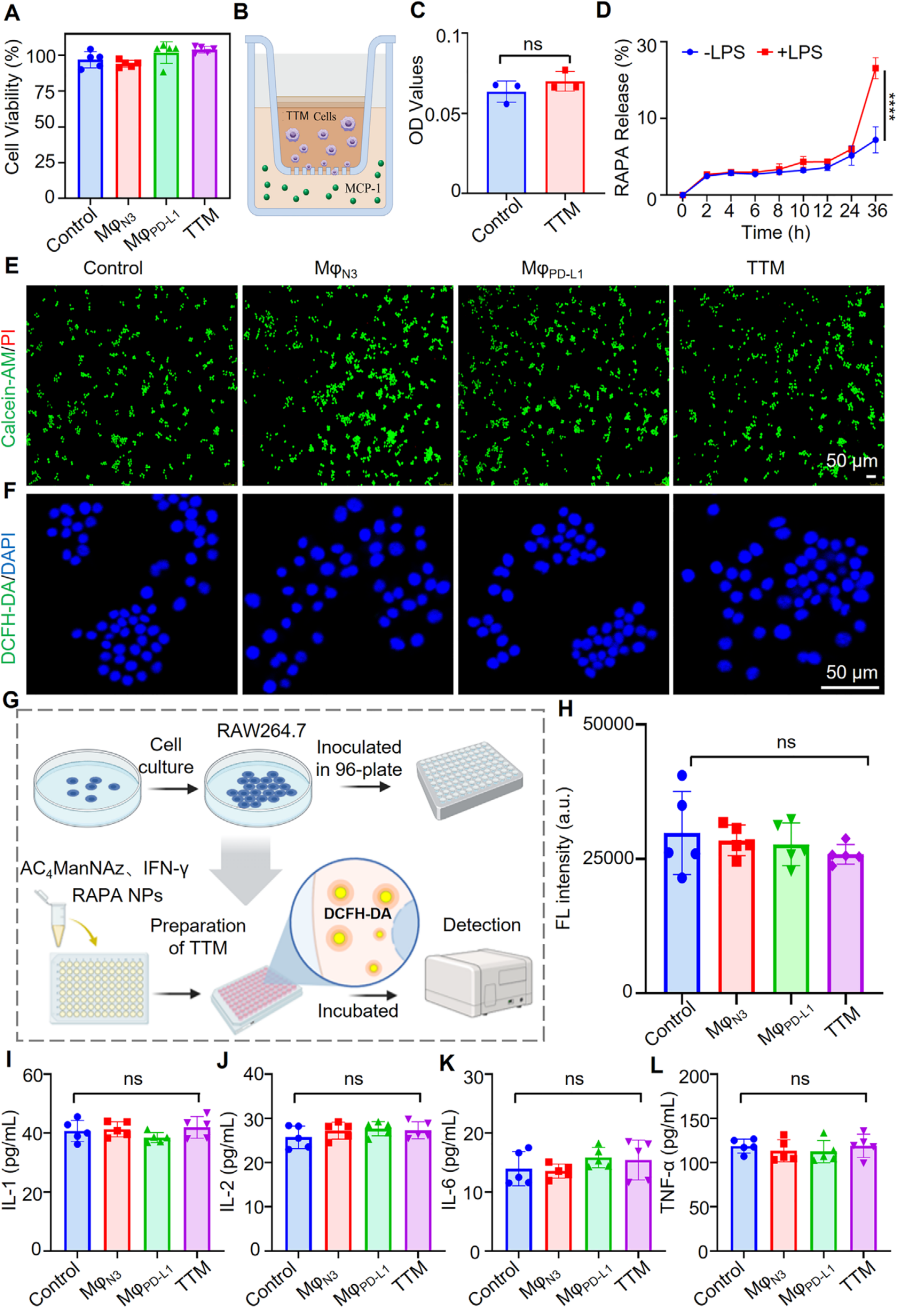
In vitro evaluation of **TTM** bioactivity and biosafety (A) Cell viability of TTMs measured by CCK‐8 assay; (B‐C) Transwell migration toward MCP‐1 quantified by crystal violet staining (*n *= 3, ns = not significant); (D) Inflammation‐responsive rapamycin release from **TTM**s under LPS stimulation. Significance indicated as ****p < 0.0001; (E) Live/dead staining (Calcein‐AM/PI) showing cell viability; (F–H) Intracellular ROS analysis using DCFH‐DA probe: confocal imaging (F), schematic workflow (G), and fluorescence intensity quantification (H). Figure 3G was created with BioRender.com; (I–L) ELISA quantification of IL‐1, IL‐2, IL‐6, and TNF‐α secretion in control and engineered macrophage groups. *n *= 5. ns, not significant. Each scatter point represents an independent biological replicate. Data are presented as mean ± SEM. Two‐tailed unpaired *t‐*test with Welch's correction for (C) and (D). Repeated‐measures one‐way ANOVA with Dunnett's post‐hoc test for (H–L).

Macrophages infiltrate grafts during rejection in response to chemotactic gradients [37, 38]. To assess whether engineering altered this function, cell viability and migration were examined. CCK‐8 assays showed >95% viability after IFN‐γ and AC_4_ManNAz treatment, comparable to unmodified controls (Figure 3A). Transwell and scratch assays demonstrated similar MCP‐1‐induced migration between **TTMs** and RAW264.7 cells (Figure 3B,C; Figure S11), confirming that surface modification did not impair chemotaxis.

Next, we investigated whether **TTMs** release rapamycin in response to inflammatory activation. HPLC analysis revealed that at 36 h, **TTMs** released 10.8 ± 2.5% of encapsulated rapamycin under basal conditions. In contrast, LPS stimulation significantly enhanced this release to 24.8 ± 2.0% at the same time point, representing a 2.3‐fold increase (Figure 3D). Live‐cell confocal imaging of DiI‐labeled nanoparticles revealed reduced intracellular and increased extracellular signals upon LPS treatment, consistent with exocytosis (Figure S12). Time‐lapse imaging further confirmed dynamic release (Movies S1 and S2).

Cytotoxicity and oxidative stress were assessed in engineered macrophages. Live/dead staining showed predominantly viable cells with minimal PI‐positive signals (Figure 3E). ROS levels, measured by DCFH‐DA fluorescence, were unchanged after modification, consistent with untreated controls (Figure 3F–H), indicating that surface labeling and nanoparticle loading did not induce oxidative stress.

Macrophage polarization was then evaluated. Under basal conditions, CD86 expression in **TTMs** was comparable to RAW264.7 cells. LPS stimulation induced a ∼1.2‐fold increase in CD86 expression in RAW264.7 cells, whereas no significant change was observed in **TTMs** (Figure S13), consistent with a lower M1 polarization potential [39, 40, 41]. Immunofluorescence staining further showed that LPS increased CD86 but decreased CD206 in RAW264.7 cells, whereas **TTMs** displayed lower CD86 and higher CD206 expression (Figure S14). These findings indicate that the modifications do not promote pro‐inflammatory polarization and may favor an M2‐like phenotype. Finally, cytokine secretion was examined. ELISA revealed no significant differences in IL‐1, IL‐2, IL‐6, or TNF‐α levels among **TTM**, Mφ_N3_, Mφ_PD‐L1_, and control groups (Figure 3I–L), further supporting the immunological safety of **TTMs** in vitro.

**FIGURE 4:**
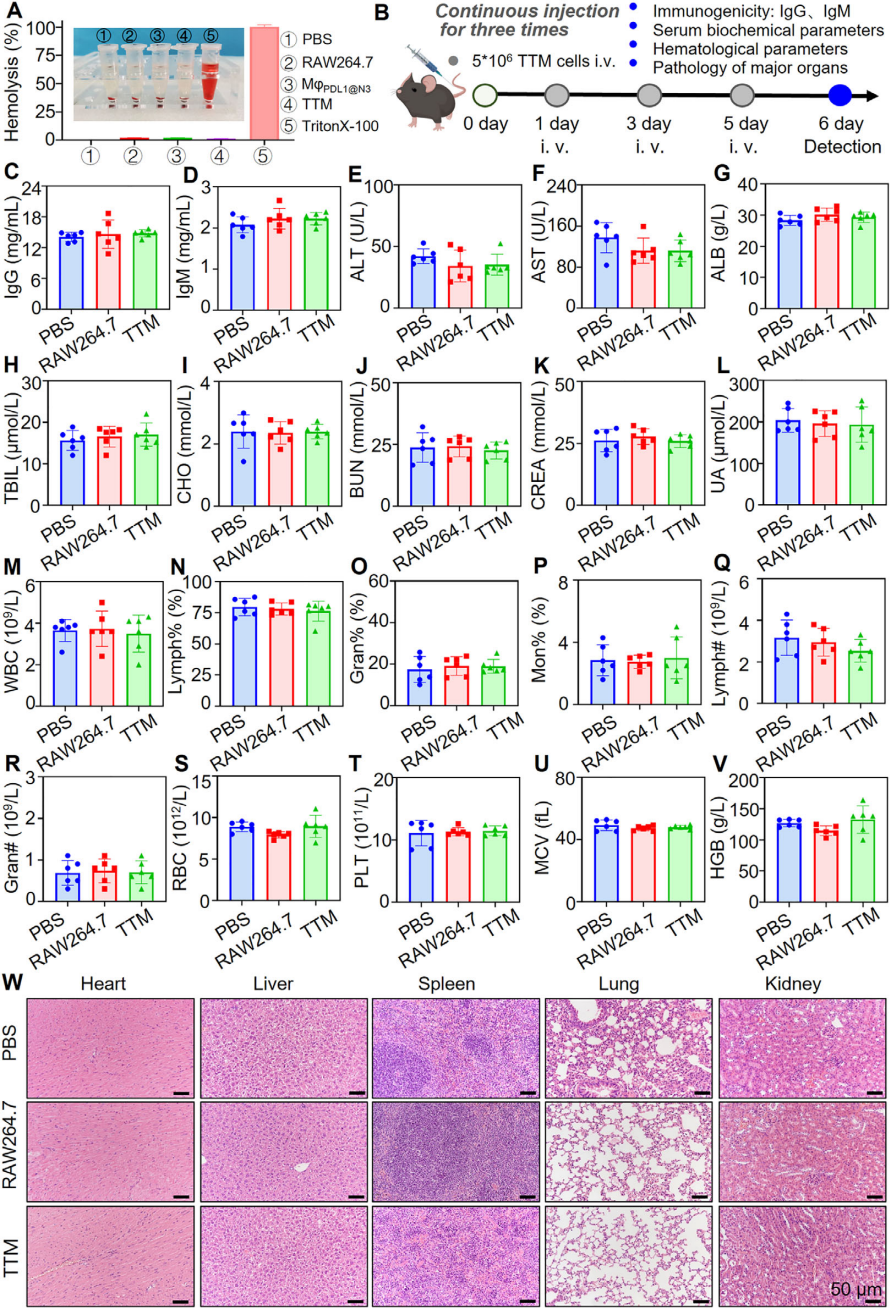
In vivo biosafety evaluation of **TTM** after repeated intravenous administration. (A) Hemolysis of red blood cells after incubation with PBS, RAW264.7, Mφ_PD‐L1@N3_ **_,_ TTMs** or Triton X‐100 (positive control); (B) Experimental timeline: three intravenous injections at 48 h intervals followed by biosafety analyses; (C,D) Serum IgG and IgM levels (ELISA); (E–I) Liver function parameters: Alanine aminotransferase (ALT), aspartate aminotransferase (AST), albumin (ALB), total bilirubin (TBIL), and total cholesterol (CHO); (J–L) Kidney function parameters: Blood urea nitrogen (BUN), creatinine (CREA), and uric acid (UA); (M–V) Hematological parameters, including white blood cell, lymphocyte, granulocyte, monocyte, red blood cell, platelet counts, mean corpuscular volume, and hemoglobin concentration. (W) Representative H&E staining of heart, liver, spleen, lung, and kidney. *n* = 6 mice per group. Scale bar: 50 µm. Individual scatter points represent independent biological replicates, with data presented as mean ± SEM. The quantitative data presented showed no statistically significant differences between groups (*p* > 0.05). Repeated‐measures one‐way ANOVA with Dunnett's post‐hoc test for panels (C–V).

Given that **TTMs** are intended for intravenous administration, we further assessed their hemocompatibility. All tested cell types, including **TTMs**, RAW264.7, Mφ_PD‐L1@N3_ cells caused minimal hemolysis comparable to the PBS control, whereas Triton X‐100 induced complete lysis (Figure 4A), confirming great hemocompatibility suitable for systemic administration. To further assess systemic safety, we evaluated potential immune activation following repeated intravenous injections. Mice received three intravenous injections of PBS, RAW264.7, or **TTMs** at 48‐h intervals (Figure 4B). Serum cytokine analysis showed no significant changes in IL‐1, IL‐2, IL‐6, or TNF‐α levels across groups (Figure S15). ELISA showed IgG and IgM concentrations comparable to controls, indicating low immunogenicity (Figure 4C,D).

To systematically evaluate the systemic safety of the therapeutic regimen, we analyzed serum biochemistry and hematology following three consecutive intravenous injections of **TTMs**. Hepatic (ALT, AST, ALB, TBIL, CHO) and renal (BUN, CREA, UA) function markers remained within normal physiological ranges, showing no significant deviation from controls (Figure 4E–L). Similarly, complete blood counts revealed no hematological toxicity, as evidenced by unaltered leukocyte subsets and red blood cell parameters (Figure 4M–V).

To rigorously assess the long‐term safety margin and potential cumulative toxicity, we extended the administration protocol to 6 and 9 consecutive doses (Figures S16A and S17A). Even under this intensified regimen, mice exhibited no signs of immunogenicity, as evidenced by stable serum IgG and IgM levels (Figures S16B,C and S17B,C). Furthermore, extensive biochemical and hematological profiling confirmed the absence of hepatic or renal impairment even after nine injections (Figures S16D–U and S17D–U).

Critically, histopathological examination of major organs (heart, liver, spleen, lung, and kidney) across all dosing regimens (3, 6, and 9 doses) revealed preserved tissue architecture. Notably, we observed no evidence of pathological macrophage accumulation, granuloma formation, or microthrombi, particularly in the lungs and liver, common sites for cellular entrapment (Figure 4W; Figures S16V and S17V). These data confirm that **TTMs** possess an excellent biosafety profile.

### 2.3 Bioorthogonal Tracking of TTM Migration in Allograft Rejection Models

To visualize **TTM** trafficking during rejection, we employed a bioorthogonal labeling strategy based on strain‐promoted azide–alkyne cycloaddition (SPAAC) [42, 43, 44, 45]. A murine skin transplant rejection model was first established by grafting BALB/c donor skin onto C57BL/6J recipients. Histological analysis confirmed progressive graft rejection, with mild bleeding on day 1, followed by lymphocytic infiltration, epithelial vacuolation, and skin shrinkage by days 3–5, and severe necrosis with dense CD3^+^ T cell infiltration by day 7 (Figure S18). Pathological scoring according to Banff criteria demonstrated no rejection in syngeneic grafts [46], whereas 60% of allografts reached grade 2 by day 3 and >80% progressed to grade 3–4 by day 7 (Figure S19), confirming successful model establishment.

**FIGURE 5:**
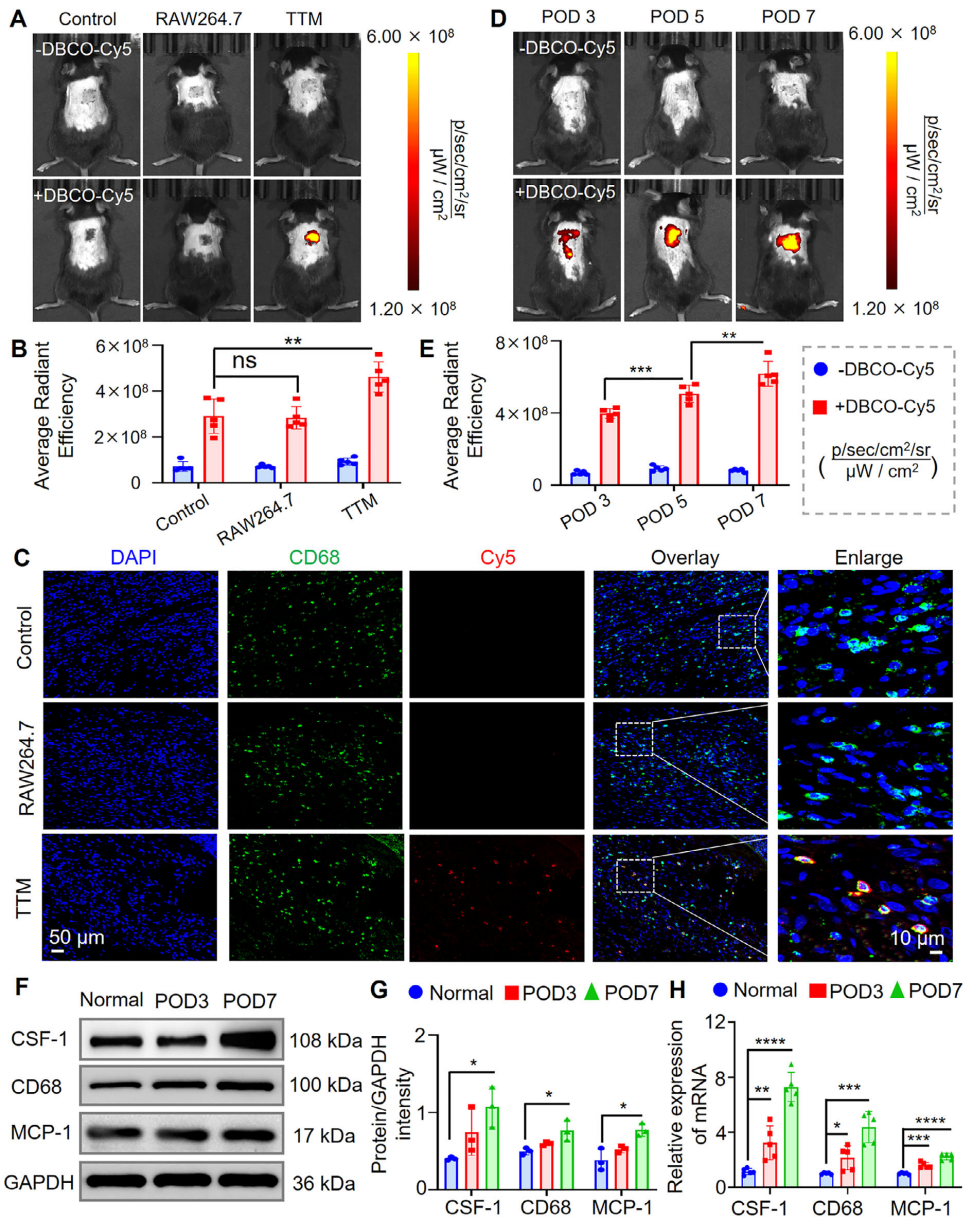
In vivo imaging and graft targeting of **TTMs** in allograft mice. (A‐B) On POD 4, mice were injected with PBS, RAW264.7, or **TTMs**. After 24 h, DBCO‐Cy5 (100 µL, 50 µm) was administered, and fluorescence imaging performed 6 h later; Quantitative analysis showed graft‐localized signals in the **TTM** group. *n* = 5, ns, not significant, **p < 0.01; (C) Immunofluorescence confirmed colocalization of CD68 (green) and Cy5 (red) in grafts, indicating targeted **TTM** migration. Scale bars: 50 µm; enlarged 10 µm; (D‐E) Longitudinal imaging on POD 3, 5, and 7 revealed progressive **TTM** accumulation at grafts. *n* = 5, **p < 0.01, ***p < 0.001; (F–H) Western blot and qPCR of graft tissues showed increased CSF‐1, CD68, and MCP‐1 expression on POD 3 and 7; For WB, three biological replicates were used; for qPCR, *n* = 5. ns, no significant; *p < 0.05, **p < 0.01, ***p < 0.001, ****p < 0.0001. Data are presented as mean ± SEM, where each superimposed scatter point indicates an independent biological replicate. Normally distributed data were subjected to statistical analysis, with panels (B), (E), (G), and (H) specifically evaluated by Student's *t*‐test (for two‐group comparisons) or one‐way ANOVA with Dunnett's post‐hoc test.

To assess in vivo migration, **TTMs** were administered intravenously on postoperative day (POD) 4, followed by DBCO‐Cy5 injection 24 h later. Whole‐body fluorescence imaging demonstrated graft‐localized signals as early as 4 h, peaking at ∼6 h and remaining detectable for up to 48 h (Figures S20 and S21). Although mild nonspecific signals were observed early, fluorescence localized to the graft over time. To test labeling specificity, mice received PBS, RAW264.7, or **TTMs** on POD 4. After 24 h, DBCO‐Cy5 was injected, and imaging was performed 6 h later. Strong graft‐localized fluorescence was detected only in the **TTM** group, while PBS and RAW264.7 showed weak signals (Figure 5A). Quantitative analysis revealed ∼1.6‐fold higher graft fluorescence in the **TTM** group (Figure 5B). Immunofluorescence confirmed Cy5 colocalization with CD68^+^ macrophages in graft tissue (Figure 5C). Additional signals in the liver and lung reflected physiological clearance by Kupffer cells and transient pulmonary retention [47]. This phenomenon is attributable to the intrinsic limitations of intravenous cell infusion. Importantly, these signals represent nonspecific, short‐term retention, rather than directed chemotaxis. Fluorescence enrichment at allograft sites in **TTM**‐treated mice reflects both chemotactic recruitment and selective visualization enabled by azide‐DBCO labeling (Figure S22).

To examine inflammation‐adaptive migration [48], **TTMs** were injected on POD 2, 4, and 6. Following DBCO‐Cy5 administration 24 h later, in vivo imaging revealed graft‐localized fluorescence at all time points, with maximal intensity observed on POD 5, which coincided with peak inflammatory activity (Figure 5D). Quantitative analysis showed a strong correlation between fluorescence intensity and rejection grade (Figure 5E). Western blot analysis of graft tissues showed progressive upregulation of CSF‐1, MCP‐1, and CD68, reaching ∼2.65‐, 2.02‐, and 1.54‐fold increases, respectively, by day 7 (Figure 5F,G). qPCR further confirmed elevated transcripts of CSF‐1 and MCP‐1 (Figure 5H), supporting their involvement in recruitment [49, 50, 51]. These findings demonstrate that **TTMs** preferentially migrate to inflamed grafts, coinciding with the upregulation of chemotactic cues (e.g., MCP‐1, CSF‐1), and can be selectively visualized through bioorthogonal labeling.

Given the substantial accumulation of **TTMs**, we further assessed whether this influx exacerbated acute graft inflammation. **TTMs** were administered on POD 6 during active rejection, and grafts were harvested 24 h later (POD 7) for analysis. Quantification of CD3^+^ T‐cell infiltration, a hallmark of acute cellular rejection, revealed no significant increase in **TTM**‐treated grafts compared to the PBS control (Figure S23). This finding provides strong evidence that the targeted migration of **TTMs** does not induce a local “flare‐up” or recruit additional effector cells, likely due to the concurrent delivery of immunosuppressive payloads.

Finally, to rigorously confirm that the observed graft accumulation was driven by macrophage‐specific chemotaxis rather than passive leakage, we employed dendritic cells (DC2.4), embryonic stem cells (ESCs), and mesenchymal stem cells (MSCs) as comparative controls. All cell types underwent identical surface engineering with AC_4_ManNAz to introduce azide groups, which was verified by confocal microscopy (Figure S24A). As expected, ESCs lacked specific inflammatory homing receptors and were cleared by the reticuloendothelial system, with signals confined to the liver and lungs (Figure S24C). DC2.4 cells preferentially migrated to secondary lymphoid organs (spleen) and the liver/lungs, consistent with their antigen‐presenting trafficking routes [52], rather than the peripheral graft (Figure S24D). While MSCs possess some homing potential, they are subject to significant “pulmonary first‐pass” entrapment following intravenous injection and typically require longer timeframes to migrate [53, 54]. Within the 24‐h window, signals were mainly observed in the lungs and liver, with no significant accumulation at the rejection site (Figure S24E). Quantitative analysis (Figure S25) confirmed that the mean fluorescence intensity in allograft skin was significantly lower in the ESC (4.1 ± 1.4), DC2.4 (4.3 ± 1.1), and MSC (3.9 ± 0.8) groups compared to the **TTM** group (28.4 ± 5.6; units: ×10^7^ [p/sec/cm^2^/sr]/ [µW / cm^2^]). These findings strongly support that intrinsic inflammatory chemotaxis is the primary driver for efficient graft targeting in our system, highlighting a unique advantage of **TTMs** for the targeted delivery of PD‐L1 and rapamycin.

### 2.4 TTM Prolongs Graft Survival in Allograft Mice

**FIGURE 6:**
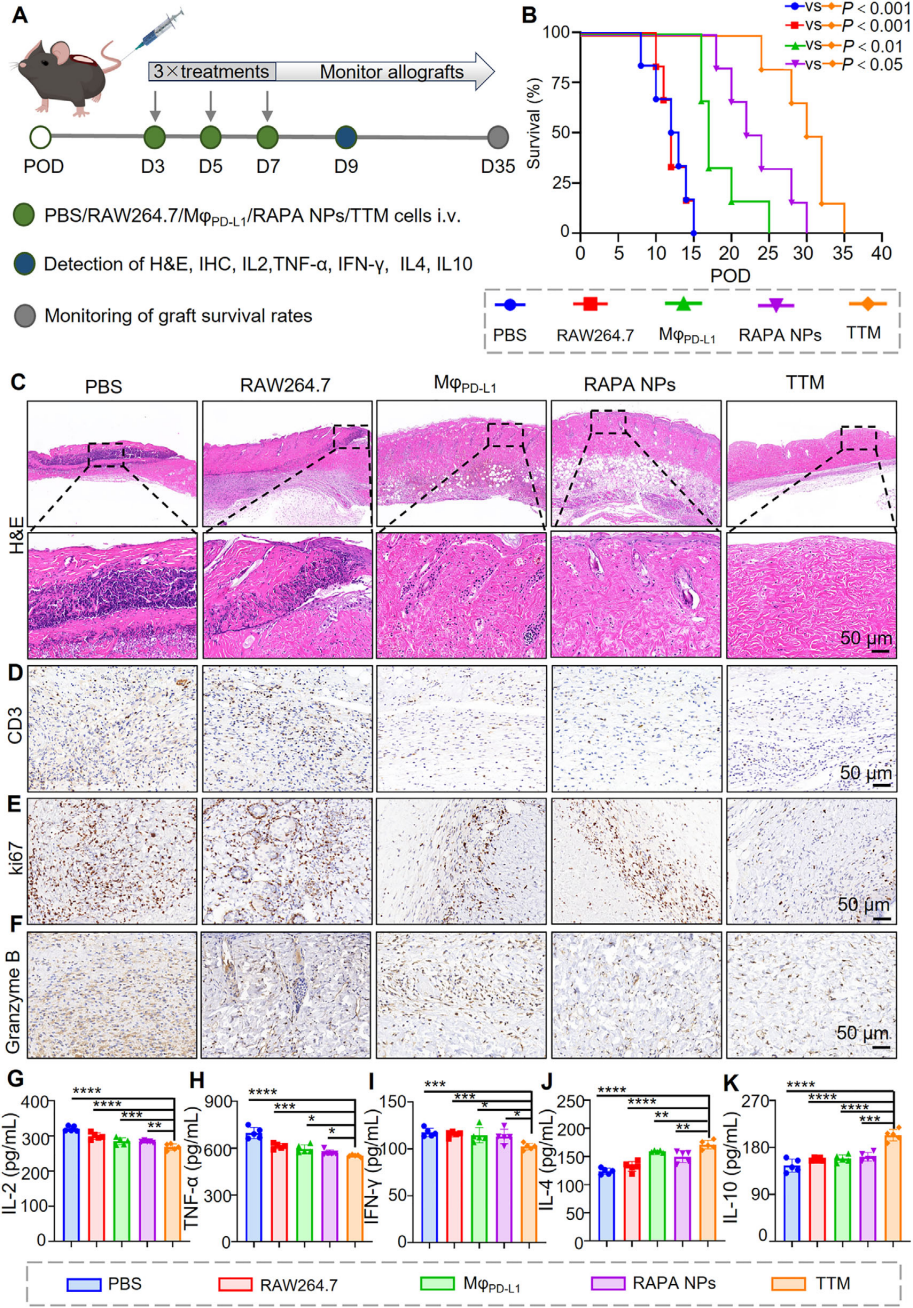
**TTMs** prolong graft survival and modulate immune responses in allograft mice. (A) Experimental timeline for in vivo evaluation of **TTMs**; (B) Kaplan–Meier survival curves showing prolonged graft survival after **TTM** treatment compared with controls. (C–F) Histological and immunohistochemical analysis of grafts on POD 9: H&E, CD3, Ki67, and Granzyme B staining. Scale bars: 50 µm; (G–K) Serum cytokine levels measured by ELISA. **TTMs** reduced IL‐2, TNF‐α, and IFN‐γ, while increasing IL‐4 and IL‐10. *n* = 5 per group, *p < 0.05, **p < 0.01, ***p < 0.001, ****p < 0.0001. Quantitative data are presented as mean ± SEM, with individual scatter points denoting independent biological replicates. For panels (B) and (G–K), normally distributed data were analyzed using Student's *t*‐test (for two‐group comparisons) or one‐way ANOVA with Dunnett's post‐hoc test.

Therapeutic efficacy was evaluated in a murine allogeneic skin graft model. Mice received intravenous injections of PBS, RAW264.7 cells, Mφ_PD‐L1_, RAPA NPs, or **TTMs** starting on postoperative day (POD) 3, repeated every 48 h for a total of three doses. Grafts and sera were collected on POD 9, and graft survival was monitored daily until day 35 (Figure 6A).

At day 6 post‐transplantation, grafts in PBS‐ and RAW264.7‐treated groups exhibited extensive necrosis with pronounced darkening and scab formation. In contrast, Mφ_PD‐L1_ and RAPA NPs treatment resulted in moderate improvement, showing delayed scab formation and reduced tissue contraction. Notably, grafts from **TTM**‐treated mice remained intact, smooth, and pliable, without visible signs of rejection (Figure S26). Kaplan–Meier analysis revealed that **TTM** treatment markedly prolonged graft survival compared with all other groups. Grafts in the PBS and RAW264.7 groups were rejected within 15 days, whereas **TTMs** extended survival up to 35 days, significantly longer than Mφ_PD‐L1_ (*p* < 0.01) and RAPA NPs (*p* < 0.05) (Figure 6B). General health was monitored by body weight. All groups showed mild weight loss between POD 3‐5, likely reflecting postoperative stress, but body weight stabilized by day 9 without group differences (Figure S27).

Histological analysis revealed severe necrosis, dense inflammatory infiltration, and tissue disruption in PBS‐ and RAW264.7‐treated grafts, consistent with acute rejection (Figure 6C). Mφ_PD‐L1_ and RAPA NPs groups showed partial preservation of tissue integrity but retained inflammatory changes. In contrast, grafts from **TTM**‐treated mice exhibited largely preserved architecture with minimal inflammatory cell infiltration. CD3 staining demonstrated abundant T cell infiltration in PBS and RAW264.7 groups, reduced infiltration in Mφ_PD‐L1_ and RAPA NP groups, and minimal CD3^+^ cells in **TTM**‐treated grafts (Figure 6D; Figure S28A). Ki67 staining, an indicator of proliferative activity, showed a parallel trend, with the lowest proliferation in the **TTM** group (Figure 6E; Figure S28B). Granzyme B expression, reflecting cytotoxic T cell activity [55], was markedly suppressed in **TTM**‐treated grafts compared with all other groups (Figure 6F; Figure S28C).

To assess systemic immunomodulation, serum cytokines were quantified by ELISA. **TTM** treatment significantly reduced IL‐2, TNF‐α, and IFN‐γ levels compared with all other groups (Figure 6G–I). Relative to PBS controls, IL‐2, TNF‐α, and IFN‐γ levels in the **TTM** group decreased by ∼16%, ∼21%, and ∼12%, respectively. The degree of suppression was greater than that observed with Mφ_PD‐L1_ or RAPA NPs alone, consistent with combined checkpoint engagement and mTOR inhibition. Anti‐inflammatory cytokine profiling revealed that **TTMs** enhanced IL‐4 and IL‐10 production. IL‐4 levels were increased by ∼38% compared with PBS (Figure 6J), while IL‐10 levels were elevated by ∼40% and were significantly higher than in the Mφ_PD‐L1_ or RAPA NP groups (Figure 6K). These findings demonstrate that **TTM** therapy achieves coordinated regulation of pro‐ and anti‐inflammatory cytokines through the synergistic action of PD‐L1 signaling and mTOR inhibition.

### 2.5 TTMs Suppress Alloreactive T Cell Responses and Promote Regulatory T Cell Induction

**FIGURE 7:**
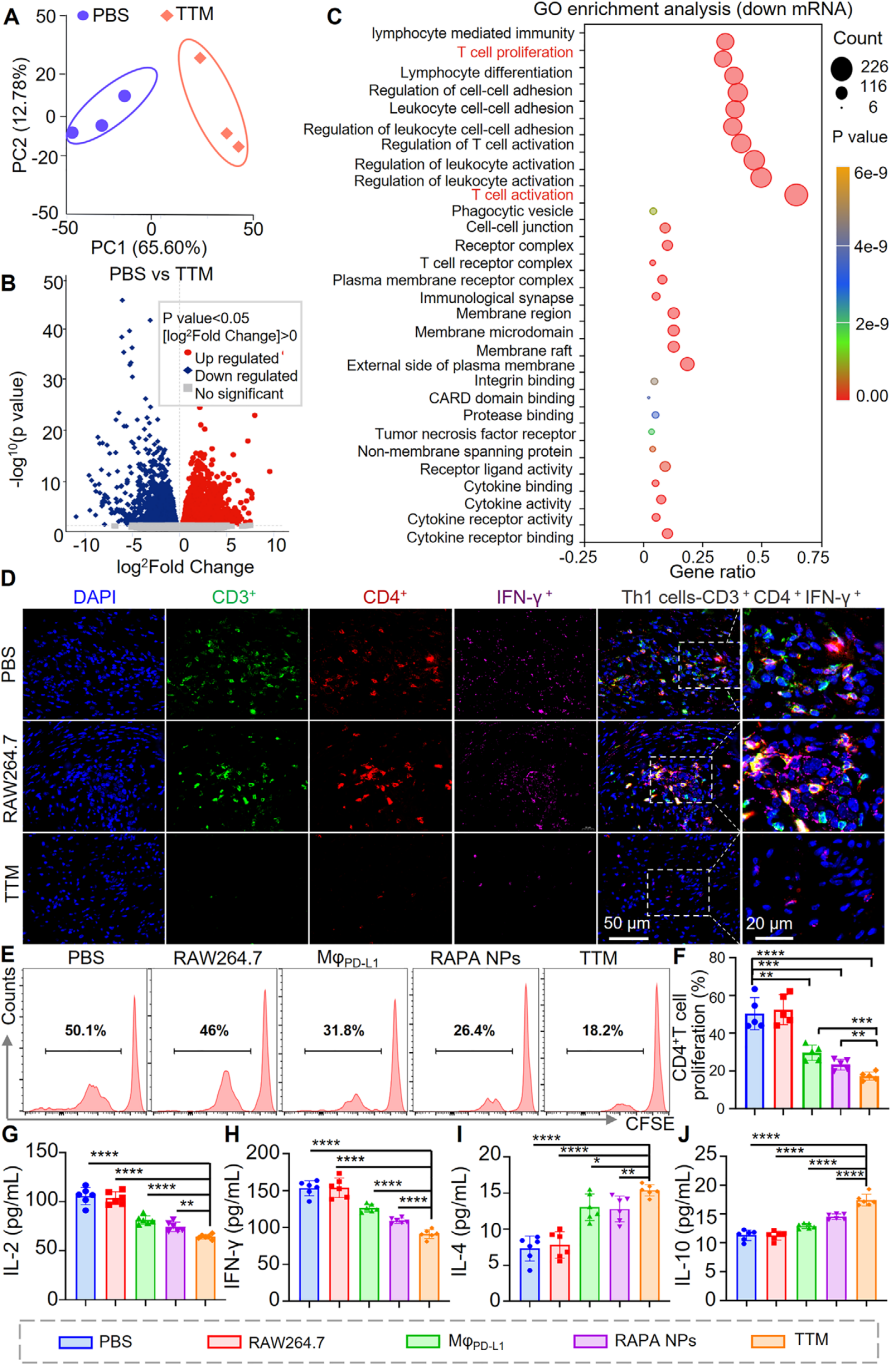
**TTMs** suppress T‐cell activation and proliferation. (A,B) RNA‐seq analysis of grafts from PBS‐ and **TTM**‐treated mice: Principal component analysis (PCA) and Volcano plot of differentially expressed genes (adjusted *p* < 0.05). (C) Gene ontology (GO) enrichment downregulated pathways revealed suppression of T‐cell activation and proliferation; (D) Immunofluorescence of grafts on POD 9 showed reduced Th1 infiltration (CD3^+^CD4^+^IFN‐γ^+^) after **TTM** treatment. Images of Mφ_PD‐L1_ and RAPA NPs groups are presented in Figure S31. Scale bar: 50 µm; (E,F) Flow cytometry of CFSE‐labeled CD4^+^ T cells demonstrated reduced proliferation in co‐culture with **TTMs**; (G–J) ELISA of co‐culture supernatants showed decreased IL‐2 and IFN‐γ, and increased IL‐4 and IL‐10 in the **TTM** group. *n* = 5, *p < 0.05, **p < 0.01, ***p < 0.001, ****p < 0.0001. For panels (F–J), normally distributed quantitative data were analyzed using Student's *t*‐test (for two‐group comparisons) or one‐way ANOVA with Dunnett's post‐hoc test. All quantitative results are presented as mean ± SEM, with individual scatter points denoting independent biological replicates.

To investigate how **TTMs** influence alloreactive T cell responses during rejection, we conducted transcriptomic profiling of skin grafts harvested on postoperative day 9 (POD 9) from **TTM**‐ and PBS‐treated mice. RNA sequencing revealed distinct expression patterns between groups, as shown by Pearson correlation heatmaps and principal component analysis (PCA), with the first component accounting for 65.6% of variance and clearly separating the two cohorts (Figure 7A; Figure S29). Differential expression analysis identified extensive transcriptional changes induced by **TTM** therapy (Figure 7B). Gene ontology (GO) enrichment analysis indicated significant downregulation of pathways associated with T cell activation, proliferation, and effector function (Figure 7C), suggesting attenuated transcriptional activity of alloreactive T cells.

Given the central role of CD4^+^ Th1 cells in driving graft rejection [4, 56, 57], we next examined whether **TTM** treatment affected Th1 differentiation and infiltration. RNA‐seq analysis further revealed marked downregulation of key transcription factors that govern Th1 lineage differentiation [58, 59], including STAT1, STAT4, and T‐bet (Figure S30). Consistent with these transcriptional changes, immunofluorescence staining for CD3, CD4, and IFN‐γ demonstrated that **TTM** treatment markedly reduced Th1 cell infiltration within grafts compared with PBS‐ and RAW264.7‐treated groups, whereas Mφ_PD‐L1_ and RAPA NPs produced only modest effects (Figure 7D; Figure S31).

To further evaluate the regulatory effect of **TTMs** on CD4^+^ T cell proliferation, we conducted in vitro co‐culture assays. CD4^+^ T cells were isolated from mouse spleens via magnetic bead separation, and their purity was subsequently verified by flow cytometry to be 95.2% (Figure S32). Subsequent co‐culture of these CD4^+^ T cells with **TTMs** demonstrated that **TTMs** significantly suppressed CD4^+^ T‐cell expansion in a dose‐dependent manner, achieving ∼62% inhibition at a 1:4 ratio (Figure S33). Flow cytometric analysis confirmed that **TTMs** exhibited stronger inhibitory effects on CD4^+^ T‐cell proliferation compared with Mφ_PD‐L1_ (*p*< 0.001) and RAPA NPs (*p* < 0.01) (Figure 7E,F). Cytokine analysis of co‐culture supernatants revealed that **TTMs** decreased IL‐2 and IFN‐γ production while increasing IL‐4 and IL‐10 (Figure 7G–J), consistent with a shift toward an anti‐inflammatory cytokine profile.

**FIGURE 8:**
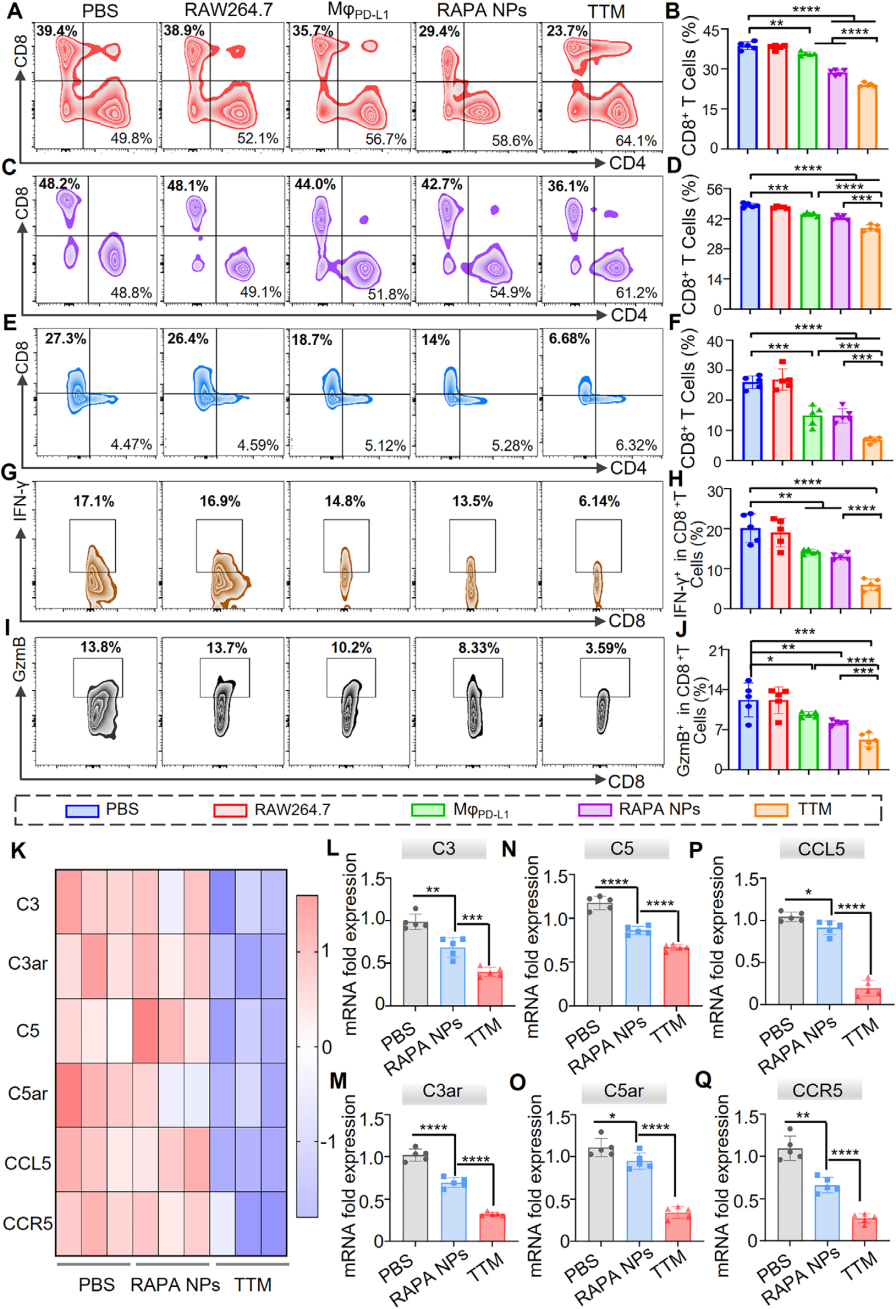
**TTMs** reduce CD8^+^ T‐cell infiltration and suppress complement and chemokine signaling in grafts. (A,B) Flow cytometry plots and quantification of CD8^+^ T‐cell frequencies in spleens; (C,D) Corresponding analysis of CD8^+^ T cell frequencies in lymph nodes; (E,F) flow cytometry plots and quantification of CD8^+^ T cell frequencies in allograft skin; (G,H) Corresponding analysis of CD8^+^IFN‐γ^+^ T cell frequencies in allograft skin; (I,J) Analysis of CD8^+^GzmB^+^ T cell frequencies in allograft skin. In the same flow cytometry experiment, identical voltage settings were used to sort cells from the PBS, RAW264.7, Mφ_PD‐L1_, RAPA NPs, and **TTM** groups. Gating strategy is shown in Figures S35–S39; (K) Heatmap of RNA‐seq data showing relative expression of complement components (C3, C3ar, C5, C5ar) and chemokine axis genes (CCL5, CCR5) in skin grafts on POD 9 (*n* = 3 biological replicates per group); (L–Q) qPCR analysis confirming mRNA expression levels of selected targets, normalized to GAPDH. n = 5 mice per group. Data are shown as mean ± SD. *p < 0.05, **p < 0.01, ***p < 0.001, ****p < 0.0001. Normally distributed data were subjected to statistical analysis, specifically evaluated by Student's *t*‐test (for two‐group comparisons) or one‐way ANOVA with Dunnett's post‐hoc test.

Since Th1‐derived IFN‐γ promotes CD8^+^ T cell activation and cytotoxicity, we next examined CD8^+^ T cell infiltration in graft tissues and secondary lymphoid organs on POD 9. Immunofluorescence staining showed reduced CD8^+^ T cell accumulation in **TTM**‐treated grafts compared with PBS, RAW264.7, Mφ_PD‐L1_, and RAPA NPs groups (Figure S34). Flow cytometric analysis of spleens demonstrated a decrease in CD8^+^ T cell frequency to 24.3% in the **TTM** group, compared with 35.7% in Mφ_PD‐L1_ and 28.6% in RAPA NPs (Figure S35; Figure 8A,B)**. **Consistent reductions were observed in lymph nodes, where **TTMs** yielded the lowest CD8^+^ T cell frequencies among groups (Figure S36; Figure 8C,D). We further analyzed graft‐infiltrating lymphocytes. Despite the lower proportion of lymphocytes in skin tissue relative to spleen or lymph nodes, flow cytometry revealed reduced CD8^+^ T cell infiltration after **TTM** treatment. Quantitative analysis showed reductions of 54.22% and 53.91% relative to Mφ_PD‐L1_ and RAPA NPs, respectively (Figure S37; Figure 8E,F), consistent with findings in secondary lymphoid organs. Flow cytometric analysis showed that **TTMs** reduced the proportions of CD8^+^IFN‐γ^+^ and CD8^+^GzmB^+^ T cells to 5.99% ± 1.46% and 4.56% ± 1.0%, respectively, whereas Mφ_PD‐L1_ and RAPA NPs produced only partial reductions (Figures S38 and S39; Figure 8G–J). These results indicate that **TTMs** reduce both systemic and local CD8^+^ T‐cell infiltration and diminish their activation status, which may collectively alleviate cytotoxic responses within graft tissues.

To further elucidate the molecular mechanisms underlying this immunoregulatory effect, we next analyzed signaling pathways associated with complement activation and PI3K/Akt/mTOR regulation. Transcriptomic analyses on POD 9 showed that **TTMs** reduced IL‐2, IFN‐γ, and TNF‐α while increasing IL‐4 and IL‐10 compared with PBS and RAPA NP groups (Figure S40). As IFN‐γ and TNF‐α are known to promote complement activation via upregulation of C3 and C5 expression [60, 61, 62], we further examined complement signaling. Transcriptomic and qPCR analyses demonstrated downregulation of C3, C5, C3ar, and C5ar in **TTM**‐treated grafts (Figure 8K–O). suggesting attenuation of complement‐mediated injury [63, 64].

KEGG pathway enrichment analysis showed that, compared with RAPA NPs treatment, differentially expressed genes in the **TTM** group were significantly enriched in the PI3K/Akt signaling pathway (Figure S41A), which is closely associated with mTOR regulation [65, 66, 67]. Western blotting confirmed reduced phosphorylation of PI3K, Akt, and mTOR in **TTM**‐treated grafts, whereas total protein levels were unchanged, indicating pathway modulation at the level of phosphorylation (Figure S41B). To further test the link between complement activity and mTOR signaling, a rescue experiment with the complement activator lipoteichoic acid (LTA) was performed [68]. Compared with **TTM** + LTA, the **TTM** group showed lower phosphorylation of PI3K (59.9% reduction), Akt (45.78% reduction), and mTOR (21.56% reduction), with all comparisons reaching statistical significance (*p*< 0.01) (Figure S41C–E). These results suggest that **TTMs** attenuate complement activation and inhibit PI3K/Akt/mTOR phosphorylation, thereby reinforcing rapamycin‐mediated mTOR blockade.

We next examined whether **TTM** therapy influences immune cell trafficking by assessing the chemokine CCL5 and its receptor CCR5, which mediate T cell recruitment to inflamed tissues [69, 70, 71, 72]. Transcriptomic and qPCR analyses of grafts harvested on POD 9 showed reduced expression of both genes in **TTM**‐treated mice compared with PBS and RAPA NPs groups (Figure 8K,P,Q). Relative to RAPA NPs, CCL5 and CCR5 expression decreased by 78.6% and 59.5%, respectively. These findings indicate that **TTMs** mitigate chemokine signaling involved in T‐cell recruitment, which may consequently reduce T‐cell activation and proliferation within grafts [73].

**FIGURE 9:**
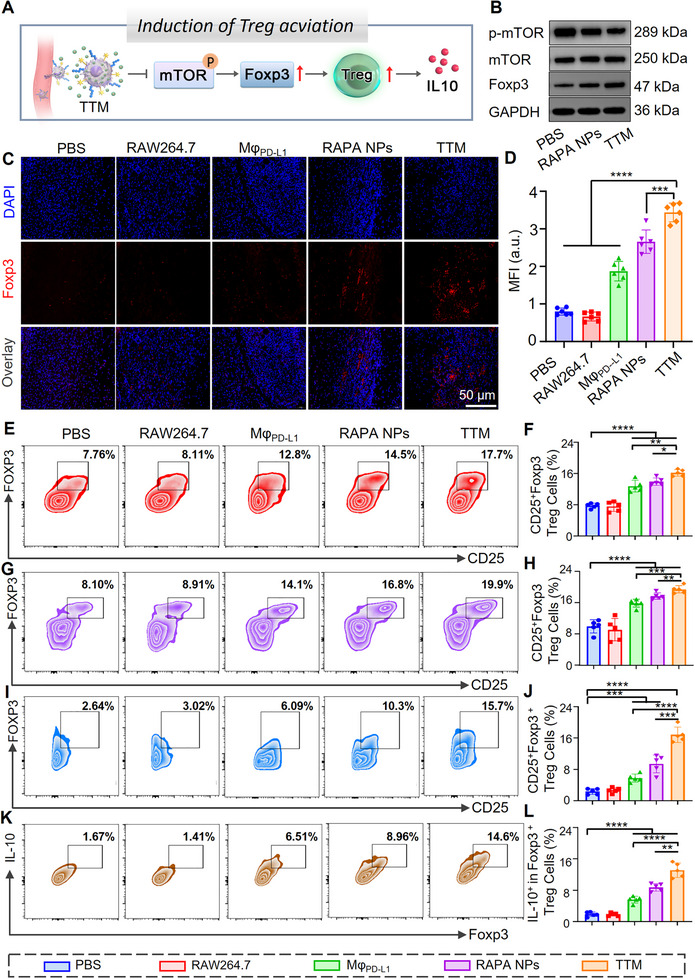
**TTMs** promote Treg differentiation and function in vivo. (A) Schematic diagram illustrating **TTM**‐induced Treg activation; (B) Western blot of mTOR, p‐mTOR, and Foxp3 expression in graft tissues; (C‐D) Immunofluorescence staining and quantification of Foxp3^+^ cells in grafts. Scale bar: 50 µm. Mean fluorescence intensity (MFI) was quantified using ImageJ (Fiji 2.15.0), *n *= 3 per sample; (E,F) Flow cytometry analysis of CD25^+^Foxp3^+^ Tregs in spleens; (G,H) Flow cytometry analysis of the proportion of CD25^+^Foxp3^+^ Treg cells in lymph nodes; (I,J) CD25^+^Foxp3^+^ Treg frequencies in graft tissues; (K,L) Frequencies of Foxp3^+^IL‐10^+^ Tregs in graft tissues. In all flow cytometry experiments, consistent voltage settings were applied for cell sorting, with detailed gating strategies for cells from different sources provided in Figures S43–S46. Statistical analysis for normally distributed data was conducted using Student's *t*‐test (for two‐group comparisons) or one‐way ANOVA with Dunnett's post‐hoc test. All data are presented as mean ± SEM. Data represent *n* = 5 biological replicates, with statistical significance denoted as **p < 0.01, ***p < 0.001, ****p < 0.0001.

To further examine the role of **TTMs** in promoting graft tolerance, we assessed their effects on Treg differentiation and function. Western blot analysis showed that, relative to the RAPA NP group, **TTMs** reduced p‐mTOR expression by 50.7% and increased the Treg transcription factor Foxp3 by 1.6‐fold (Figure 9B; Figure S42), providing a molecular basis for Treg induction. Immunofluorescence staining revealed a significant accumulation of Foxp3^+^ cells in the allograft tissues treated with **TTMs** on POD 9 (Figure 9C). This finding was further supported by semi‐quantitative analysis of average fluorescence intensity (Figure 9D). Given the central role of Foxp3 in defining Treg identity and suppressive capacity [74, 75], these results suggest that **TTM** treatment enhances local Treg‐mediated immunoregulation within grafts. Flow cytometric analysis of spleens and lymph nodes further demonstrated increased CD25^+^Foxp3^+^ Treg frequencies in **TTM**‐treated mice compared with RAPA NPs (Figures S43 and S44; Figure 9E–H). Single‐cell suspensions prepared from allograft skin tissue confirmed a 1.79‐fold increase in intra‐graft Tregs in the **TTM** group relative to RAPA NPs (Figure 9I,J; Figure S45). These observations are consistent with the known ability of rapamycin to promote Foxp3 induction and the role of PD‐L1 in stabilizing Tregs under inflammatory conditions [76]. Finally, functional assessment of Foxp3^+^IL‐10^+^ Tregs revealed that **TTMs** increased IL‐10^+^ Treg proportions by 2.3‐fold compared with Mφ_PD‐L1_ and by 1.5‐fold compared with RAPA (Figure 9K,L; Figure S46), indicating enhanced Treg suppressive capacity. Collectively, these results show that **TTMs**, through the combined effects of PD‐L1 signaling and rapamycin‐mediated mTOR inhibition, increase Foxp3 expression, expand Treg populations, and enhance their immunosuppressive function (Figure 9A), thereby contributing to the establishment of a tolerogenic graft microenvironment.

## 3 Discussion

In this study, we developed a macrophage‐based therapeutic platform, trackable tolerogenic macrophages (**TTMs**), to achieve precise and localized immunoregulation in transplantation. **TTMs** were constructed by inducing PD‐L1 expression, incorporating azide groups via metabolic labeling for bioorthogonal tracking, and loading intracellular rapamycin nanoparticles to deliver sustained immunosuppression. Following systemic administration, **TTMs** selectively migrated to inflamed grafts, where bioorthogonal click labeling enabled real‐time visualization of their accumulation. Through combined checkpoint engagement and mTOR inhibition, **TTMs** effectively mitigated alloimmune activation. This work establishes a proof‐of‐concept for engineered macrophages as multifunctional therapeutic cells integrating graft homing, molecular tracking, and dual‐pathway immune modulation to promote transplant tolerance.

Macrophages were selected as therapeutic carriers because of their intrinsic properties, including active homing to inflamed grafts, robust phagocytic capacity for drug loading, and extended in vivo persistence. Although dendritic cells (DCs) and mesenchymal stem cells (MSCs) have been investigated for tolerance induction, both exhibit limitations [77, 78]. DCs preferentially migrate to lymphoid tissues and require continuous exposure to IL‐10 or TGF‐β to maintain a tolerogenic phenotype, rendering them less effective for direct modulation within the graft microenvironment [58, 79, 80]. MSCs exhibit limited homing to sites of injury, including grafts, and both MSCs and DCs are rapidly cleared following systemic infusion (typically <2 weeks), restricting their therapeutic duration [81, 82, 83]. In contrast, adoptively transferred macrophages survive for 2–4 weeks in vivo [31], and can be loaded intracellularly with bioactive payloads, offering a dual‐function platform for targeted drug delivery and immunomodulation [84]. In this study, engineered macrophages retained chemotactic migration toward grafts via MCP‐1 and CSF‐1 signaling, while bioorthogonal labeling enabled real‐time visualization of their recruitment (Figure 5A–E). While we observed a correlation between graft chemokine expression (MCP‐1, CSF‐1) and **TTM** accumulation, confirming the precise homing mechanism would require further investigation using chemokine receptor knockout models. Nevertheless, under chemokine‐mediated recruitment, **TTM** exhibited excellent graft homing capabilities compared to ESCs, DC2.4 cells, and MSCs. This exceptional targeting performance, characteristic of macrophage‐based strategies like **TTM**, along with the inherent programmability of macrophages, collectively underscores their feasibility as multifunctional therapeutic cells.

Since macrophages are terminally differentiated, we adopted a non‐genetic strategy to induce PD‐L1 via the IFN‐γ/JAK‐STAT signaling cascade [85, 86]. A critical consideration for clinical translation is the durability of this phenotype. Our time‐course analysis demonstrated that PD‐L1 expression is sustained at high levels for at least 48 h post‐induction before declining (Figure S10). This empirically determined stability window supports the rationale for our in vivo administration regimen (every 48 h), ensuring continuous checkpoint signaling within the graft. Notably, while IFN‐γ induces STAT1 phosphorylation, RNA‐seq of graft tissues revealed reduced STAT1 transcript abundance, an apparent discrepancy explained by post‐translational activation of STAT1 and the heterogeneous nature of bulk transcriptomes. Feedback inhibition through SOCS family proteins may further constrain STAT1 transcription [87, 88]. These findings highlight the complexity of STAT1 regulation in vivo and point to the need for single‐cell transcriptomic analysis to delineate macrophage‐specific dynamics.

To enable real‐time imaging, **TTMs** were labeled with azide groups via metabolic incorporation of AC_4_ManNAz, followed by in vivo click conjugation with DBCO‐Cy5. This bioorthogonal chemistry provided specific, biocompatible fluorescent labeling without compromising cell function [43, 89]. Compared to conventional membrane or intracellular dyes, this approach achieved higher specificity, lower cytotoxicity, and allowed dynamic visualization of macrophage trafficking. **TTMs** preferentially accumulated in rejecting grafts, and fluorescence intensity correlated with inflammatory severity, validating the strategy for non‐invasive monitoring of therapeutic cell migration. Beyond this application, such labeling techniques may inform future development of clinically relevant imaging modalities to track cell therapies in real time.

Macrophages are inherently plastic and can adopt pro‐inflammatory phenotypes under cytokine stimulation, posing potential risks for exacerbating tissue injury. We therefore evaluated **TTM** polarization in vitro. Upon LPS exposure, **TTMs** did not upregulate the M1 marker CD86 but rather showed reduced expression, suggesting resistance to inflammatory activation. This may result from controlled intracellular release of rapamycin, which inhibits mTOR signaling and induces autophagy, thereby limiting macrophage‐driven inflammation [40, 41]. In parallel, PD‐L1 engagement with PD‐1 on T cells provided an extrinsic checkpoint, suppressing T‐cell effector activity and promoting regulatory T‐cell (Treg) differentiation, consistent with elevated Foxp3 expression observed in **TTM**‐treated grafts (Figure 9A,B). Treg‐derived IL‐10, which was increased in **TTM** co‐cultures (Figure 9K), further suppresses M1 polarization and reinforces macrophage tolerance, forming a reciprocal feedback loop that stabilizes the immunoregulatory state. Together, these interactions suggest that **TTMs** couple intrinsic inhibition of macrophage inflammation with extrinsic modulation of T‐cell responses to promote graft homeostasis.

Mechanistically, **TTMs** mediate immune regulation through coordinated actions of PD‐L1 and rapamycin. PD‐L1/PD‐1 ligation attenuates proximal TCR signaling, restricting Th1 differentiation and reducing effector cytokines such as IFN‐γ and TNF‐α. Concurrently, rapamycin released from **TTMs** sustains inhibition of the mTOR pathway, suppressing metabolic and proliferative programs of CD4^+^ and CD8^+^ T cells. By targeting complementary checkpoints, membrane signaling, and intracellular metabolism, PD‐L1 and rapamycin achieve synergistic suppression of alloimmune activation. This dual regulation was accompanied by broad modulation of the inflammatory milieu. Transcriptomic and qPCR analyses showed downregulation of complement components (C3, C5, C3aR, C5aR) and decreased phosphorylation of PI3K, Akt, and mTOR, suggesting dampened amplification loops that sustain effector T‐cell responses. Chemokine axis analysis revealed reduced CCL5 and CCR5 expression, limiting T‐cell recruitment into grafts. These changes coincided with decreased IL‐2, IFN‐γ, and TNF‐α, along with elevated IL‐4 and IL‐10, collectively favoring Foxp3^+^ Treg differentiation and IL‐10‐producing Tregs. Thus, **TTMs** orchestrate a multilayered immunoregulatory network that suppresses effector T‐cell activation, proliferation, and migration while reinforcing regulatory pathways, culminating in a tolerogenic graft microenvironment.

Current immunomodulatory strategies include localized delivery systems, such as FasL‐engineered microgels, which have successfully promoted tolerance in islet transplantation by creating a local immune‐privileged site [90, 91, 92]. However, for vascularized composite allografts like skin, localized therapies present challenges; direct injection or material implantation at the graft interface may physically disrupt the critical early‐stage revascularization process. Furthermore, skin allografts elicit potent systemic sensitization involving secondary lymphoid organs. In this context, the intravenous administration of **TTMs** offers a distinct advantage: it avoids mechanical interference with graft healing while allowing for simultaneous graft targeting (via chemotaxis) and systemic immunoregulation. As evidenced by our data, **TTMs** not only resolve local inflammation but also reprogram immune responses in the spleen and lymph nodes. Based on the demonstrated safety of repeated dosing (up to 9 injections), we envision **TTMs** as an induction therapy administered intensively in the early post‐transplant phase to establish systemic tolerance, offering a translatable strategy that balances potent immunomodulation with long‐term safety.

This proof‐of‐concept study was conducted using a murine macrophage cell line, which may not fully represent autologous primary macrophages intended for clinical use. As with other cell‐based products such as Mreg and Tregs [93, 94, 95, 96], issues of manufacturing consistency and scalability will need to be addressed. Further validation in large‐animal models of vascularized organ transplantation will be required to assess translatability.

## 4 Conclusion

This study establishes trackable tolerogenic macrophages (**TTMs**) as a multifunctional cell therapy integrating checkpoint signaling, metabolic inhibition, and molecular tracking to achieve localized immunoregulation in transplantation. By combining PD‐L1–mediated suppression with rapamycin‐induced mTOR blockade, **TTMs** effectively reprogrammed effector and regulatory T‐cell responses, mitigating alloimmune injury. The incorporation of bioorthogonal labeling further enabled precise monitoring of in vivo cell dynamics. Collectively, these findings provide a mechanistic and translational foundation for developing macrophage‐based immunotherapies with tunable and traceable immune control.

## 5 Experimental Section

### 5.1 Materials

AC_4_ManNAz (900917) and DBCO‐Cy5 (777374) were bought from Sigma–Aldrich (St. Louis, MO, USA). Calcein/PI Cell Viability and Cytotoxicity Detection Kit (C2015M), Reactive Oxygen Species Detection Kit (ROS, S0033S), IP(P0013), and 4′,6‐diamidino‐2‐phenylindole (DAPI, C1006) were obtained from Beyotime Biotechnology (Nanjing, China). Rapamycin (HY‐10219), MCP‐1(HY‐P7764), GM‐CSF(HY‐P7361), and IFN‐γ (P01580) were acquired from MedChemExpress (Monmouth Junction, NJ, USA). Hematoxylin was procured from Servicebio Technology Co., ltd in China. CellMask Plasma Membrane Stains (C37608) were bought from Thermo Fisher Scientific (USA). EasySep Mouse CD4^+^T Cell Isolation Kit (19852) was bought from STEMCELL Technologies (Canada). The Mouse Embryonic Stem Cell Serum‐Free Medium Type I (OriCell MUXES‐90062‐200), Mouse Embryonic Stem Cell Serum‐Free Medium Type II (OriCellMUXES‐90061‐200), Mouse Embryonic Fibroblast Complete Medium (OriCell MUXEF‐90011), and Gelatin Solution (GLT‐11301) were purchased from Cyagen Biosciences (Suzhou) Co., Ltd (China). The Mouse Mesenchymal Stem Cell Specific Medium was obtained from Warner Biology Co., Ltd (China). The cell adhesion substrate (C1010) was purchased from Beijing APPLYGEN Gene Technology Co., Ltd (China). The ELISA kits for IL‐1, IL‐2, IL‐4, IL‐6, IFN‐γ, TNF‐α, IL‐10, IgG, and IgM were sourced from Mlbio Biotechnology Co., Ltd., located in China. The detailed information regarding the antibody reagents required for the study is listed in Table S1. All experiments were conducted using solvents and reagents of analytical grade.

### 5.2 Cell Culture

RAW264.7 cell lines were purchased from the Chinese Academy of Sciences Cell Bank and cultured in Dulbecco's Modified Eagle Medium (DMEM, Gibco). DMEM medium was supplemented with 10% fetal bovine serum (Gibco, Australia, NY) and 1% penicillin–streptomycin (Gibco) to prepare a complete medium. The cells were incubated at 37°C in an environment with 5% CO_2_ (Thermo Fisher Scientific, USA).

**ESCs**. Mouse embryonic stem cells (ESCs) were purchased from Cyagen Biosciences (Suzhou) Co., Ltd. After thawing, ESCs were cultured on a feeder layer of inactivated mouse embryonic fibroblasts (MEF) using ESC‐specific culture medium (OriCellMUXEF‐90011). To ensure the stability of cell proliferation, serum‐free medium Type I (OriCellMUXES‐90062‐200) and Type II (OriCellMUXES‐90061‐200) were used for adaptive transition during passaging. All ESC cultures were conducted in 6 cm culture dishes coated with 0.1% gelatin to promote proper cell attachment. After the cells reached the logarithmic growth phase, they were collected for subsequent experiments.

**DC2.4**. Mouse DC2.4 cells (purchased from Warner Biology Co., Ltd.) were used following mycoplasma testing and Short Tandem Repeat (STR) identification. The cells were routinely cultured in RPMI‐1640 medium supplemented with 10% fetal bovine serum (Gibco) and 10 ng/mL granulocyte–macrophage colony‐stimulating factor (GM‐CSF) to continuously induce and maintain their activity.

**MSCs**. Mouse bone marrow‐derived mesenchymal stem cells (MSCs) were purchased from Warner Biology Co., Ltd. and have passed mycoplasma testing. MSCs were expanded in vitro using a dedicated stem cell culture medium, and passaging was performed once the cells reached the logarithmic growth phase. All subsequent experiments were conducted using MSCs with fewer than 5 passages.

### 5.3 Preparation of Mφ_N3_, Azide‐Modified ESC/DC2.4/MSC Cells, Mφ_PD‐L1_, and Mφ_PD‐L1@N3_

- **Mφ_N3._** RAW264.7 cells were plated in confocal dishes at a concentration of 5 × 10^4^ cells/mL and incubated for 8 h at 37°C in a cell culture incubator. Next, the cells were exposed to complete culture medium supplemented with 30, 50, and 150 µm of AC_4_ManNAz, and then cultured at 37°C in the incubator for an additional 48 h.
- **Azide‐Modified ESC/DC2.4/MSC Cells**. ESC, DC2.4, and MSC cells were plated at a density of 5 × 10^4^ cells/mL in confocal dishes. Given the lower adhesion capability of DC2.4 and ESC cells, the dishes were pre‐coated with a cell adhesion enhancer before seeding. After an 8‐h incubation at 37°C, the cells were collected, and the culture medium was replaced with fresh medium containing 50 µm AC_4_ManNAz. These cells then underwent continuous cultivation for an extra 48 h.
- **Mφ_PD‐L1._** RAW264.7 cells were plated on cover slips at a concentration of 1 × 10^5^ cells/mL and incubated overnight at 37°C in a cell culture incubator. Following this, the culture medium in each well was discarded and replaced with fresh medium containing IFN‐γ at concentrations of 50 ng/mL, 100 ng/mL, and 200 ng/mL. The cells were subsequently incubated for an additional 48 h at 37°C in the cell culture incubator.
- **Mφ_PD‐L1@N3._** RAW264.7 cells were seeded at a concentration of 1 × 10^5^ cells/mL. Following an 8‐h incubation, the cells were exposed to a mixture containing 50 µm AC_4_ManNAz and 100 ng/mL IFN‐γ. This treatment lasted for 48 h before being concluded.

### 5.4 Immunofluorescence Analysis of PD‐L1 Overexpression

RAW264.7 cells were first exposed to IFN‐γ and subsequently fixed with 4% paraformaldehyde for 10 min. Next, a 0.1% Triton X‐100 solution was introduced to the wells to permeabilize the cells for 5 min, allowing for antibody access. After performing three washes with PBS, the cells were treated with 5% BSA at room temperature for 1 h to decrease nonspecific binding. The PD‐L1 antibody was diluted in PBS at a ratio of 1:200 to prepare the primary antibody solution, which was then applied to the cells after removing the blocking solution. The wells were covered with parafilm and incubated overnight at 4°C in the absence of light.

Following sufficient binding of the primary antibody (confirming the incubation time if applicable), the antibody solution was carefully removed using a pipette. The wells were washed with PBS three times to eliminate unbound antibodies. Next, a FITC‐conjugated secondary antibody solution, diluted 1:1000, was added and cultured at room temperature for 1 h. Afterward, DAPI staining solution was added for 10 min in the dark. Coverslips were carefully placed on slides pre‐treated with an anti‐fade reagent, and the slides were sealed. Finally, images were collected via a confocal laser scanning microscope with the following parameters: DAPI: Ex/Em = 405 nm/475 nm; FITC: Ex/Em = 495 nm/520 nm.

### 5.5 Optimization of the Labeling Time for Azide‐Derivative Mannosamine Metabolic Tagging in RAW264.7 Cells

After digesting and counting RAW264.7 cells, they were plated in confocal dishes at a density of 5 × 10^4^ cells/mL or in 96‐well plates at 2 × 10^4^ cells/mL and incubated overnight at 37°C. And the culture medium was then aspirated using a pipette. For the control group, cells underwent medium replacement at 0 h, while the experimental group was treated with complete medium containing 50 µm AC_4_ManNAz. Incubation times for the experimental group were 24 h, 48 h, and 72 h. Following the incubation periods, cells from each group were collected, washed with PBS, and incubated in serum‐free medium with a final concentration of 30 µm DBCO‐Cy5 at 37°C for 30 min before conducting further analyses:

- **Confocal Laser Scanning Microscope (CLSM) Detection**. Cells within each confocal dish were first washed with PBS to remove any unbound substances, followed by fixation in 4% paraformaldehyde for a duration of 10 min. Next, 0.1 mL of DAPI was added to the dish to stain for 10 min. Finally, images were collected using CLSM.
- **Flow Cytometry (FCM) Analysis**. Following the removal of the plates, each well was subjected to three washes with 0.2 mL of PBS to remove any residual medium and unbound materials. Subsequently, cells were digested and collected. After completely removing the culture medium, the cells were dispersed in 0.3 mL of PBS, thoroughly mixed, and filtered through a 300‐mesh cell strainer. The cell suspension was then transferred to FCM tubes and mixed again with a pipette before analysis. The voltage threshold for flow cytometry was adjusted, and cells were further analyzed based on Cy5 fluorescence labeling.
- **Quantification Using a Microplate Reader**: Initially, the DBCO‐Cy5 staining solution was removed from the 96‐well plate. To remove any unbound DBCO‐Cy5, the wells were rinsed three times with PBS. Next, 0.1 mL of PBS was introduced into each well, and the 96‐well plate was placed in the microplate reader. Finally, the fluorescence intensity of the cells was detected for quantitative analysis.

### 5.6 CLSM Verification of Azide on the Surface of RAW264.7's Membranes

RAW264.7 cells were seeded into confocal dishes at a concentration of 5 × 10^4^ cells/mL and incubated at 37°C for 8 h. Afterward, the medium was exchanged for complete medium supplemented with 50 µm AC_4_ManNAz, and the cells were allowed to induce for 48 h. After this step, the medium was discarded, and the cells were rinsed with PBS. They were then incubated with a 30 µm DBCO‐Cy5 staining solution at 37°C in the dark for 30 min. Each dish was subsequently rinsed three times with 2 mL of PBS for 1 min per wash to eliminate any excess staining solution. After diluting the CellMask probe 1000‐fold in PBS was applied to the dish. And the cells were then cultured at 37°C for 10 min in the dark. At last, the staining solution was removed with a pipette, after which the cells were counterstained with DAPI for a duration of 10 min. Multicolor fluorescence laser confocal microscopy was utilized to observe the cells. The excitation and emission wavelengths were adjusted for DAPI (Ex/Em = 405 nm/475 nm), Cy5 (Ex/Em = 650 nm/670 nm), and CellMask (Ex/Em = 522 nm/535 nm), with images captured immediately, with images captured immediately.

### 5.7 Preparation and Characterization of RAPA NPs

**(i) Synthesis and Standard Curve Establishment of RAPA NPs**. Initially, 2 mg of RAPA and 10 mg of PLGA were each dissolved in 0.5 mL of dichloromethane. Next, 0.1 mL of the RAPA solution was added to the PLGA dispersion and thoroughly mixed. Subsequently, 3 mL of 0.5% PVA solution (W/V) was added, and the mixture was processed using an ultrasonic disruptor at 20% power for 5 min. The obtained mixture was subsequently combined with 10 mL of ultrapure water and stirred on a magnetic stirrer at room temperature for 3 h to facilitate the evaporation of dichloromethane. Finally, the mixture was centrifuged at 22 000 g for 20 min to collect the pellet. This pellet was then resuspended in 4 mL of ultrapure water to produce the final product, PLGA‐RAPA (RAPA NPs), which was subsequently lyophilized for use in later experiments.

One milligram of RAPA powder was mixed with 1 mL of acetonitrile until fully dissolved. Acetonitrile was then used to perform a series of gradient dilutions to obtain concentrations of 1.113, 2.226, 4.453, 17.813, 35.625, and 71.25 µg/mL. High‐performance liquid chromatography (HPLC) was conducted for each concentration with a sample injection volume of 0.1 mL. The peak areas for each sample were measured, and a standard curve was constructed utilizing Origin 8.5 software.

**(ii) Characterization of RAPA NPs**. Initially, RAPA NPs powder was reconstituted in 1 mL of ultrapure water and then dispersed by applying ultrasonic treatment for 5 min. Subsequently, 0.1 mL of the dispersion was diluted 50‐fold with ultrapure water and applied to the surface of a carbon‐coated copper grid (300 mesh). After allowing it to air dry, the sample was placed on a sample holder and examined using transmission electron microscopy (TEM) to investigate its morphology.

To analyze the particle size, the RAPA NPs dispersion was diluted with ultrapure water in a 1:100 ratio, achieving a volume of 1.5 mL. This diluted solution was transferred to a quartz cuvette for particle size measurement. Additionally, for zeta potential measurements, the RAPA NPs dilution was placed in a specific electrode cup, following the instrument's instructions to position the electrode cup correctly, and measurements were taken using the potential module.

Dilute an appropriate amount of RAPA NPs with ultrapure water and store them at room temperature in a sealed container. And RAPA NPs were analyzed for particle size stability at predetermined time points: 0‐, 3‐, 6‐, 9‐, 12‐, 18‐, 24‐, and 30‐day, using a particle size analyzer, with stability assessed against initial measurements.

For drug release studies, the RAPA NPs dispersion was placed in a microdialysis device (10K MWCO) and incubated at room temperature with slow agitation at 40 rpm on a shaker. The final volume inside the dialysis cup was 0.5 mL, while the outer chamber was filled with 14.5 mL of PBS. At specified intervals 0, 2, 4, 6, 8, 10, 12, 24, 36, and 48 h. 0.5 mL of the external solution was collected and replaced with an equal volume of PBS. The samples obtained were detected through high‐performance liquid chromatography (HPLC), and the drug release efficiency of RAPA NPs was determined using the standard curve.

### 5.8 Synthesis and Characterization of TTM

RAW264.7 cells were plated into confocal dishes at a concentration of 5 × 10^4^ cells/mL. After allowing the cells to adhere, 50 µm AC_4_ManNAz and 100 ng/mL IFN‐γ were added to the culture, and the cells were incubated for 48 h. During this period, 25 µg/mL RAPA NPs were introduced into the dish, and the culture was continued at 37°C to construct **TTM**. Subsequent analyses were performed as follows:

- **TEM**. TEM was employed to determine the internal morphology of **TTM**. **TTM** cells were collected and then centrifuged at 1000 rpm for 5 min to discard the supernatant. The cell pellets were then resuspended in electron microscopy fixative and incubated in the dark for 30 min, after which they were transferred to 4°C for an additional hour to complete the fixation process. The samples subsequently underwent dehydration, infiltration, embedding, sectioning, and double staining with uranyl acetate and lead citrate. After drying the sections at room temperature, images were captured using TEM, and the phagocytic activity of the cells was analyzed.
- **HPLC**. To measure the RAPA content in **TTM** cells, the cells were collected and washed with PBS to eliminate any unbound RAPA NPs. Subsequently, a mild IP cell lysis buffer was added, and the mixture was placed in an ice bath for 2 min prior to sonication for 40 sec. The sample was then centrifuged at 14,000 rpm (or approximately 20,000 g) for 20 min to collect cell pellets. The pellets were resuspended in 0.3 mL of PBS containing 6% acetic acid, and the suspension was centrifuged at 800 g for 5 min. At last, the supernatant was analyzed for RAPA content using HPLC.
- **Western Blot (WB) and Real‐Time Fluorescence Quantitative PCR (qPCR)**. To establish that the TTM construction system retains its immunoregulatory functions, the study employed WB and qPCR to further assess the expression levels of PD‐L1 in **TTM**. RAW264.7, Mφ_PD‐L1_, Mφ_PD‐L1@N3_, and **TTM** cells were prepared according to the aforementioned induction steps. Cells were harvested on ice with a cell scraper and subsequently underwent cell lysis, ultrasonic disruption, and centrifugation to extract proteins and genes. β‐Actin served as the internal control. PD‐L1 expression levels were then determined using WB and qPCR techniques.

### 5.9 Visualization of Drug Uptake in Mφ_PD‐L1@N3_ Cells Using DiI NPs

First, CLSM was employed to observe the phagocytic activity of Mφ_PD‐L1@N3_ cells toward the drug. To visualize this process, fluorescent PLGA‐DiI nanoparticles (DiI NPs) were synthesized according to the preparation steps of RAPA NPs. Mφ_PD‐L1@N3_ cells were prepared according to the specified induction concentration. DiI NPs were then added, and the mixture was incubated at 37°C in a 5% CO_2_ environment for 8 h. After sufficient uptake, the cells were washed three times with PBS and fixed in 4% paraformaldehyde for 10 min. Following this, they were stained with DAPI in the dark for another 10 min before being examined under a microscope.

To further optimize **TTM**, the study investigated the optimal time point for DiI NPs uptake by Mφ_PD‐L1@N3_ cells. During the cultivation of Mφ_PD‐L1@N3_, 0.05 mg/mL of DiI NPs was added, and phagocytosis was terminated at different time points (0 h, 4 h, 8 h, 12 h, and 24 h). Following this, the cells were digested and collected, then resuspended in 0.3 mL of PBS before being transferred to flow cytometry tubes equipped with a filter. Finally, analysis was conducted using a Sony ID7000 spectral flow cytometer, configured with DiI parameters set to an excitation wavelength of 549 nm and an emission wavelength of 565 nm.

### 5.10 Evaluation of RAPA Release from TTMs Under Inflammatory Stimulation

To mimic the inflammatory microenvironment, **TTM** cells were stimulated with 2 µg/mL LPS. At defined time points (0, 2, 4, 6, 8, 10, 12, 24, and 36 h), cells were harvested, washed twice with PBS, and centrifuged at 1000 rpm for 5 min to remove supernatants. The pellets were resuspended in 0.3 mL PBS, and 20 µL acetic acid was added to lyse the cells. The suspensions were mixed thoroughly by pipetting and centrifuged for 30 s at high speed. Supernatants were analyzed by HPLC under isocratic conditions to determine intracellular rapamycin content, quantified using a standard calibration curve. Rapamycin release rate was calculated as: Release rate = (Total encapsulated drug‐Intracellular drug at time t)/Total encapsulated drug × 100%.

### 5.11 Assessment of TTM Viability Using CCK‐8 Assay

RAW264.7 cells were adjusted to a concentration of 2 × 10^4^ cells/mL prior to being seeded into a 96‐well plate. Subsequently, these cells were divided into the following groups: control (RAW264.7), Mφ_PD‐L1_, Mφ_N3_, and **TTM**. Each group was subjected to different induction treatments. Afterward, 10 µL of CCK‐8 solution was introduced into each well. Then, the cells were cultured at 37°C for 1.5 h, after which the optical density (OD) at 450 nm was assessed via a microplate reader. Determine the cell viability using the formula provided in the manufacturer's instructions:

$$\begin{array}{c} \mathrm{Cell}\;\mathrm{viability}\;\left(\%\right)=\left(OD_{\mathrm{control}}-OD_{\mathrm{blank}}\right)/\left(OD_{\mathrm{sample}}-OD_{\mathrm{blank}}\right)\\ \times 100\% \end{array}$$

### 5.12 Detection of TTM Migration Using the Transwell Assay

The migration capacity of **TTM** cells was evaluated through a Transwell assay. The **TTM** cells were first collected and then resuspended in an appropriate medium. After resuspension, the cell suspension was transferred to the upper chamber of the Transwell apparatus. And the lower chamber contains complete culture medium enriched with 10 ng/mL of the chemokine MCP‐1. After incubation for a specified period, cells that had migrated via the membrane into the lower chamber were fixed, stained, and then detected under a microscope. To evaluate the migration ability of **TTM** cells, the number of migrated cells was quantified.

### 5.13 Evaluation of PD‐L1 Stability

To investigate the long‐term dynamics of PD‐L1 expression, TTMs were maintained in culture after the removal of induction stimuli. Cells were harvested at defined intervals (0, 48, 96, and 144 h) for analysis. PD‐L1 surface retention was quantified via immunofluorescence staining using a standard protocol (primary anti‐PD‐L1 antibody followed by FITC‐conjugated secondary antibody) and analyzed using confocal microscopy (E_x_/E_m_ = 495/520 nm).

### 5.14 Analysis of Cell Viability and Toxicity Using Calcein‐AM/PI Staining

To evaluate the toxicity effects of engineered induction on cells, a fluorescence microscopy analysis with Calcein‐AM and PI dual staining was conducted. The experiment was divided into four groups: control (RAW264.7), Mφ_PD‐L1_, Mφ_N3_, and **TTM**. Initially, RAW264.7 cells were seeded in a 96‐well plate and then subjected to the respective induction for each group. Following treatment, a working solution of Calcein‐AM and PI was prepared based on the manufacturer's guidelines and then added to each well at a volume of 0.1 mL. The cells were incubated at 37°C in the dark for 30 min. After three washes with PBS, images were captured with a fluorescence microscope (Calcein‐AM Ex/Em = 494 nm/517 nm, PI Ex/Em = 535 nm/617 nm).

### 5.15 Measurement of Cellular ROS Levels Using DCFH‐DA

In accordance with the instructions included with the reactive oxygen species (ROS) detection kit, the DCFH‐DA was diluted in serum‐free medium to prepare a 10 µm probe working solution. These cells, seeded in a 96‐well plate, were removed from the incubator, and the medium was removed. Next, the working solution of DCFH‐DA was added to the wells and thoroughly mixed. Following a 20‐min incubation at 37°C, the cells were washed with PBS to eliminate any unbound probe. Afterward, the DAPI solution was added to each well to stain in the dark for 10 min. Finally, fluorescence microscopy and a microplate reader were used to detect fluorescence.

### 5.16 Detection of M1 Macrophages by Flow Cytometry

**TTM** cells, 1 × 10^8^, were seeded into a six‐well plate and treated with 1 µg/mL LPS for 24 h. Subsequently, the cells were collected into a centrifuge tube, washed with PBS, and centrifuged at 250 g to remove the supernatant. The cell pellet was then resuspended in 50 µL of PBS, and 1 µL of F4/80 and CD86 antibodies were added. The mixture was incubated in the dark for 25 min. Afterward, the cells were washed three times with 2 mL of PBS, vortexed, and then fixed by adding 500 µL of fixation solution, followed by incubation in the dark at 4°C for 30 min. Finally, the cells were washed three times with PBS and transferred to a flow cytometry tube for analysis using a SONY ID7000 spectral flow cytometer.

### 5.17 Quantification of Inflammatory Cytokines in TTM Cells by ELISA

RAW264.7 and **TTM** cells were cultured in a six‐well plate. Subsequently, 0.2 mL of IP lysis buffer was added to each well, and the cells were incubated on ice for 3 min. Cell clumps were collected using a cell scraper. The cell suspension was subsequently treated with ultrasonic disruption on ice for 40 sec, followed by centrifugation at 14 000 rpm for 20 min at 4°C to obtain the supernatant. Then, the total protein concentration in the supernatant was detected via a BCA protein assay kit. Protein concentrations were adjusted to 20 µg/mL with PBS to ensure consistency across samples. IL‐1, IL‐2, IL‐6, and TNF‐α ELISA kits were allowed to equilibrate at room temperature for 20 min. Following the manufacturer's guidelines, the procedure involved adding 50 µL of standards and samples to the wells. Next, 100 µL of horseradish peroxidase‐conjugated antibody was added and then incubated in the dark at 37°C for 1 h. After washing, substrate reaction and stopping solutions were applied. Absorbance at 450 nm was recorded by a microplate reader. The cytokine levels in **TTM** cells were assessed by comparing the sample absorbance to a standard curve created from the standards.

### 5.18 Construction of a Skin Transplant Rejection Mice Model

**Animals**: BALB/c and C57BL/6J mice utilized in this study were obtained from Beijing Vital River Laboratory Animal Technology Co., Ltd. The male mice used in the study were aged 6 to 8 weeks and had an average weight of about 20 g. Upon arrival, the mice were quarantined and kept in a SPF facility at Tongji Medical College, Huazhong University of Science and Technology. All procedures involving feeding, experimentation, and euthanasia adhered to the standards set by the Animal Ethics Committee of Huazhong University of Science and Technology. Ethical approval was obtained for this study (Approval No. 3636). In this study, no animals died unexpectedly during the course of these experiments, and no data points were discarded or excluded from the analysis. The sample sizes reported in the original manuscript reflected the initial experimental design, not the exclusion of outliers. All animals reached the pre‐defined endpoints, and all collected data are reported.

**(i) Allogeneic Dorsal Skin Grafting**. BALB/c mice were anesthetized and positioned dorsal side up on a surgical board. The dorsal fur was shaved with a razor, followed by the application of hair removal cream on gauze to remove any residual fine hairs. The skin was then disinfected with 10% povidone–iodine using a cotton ball. Pre‐sterilized surgical instruments were employed for the procedure. The buttock skin was lifted with ophthalmic forceps, and skin scissors were used to make a longitudinal incision from the buttocks to the neck. Following this, the mouse was euthanized. An 8 cm × 8 cm sterile gauze was spread on a 10 cm dish and moistened with physiological saline to enhance adhesion. The excised mouse skin was placed on the gauze. Using ophthalmic forceps and working on ice, the attached connective tissue, fat, and blood vessels were carefully dissected away from the skin. Throughout this process, pre‐cooled physiological saline was continuously applied to maintain moisture in the skin graft. The skin was then trimmed into sections of 0.8 cm × 1.2 cm for subsequent use.

C57BL/6J mice were deeply anesthetized. The fur on the dorsal region was shaved, and depilatory cream was applied to clean the skin. The area was then disinfected with povidone‐iodine. A corresponding skin graft was harvested from the central region of the dorsal skin. Using sterile 6‐0 absorbable sutures, the donor skin was quickly sutured onto the recipient site. Finally, a layer of Vaseline gauze was covered over the graft site to maintain skin moisture, and a bandage was used to cover the mouse's forelimb to protect the transplanted area.

**(ii) Identification of Skin Transplant Rejection**. Post‐operatively, the mice were observed daily for changes in skin color and shrinkage of the grafted area. On post‐operative days 3, 5, and 7, the grafted skin was collected for histological analysis. The collected specimens were stained with hematoxylin and eosin (H&E) and detected using a high‐resolution pathological slide scanner to assess inflammatory cell infiltration. The degree of rejection was then determined according to the Banff classification system. Herein, skin from donor C57BL/6J mice was transplanted onto recipient C57BL/6J mice to generate syngeneic transplant mice (isograft mice), which were used as the control group for assessing the pathological scoring of the rejection model.

### 5.19 In Vivo Imaging and Biodistribution Analysis in Allograft Mice

- **In Vivo Imaging of TTM Labeling at Various Time Points. TTM** cells were seeded in 10 cm dishes, and cell aggregates were collected by aspirating the bottom of the dish with serum‐free medium. Following centrifugation at 1000 rpm for 5 min, the cells were resuspended in pre‐warmed PBS at 37°C. On the fourth day post‐transplantation, 5 × 10^6^ **TTM** cells were administered through the tail vein of the mice. Subsequently, 24 h after circulation of **TTM** cells, mice were administered a tail vein injection of 0.5 mg/kg DBCO‐Cy5. Mice were anesthetized at the following time points: 0, 4, 6, 8, 10, 12, 24, and 48 h. They were then placed in an imaging system for small animals. Images were captured with an exposure time of 3 s and a camera‐to‐panel distance of C field of view (Cy5 Ex/Em = 620 nm/670 nm).
- **Comparison of TTM Migration across Different Rejection Grades**. On the second, fourth, and sixth postoperative days, 5 × 10^6^ **TTM** were intravenously administered into the tail veins of the allograft mice. 24 h post‐cell migration, on the third, fifth, and seventh postoperative days, 0.5 mg/kg DBCO‐Cy5 was administered. Following a 6‐h dark incubation period, the mice were anesthetized, and the graft site was observed using the IVIS imaging system.
- **Analysis of Biodistribution of PBS, RAW264.7 TTM and Azido‐Labeled ESCs, DC2.4, or MSCs**. On the fourth day post‐skin transplantation in allograft mice, 5 × 10^6^ cells were injected into the tail veins. Mice in the control group were given a matching volume of PBS. 24 h later, on the fifth postoperative day, 0.5 mg/kg DBCO‐Cy5 was administered to all groups. After a 6‐h reaction period, images were captured using an in vivo imaging system. At last, the mice were euthanized, and the heart, liver, spleen, lungs, kidneys, and allograft skin were harvested, rinsed with PBS, and subjected to fluorescence imaging against a black background.

### 5.20 Colocalization Analysis of CD68 and Cy5 in Allograft Skin Tissue

Allograft mice were established and subsequently divided into three groups: RAW264.7, **TTM**, and DBCO‐Cy5, with five mice in each group. On the fourth postoperative day, each mouse received an intravenous infusion of an equal number of cells. However, the DBCO‐Cy5 group received only PBS. On the fifth postoperative day, all groups were administered 0.5 mg/kg DBCO‐Cy5 via tail vein injection. After a 6‐h reaction period, the mice were euthanized, and samples of the transplanted skin were collected. The skin tissues were rinsed with PBS, transferred to cryovials, and quickly frozen using liquid nitrogen. Then, these tissues were embedded in OCT, frozen overnight at −80°C, and subjected to sectioning, mounting, blocking, primary antibody incubation, washing, secondary antibody labeling, washing, nuclear counterstaining, and final mounting. Finally, the distribution of **TTM** in the transplanted skin was observed using an inverted laser confocal microscope.

### 5.21 Western Blot and Quantitative PCR Analysis

On the third and seventh days post‐skin transplantation, mice were euthanized, and transplanted skin tissues were collected and rapidly frozen using liquid nitrogen. Proteins and RNA were then extracted from these skin tissues. WB and qPCR were used to analyze the expression levels of CSF‐1, MCP‐1, and CD68. Details of primer design sequences are provided in Table S2.

Additionally, to further investigate the immunosuppressive mechanisms of **TTM** cells, skin tissues from the treatment group were collected, treated with liquid nitrogen, and subsequently processed for gene extraction. Finally, quantitative PCR (qPCR)was conducted to evaluate the expression of C3a, C5a, C3ar, C5ar, CCL5, and CCR5 in the grafts. Details of primer design sequences are provided in Table S3.

Furthermore, the study utilized WB to further examine the protein expression levels of p‐PI3K, PI3K, p‐Akt, Akt, p‐mTOR, mTOR, and FOXP3 in the transplanted grafts on day 9 post‐transplantation. GAPDH was employed as the internal control protein. The study quantified the grayscale values of the target proteins in relation to the internal control.

### 5.22 Evaluation of the TTM Biosafety

**(i) Hemolysis Toxicity**. Normal C57BL/6J mice were selected for blood collection via ocular puncture to isolate red blood cells. These red blood cells were then resuspended in saline at a 10‐fold volume, centrifuged at 1000 rpm for 5 min, and the resulting supernatant was removed. This wash step was repeated three times. The red blood cells were then diluted with saline to prepare a 4% suspension. This suspension was then separated into five groups, with each group containing 1 mL: ① PBS group: served as the negative control, with 0.2 mL PBS added and mixed; ② RAW264.7 group: 1 × 10^7^ RAW264.7 cells were combined with the red blood cell suspension; ③ Mφ_PD‐L1@N3_ group: 1 × 10^7^ Mφ_PD‐L1@N3_ cells were added to the red blood cell suspension; ④ **TTM** group: 1 × 10^7^ **TTM** cells were mixed with the red blood cell suspension; ⑤ Triton X‐100 group: served as the positive control, with 2% Triton X‐100 incorporated into the red blood cell suspension. And the mixtures were incubated at 37°C for 1 h. After incubation, the suspensions were centrifuged at 3000 rpm for 10 min. The images of the supernatants were then captured. Additionally, 100 µL of each supernatant was transferred to a 96‐well plate with three replicates per group. Absorbance at 540 nm was recorded with a microplate reader to determine the hemolysis rate [97].

**(ii) ELISA Measurement of Serum Inflammatory Cytokine in Vivo**. Fifteen C57BL/6J mice were randomly assigned to 3 groups, each consisting of five mice. Each group received injections via the tail vein every 48 h with either 0.2 mL PBS, 5 × 10^6^ RAW264.7 cells, or 5 × 10^6^ Mφ_PD‐L1@N3_ cells, for a total of three injections. On the seventh day, blood was collected from each group and centrifuged at 3000 rpm for 15 min. Serum obtained was divided into aliquots and stored at −80°C for future analysis.

ELISA kits for IL‐1, IL‐2, IL‐6, and TNF‐α were removed from the refrigerator and equilibrated at room temperature for 30 min. And the serum samples were diluted three‐fold using the sample diluent provided. The diluted samples and standards were added to antibody‐coated wells of the ELISA plate. The plate was cultured at 37°C for 1 h with horseradish peroxidase‐conjugated antibodies, followed by washing, substrate reaction, and reaction termination. Absorbance was measured within 15 min using a microplate reader at 450 nm.

**(iii) Immunogenicity Assessment**. C57BL/6J mice were randomly assigned to 3 groups, receiving injections of either 0.2 mL PBS, 5 × 10^6^ RAW264.7 cells, or 5 × 10^6^ **TTM** cells. Injections were administered every 48 h. The study included groups with a total of 3, 6, or 9 dose‐injections. Blood samples were collected via retro‐orbital puncture on days 6, 12, and 18, followed by serum separation through centrifugation. Subsequent ELISA assays were performed following the instructions provided with IgG and IgM kits, with serum samples being diluted as specified.

**(iv) Biochemical and Hematological Analysis**. C57BL/6J mice were divided into 3 groups: PBS, RAW264.7, and **TTM**. Mice in each group received continuous intravenous injections as previously described. Blood was obtained from the mice, and the serum was subsequently isolated on days 6, 12, and 18. Next, serum biochemical parameters were assessed using an automated biochemical analyzer according to the instructions provided with the relevant kits. The parameters measured included alanine aminotransferase (ALT), blood urea nitrogen (BUN), creatinine (CREA), aspartate aminotransferase (AST), albumin (ALB), total bilirubin (TBIL), uric acid (UA), and total cholesterol (CHO). Additionally, a complete blood count (CBC) was performed on the treated mice. Whole blood samples were drawn into anticoagulant tubes and subsequently analyzed with a hemocytometer. The following hematological parameters were measured: white blood cells (WBC), red blood cells (RBC), platelets (PLT), lymphocyte percentage (Lymph%), neutrophil percentage (Gran%), monocyte percentage (Mon%), hemoglobin (HBG), lymphocyte count (Lymph#), neutrophil count (Gran#), and mean corpuscular volume (MCV).

**(v) Assessment of Major Organ Pathological Damage**. Mice that had received 3, 6, and 9 doses of cell intravenous injections were euthanized via cervical dislocation. Subsequently, the heart, liver, spleen, lungs, and kidneys were extracted and rinsed with physiological saline. These organs were fixed in 4% paraformaldehyde solution and subsequently subjected to H&E staining. Images of the stained tissues were captured using a high‐resolution pathological slide scanner for further analysis.

### 5.23 In Vivo Evaluation of TTM Treatment on Allograft Mice

**Treatment Protocol**. Allograft mice were randomly divided into five groups: PBS, RAW264.7, Mφ_PD‐L1_, RAPA NPs, and **TTM**, with 10 mice per group. On the third day post‐transplantation, each group received intravenous treatment. Specifically, the RAPA NPs group was administered 2 mg/kg RAPA per mouse, the Mφ_PD‐L1_ group was given 5 × 10^6^ cells per mouse, and the **TTM** group also received 5 × 10^6^ cells per mouse along with a RAPA dose of 2 mg/kg, consistent with the RAPA NPs group. All treatments were administered at a volume of 0.2 mL on postoperative days 3, 5, and 7. On day 9, the mice were euthanized, and grafts, serum, and other samples were collected for subsequent analysis:

- **Assessing Graft Survival Rates and Body Weight**. During the cell reinfusion treatment, the physiological condition of the grafts was monitored to determine their survival status. Grafts were considered to be dead if they exhibited signs of shrinkage, discoloration to dark or black, necrotic areas with no apparent blood supply, and a hard, non‐rebounding texture upon palpation. Conversely, grafts were recorded as alive if the graft tissue appeared uniformly normal in color, was moist and resilient, and showed no signs of bleeding or inadequate healing at the sutured edges. Furthermore, the weights of the mice in the aforementioned groups were measured using a small animal electronic scale.
- **Immunohistochemistry**. On day 9 post‐surgery, following the end of treatment, mice were euthanized. Using ophthalmic forceps and scissors, the transplanted skin tissue was carefully excised, and any suture remnants or foreign materials were removed. The tissue was then rinsed in PBS and fixed in the dark with 4% paraformaldehyde. Subsequently, the tissue underwent pathological immunohistochemical staining for H&E, CD3, Ki67, and Granzyme B. Finally, a high‐resolution pathological slide scanner was employed to examine the pathological damage in the transplanted skin sections.
- **ELISA Analysis of Serum Inflammatory and Anti‐Inflammatory Cytokine Levels**. On day 9 post‐surgery, the mice were euthanized, and the eyeballs were removed using sterile ophthalmic forceps to collect blood. After standing at room temperature for 1.5 h, the blood was centrifuged at 3000 rpm for 15 min to isolate the serum. The serum was then diluted threefold with sample diluent and analyzed for IL‐2, IL‐4, IL‐6, TNF‐α, and IL‐10 following the kit's instructions. Absorbance readings at 450 nm were obtained for subsequent statistical analysis.

### 5.24 RNA Sequence

On day 9 post‐transplantation, we collected the mouse allografts for the following RNA sequence analysis: RNA extraction was performed using TRIzol reagent (Fisher Scientific, 15596018), followed by purification with the NucleoSpin RNA XS kit (Takara Bio, U0902A) in accordance with the manufacturer's protocol. RNA quantification was performed with a Qubit 2.0 fluorometer (Thermo Fisher Scientific). The library was diluted to a concentration of 1.5 ng/µL, and its insert size was assessed on the Agilent Bioanalyzer 2100, confirming an effective concentration above 2 nM.

Index‐coded samples underwent clustering on a cBot Cluster Generation System with the TruSeq PE Cluster Kit v3‐cBot‐HS (Illumina) following the provided protocols. After cluster generation, sequencing was performed on the Illumina NovaSeq platform, yielding 150 bp paired‐end reads. Reference genome and gene model annotation files were acquired from the genome website, and an index for the reference genome was created using Hisat2 v2.0.5. The paired‐end clean reads were aligned to this reference genome using the same software. Hisat2 was selected for its ability to generate a splice junction database from the gene model annotation, enhancing mapping accuracy over non‐splice mapping tools.

For gene expression quantification, featureCounts v1.5.0‐p3 was employed to count the number of mapped reads, and the FPKM (Fragments Per Kilobase of transcript per Million mapped reads) values for each gene were calculated based on gene length and mapped read counts. The DESeq2 package was utilized to compute expression levels for normalized counts, including only genes with counts greater than one, for further analysis. Heatmaps of these genes were created using the R package, with differentially expressed genes (DEGs) defined as those with an average log‐transformed difference greater than 1 and a p‐adj value below 0.05. GO enrichment analyses were conducted using the clusterProfiler (version 3.8.1) software, which also corrected for gene length biases.

### 5.25 In Vitro Analysis of Immunosuppressive T Cell Activity

- **Extraction and Isolation of Mouse Spleen CD4^+^ T Cells**: A healthy 6‐week‐old male C57BL/6J mouse was euthanized by cervical dislocation and immediately immersed in 75% ethanol for 15 min. The sterile workbench was then subjected to UV irradiation for 15 min, and the bench surface was wiped with 75% ethanol. A clean, sterile gauze was placed on the bench, and the mouse was positioned with its abdomen facing up on the gauze. An incision was made along the abdomen using ophthalmic scissors to extract the spleen. The spleen was then placed onto a 70 µm filter mesh pre‐wetted with 2% FBS‐PBS in a 50 mL non‐enzymatic centrifuge tube. Using a rubber stopper from a 2 mL syringe, the spleen was mashed until the deep red tissue clumps were dissolved, adding PBS as needed. The resulting suspension was transferred into a 15 mL conical centrifuge tube. It was then centrifuged at 300 g for 10 min to prepare for subsequent applications.
  The mouse T cell isolation kit was equilibrated at room temperature for 15 min. FcR blocker, Isaolation Cocktail, and RapidSpheres were vortexed for 30 sec. The cell clumps were resuspended in 1 mL of 2% FBS‐PBS, and the cell count was measured using a cell counter. The cell density was modified to 1 × 10^8^ cells/mL. Subsequently, the suspension was transferred to a 5 mL polystyrene round‐bottom tube. An FcR blocker was added and mixed thoroughly, followed by the addition of Isolation Cocktail. The mixture was then incubated at room temperature for 10 min. RapidSpheres were added, and the reaction continued for 2.5 min. Once the volume was adjusted to 2.5 mL, the tube was placed in a magnetic separator. After 2.5 min, the supernatant was rapidly collected, yielding a purified CD4^+^ T lymphocyte suspension.
- **Identification of CD4^+^ T Cells**. T cells collected by the aforementioned method were washed with PBS, and the cell density was adjusted to 1 × 10^6^ cells/mL, then aliquoted into flow cytometry tubes. Following the manufacturer's instructions, the cells were stained with BV510 viability dye (FVS) for 20 min in the dark. Subsequently, PBS was added to terminate the staining reaction. The cells were centrifuged at 250 × g for 10 min, and the supernatant was discarded. Fluorescently labeled anti‐CD45, anti‐CD3, and anti‐CD4 antibodies were added and mixed, followed by incubation at 4°C in the dark for 30 min. To remove unbound antibodies, 1 mL of PBS was added to each tube to wash the cells. The washing process included centrifugation at 250 × g for 10 min, and the supernatant was discarded. This step was repeated twice. Finally, the cells were resuspended in 300 µL PBS and analyzed using flow cytometry.
- **Detection of TTM Mixed Incubation Ratios for CD4^+^ T Cells**. The study involved mixing ultrapure anti‐CD3 and anti‐CD28 antibodies according to the manufacturer's instructions and precoating a 96‐well plate. Subsequently, CD4^+^ T lymphocyte suspension was resuspended in 1640 medium containing 10% FBS and seeded into the 96‐well plate. And then the plate was incubated at 37°C with 5% CO_2_ for 4 h. Following this, the cells were co‐cultured in a cell culture box for 72 h according to the ratios of **TTM**: CD4^+^T cell (0:1, 1:4, 1:8, 1:16, and 1:32). After incubation, the plate was placed in a centrifuge and centrifuged at 1000 rpm for 5 min. The supernatant was then discarded, and CCK‐8 solution was added to each well. Incubate at 37°C for 1.5 h. Finally, absorbance values were measured at 450 nm using a microplate reader.
- **Evaluation of TTM Inhibition on CD4^+^ T Cells Using CFSE**. First, 100 µg of CFSE powder was prepared as a 5 mM stock solution. The stock was then diluted 1000‐fold with culture medium to obtain a 5 µm CFSE working solution. CD4^+^ T lymphocytes were extracted as described earlier. After centrifugation at 1000 rpm for 3 min, the cell was then adjusted to 5 × 10^6^ cells/mL. CFSE working solution was added, and staining was conducted at 37°C for 20 min, followed by termination of the reaction with five‐fold volumes of culture medium. CFSE‐labeled CD4^+^ T cells were then seeded into a 96‐well plate and co‐incubated with **TTM** cells at a 1:4 ratio. For comparison, RAW264.7 and Mφ_PD‐L1_ cells were used to mix with T cells at the same ratio, and a Control group with equal volumes of culture medium was also prepared. The plate was cultured at 37°C for 72 h, and the T lymphocyte suspension was collected. Finally, flow cytometry was employed with a CFSE fluorescence selection module to quantify the proliferation rates of T lymphocytes in each group (Ex/Em = 492 nm/517 nm).
- **ELISA Detection of Inflammatory Levels in CD4^+^ T Cells Supernatants Induced by TTM**. As previously described, a 96‐well plate was precoated with mixed antibodies. RAW264.7, Mφ_PD‐L1_, and **TTM** cells were each mixed with CD4^+^ T lymphocytes at a 1:4 ratio in the wells of the plate. The control group consisted of CD4^+^ T lymphocytes combined with an equal volume of culture medium. After incubating the cells at 37°C with 5% CO_2_ for 72 h, the supernatants from the wells were collected using a pipette. At last, the concentrations of IL‐2, IL‐4, IFN‐γ, and IL‐10 in the supernatants were assessed through ELISA assays.

### 5.26 Evaluation of TTM's Modulation of T Lymphocyte Activity In Vivo

Allograft mice from the PBS, RAW264.7, Mφ_PD‐L1_, RAPA NPs, and **TTM** groups were subjected to corresponding intravenous injection treatments on postoperative days 3, 5, and 7. On day 9 post‐surgery, the mice were euthanized by cervical dislocation for the following assessments:

- **Extraction of Mouse Spleen and Lymph Node Cells**. ① Animal Dissection: On the dissection board, the mice were pinned with their limbs fixed, and the abdominal cavity was opened with ophthalmic scissors. The intestines were gently moved aside with cotton swabs to expose the spleen, which was then removed using ophthalmic forceps. Next, under a stereomicroscope, the bilateral axillary regions were opened, and fat and muscle tissues were separated to retrieve the light yellow, pear‐shaped lymph nodes. ② Tissue Processing: The PBS buffer supplemented with 20% FBS was added to each well of a six‐well plate. The separated spleen and lymph node tissues were rinsed once with PBS to eliminate any blood and debris. And the tissues were then thoroughly immersed in the plate to maintain cell viability. ③ Cell Separation: A 70 µm sterile filter was placed over a 50 mL conical centrifuge tube, and the filter was wetted with PBS containing 20% FBS. Tissue was then minced with a syringe plunger, and PBS was added as needed to keep the tissue moist during the mechanical dispersion, ensuring thorough passage through the filter. The final suspension was adjusted to 5 mL.
- **Preparation of Single‐Cell Suspension from Allograft Skin Tissue**.①Tissue Processing. Allograft skin tissue was collected and washed with physiological saline. The tissue was then cut into fragments of ≤1 mm^3^ using sterile scissors and transferred into a 2 mL grinding tube.②Cell Dissociation. To each tube, 1 mL of cell dissociation solution was added and incubated at 37°C in a shaking incubator (80 rpm) for 1 h. After incubation, the tube was vortexed vigorously for 40 s, and the resulting cell suspension was transferred to a 15 mL centrifuge tube. To terminate the dissociation, 10 mL of PBS was added. The suspension was then filtered through a 70 µm sterile cell strainer to remove undigested tissue debris.③Cell Washing and Collection: The suspension was centrifuged at 250 × g for 10 min, and the supernatant was discarded. The pellet was resuspended in 10 mL of PBS and washed twice. The cell pellet was then collected and reserved for further processing.④Cell Seeding. The cells were counted using an automatic cell counter, then resuspended to a density of 1 × 10^6^ cells per 100 µL RPMI‐1640 medium. The cells were seeded into a 24‐well plate, with 1 mL of RPMI‐1640 medium added to each well. After the addition of brefeldin A (BFA, 5 µg/mL), the mixture was gently vortexed and incubated in a 37°C, 5% CO_2_ incubator for 4 h.⑤Cell Collection and Staining Preparation. After the incubation, the cells were collected by gentle pipetting, transferred to 5 mL flow cytometry tubes, and centrifuged at 250 × g for 10 min. The supernatant was discarded, and the cells were washed once with PBS. The cell pellet was collected for subsequent staining.
- **Surface Staining**. According to the instructions, cells derived from spleen, lymph nodes, and skin tissues were subjected to FCR blocking for 15 min, followed by PBS addition to terminate the blocking process. Next, antibody staining solutions for FVS, CD45, CD3, CD4, CD8, and CD25 were prepared and sequentially added to the cell suspension. The cells were stained in the dark for 30 min. The samples were then centrifuged at 500 × g for 7 min at 4°C, and the supernatant was discarded. Subsequently, 1 mL of Fix reagent from the membrane disruption kit was added to the cells, and the samples were fixed at room temperature for 1 h.
- **Intracellular and Nuclear Staining**. Next, 2 mL of Perm reagent was added to each flow cytometry tube, and the mixture was thoroughly mixed. The samples were centrifuged at 500 × g for 5 min, and the supernatant was discarded. Then, the cells were resuspended in a working solution of diluted Foxp3, IL‐10, IFN‐γ, and Granzyme B antibodies in Perm liquid. The cells were mixed well and stained for 40 min. After the staining protocol, the cells were washed with Perm liquid and resuspended in 0.2 mL PBS. The resulting suspension was filtered through a 300‐mesh cell filter and then analyzed by flow cytometry to detect and sort the CD8^+^ T cell population, Treg cells, CD8^+^IFN‐γ^+^ T cells, CD8^+^GzmB^+^ T cells, and Foxp3^+^IL‐10^+^ Treg cells. To ensure consistency of the assays, the laser parameters of the flow cytometer were set as follows: 405 nm at 100 mW, 488 nm at 150 mW, 561 nm at 100 mW, and 637 nm at 140 mW. And each group underwent flow cytometric sorting under the same voltage conditions using cells from the same tissue source, with each group consisting of 5 biological replicates.

### 5.27 Immunofluorescence

Following the treatment period, allograft mice from the PBS, RAW264.7, Mφ_PD‐L1_, RAPA NPs, and **TTM** groups were euthanized by cervical dislocation on the ninth day post‐transplantation. The allograft skin tissues were excised and fixed in 4% paraformaldehyde for thorough fixation. Following this, immunofluorescence staining was observed on the transplanted tissue sections to evaluate the levels of CD4, CD8, IFN‐γ, and Foxp3.

### 5.28 Statistical Analysis

Data analysis and graphical preparation were performed utilizing GraphPad Prism software. The summarized data are expressed as mean ± standard deviation (SD). Blinding or randomization methods were implemented. In comparing the two sets of data, we employed Student's *t*‐test. For comparisons involving three or more groups, we utilized one‐way analysis of variance (ANOVA). Statistically, a *p*‐value of < 0.05 is considered indicative of a significant difference.

## Author Contributions

W.Y.H. and S.Y. contributed equally to this work. X.M.X. and G.T. conceived this work. W.Y.H. designed, prepared, and characterized the engineered macrophages. W.Y.H. and S.Y. carried out the in vitro, ex vivo, and in vivo experiments and wrote the manuscript. X.Y.J., Z.J.M., L.J, and W.W.Y. contributed technical support. X.Y., W.W.Q., Z.W.Q., and J.Q.F. discussed the results. W.J. and Y.Y.L. edited the manuscript. Z.L and X.M.X. supervised the project.

## Conflicts of Interest

The authors declare no conflicts of interest.

## Supporting information




**Supporting File**: advs74232‐sup‐0001‐SuppMat.docx.

**Supplemental Movie 1**: advs74232‐sup‐0002‐MovieS1.mov.

**Supplemental Movie 2**: advs74232‐sup‐0003‐MovieS2.mov.

**Supplemental Movie 3**: advs74232‐sup‐0004‐MovieS3.mov.

**Supplemental Movie 4**: advs74232‐sup‐0005‐MovieS4.mov.

**Supplemental Movie 5**: advs74232‐sup‐0006‐MovieS5.mov.

**Supplemental Movie 6**: advs74232‐sup‐0007‐MovieS6.mov.

## Data Availability

Further information and requests for resources and reagents should be directed to and will be fulfilled by the lead contact, Prof. M. Xie (xiemx@hust.edu.cn). Sequencing data applied in this study can be accessed in the public SRA database under the number PRJNA1255121.
