# Supplementary material for: Trackable Tolerogenic Macrophages Integrate PD‐L1 and Rapamycin Signaling to Suppress Alloimmune Responses in Transplantation
Source: Adv Sci (Weinh). 2026 Feb 8;13(21):e20420. doi: 10.1002/advs.202520420 (PMC13073329; doi:10.1002/advs.202520420)
Supplement: Supplementary file 1 — Supporting File: advs74232‐sup‐0001‐SuppMat.docx. [file ADVS-13-e20420-s001.docx]

Supplemental Information for

**Trackable Tolerogenic Macrophages Integrate PD-L1 and Rapamycin Signaling to Suppress Alloimmune Responses in Transplantation**

Yi-Hui Wang *et al.*

*Corresponding author. Email: [gaotang@hust.edu.cn](mailto:gaotang@hust.edu.cn%20(T) ; xiemx@hust.edu.cn.

**This word file includes:**

Figures. S1 to S46

Tables S1 to S3

Legends for movies S1 to S2

**Other Supplemental Information for this manuscript include the following:**

Movies S1 to S2

Supplemental Figures


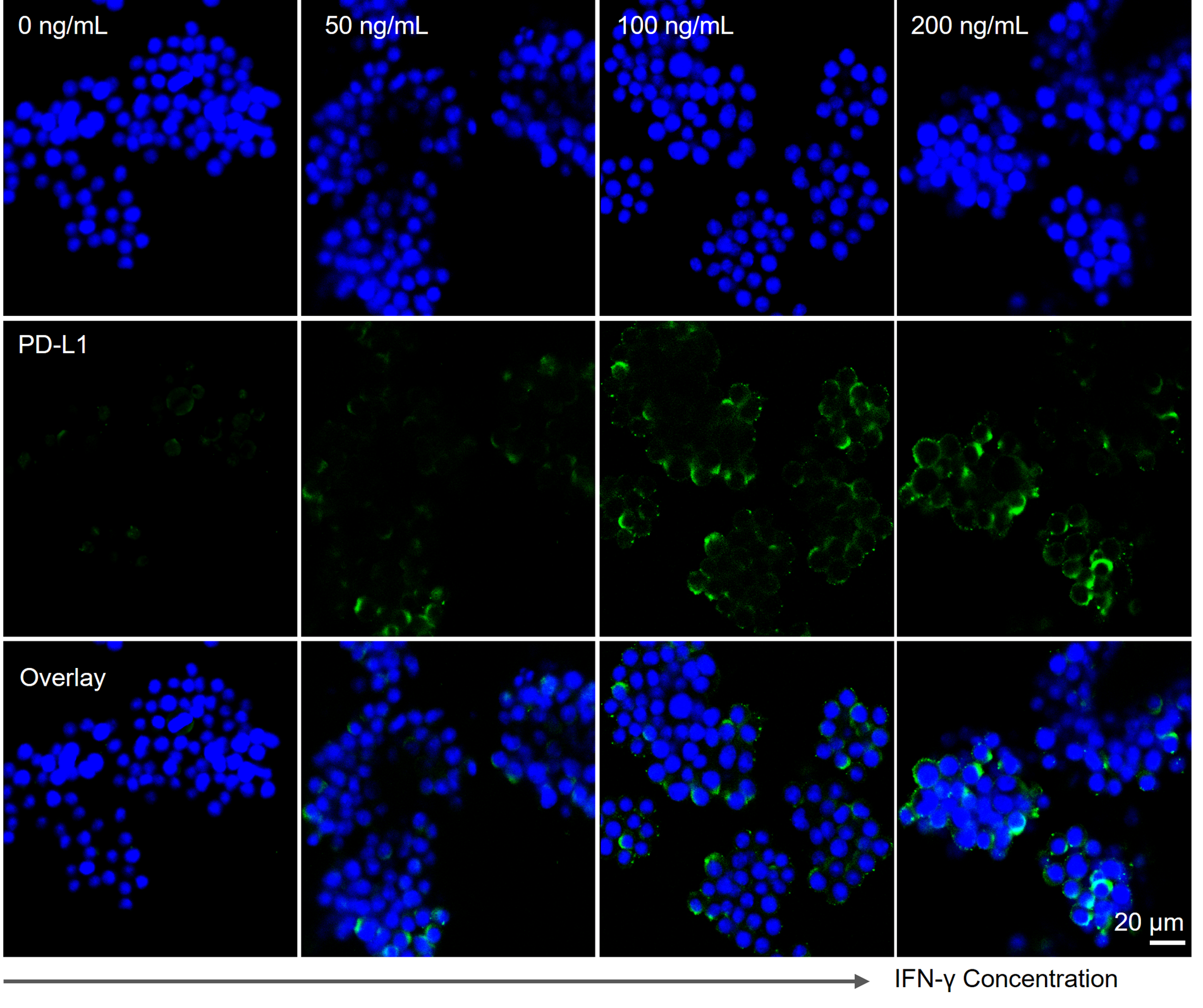


Figure S1 Laser confocal microscopy was utilized to examine the overexpression of PD-L1 on the surface of RAW264.7 cells induced by IFN-γ concentrations of 0, 50, 100, and 200 ng/mL, with a scale bar of 20 μm.


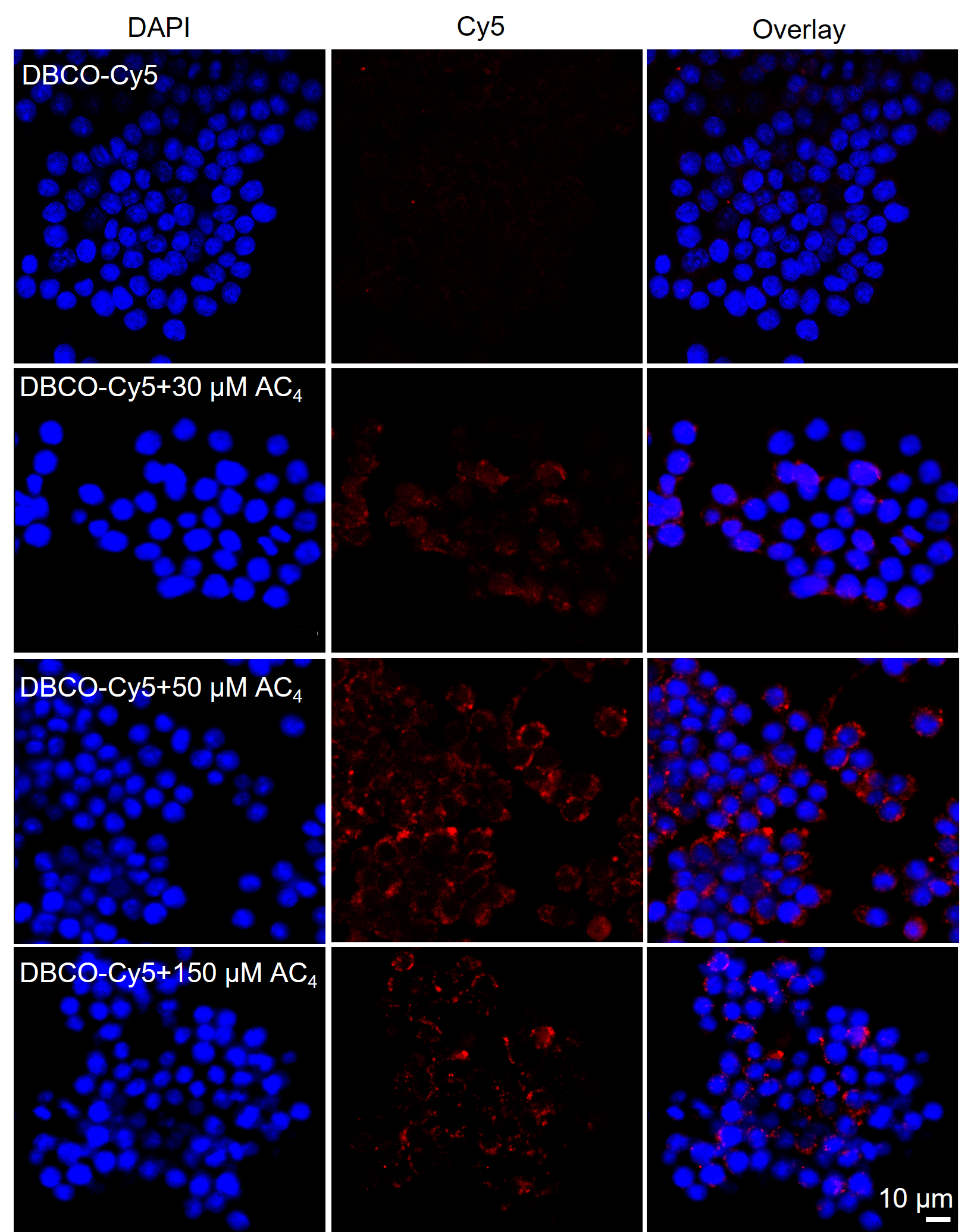


Figure S2 Confocal microscopy observation of Mφ_N3_ labeled with different concentrations of AC_4_ManNAz (30 μM, 50 μM, and 150 μM). AC_4_: AC_4_ManNAz. Scale bar: 10 μm.


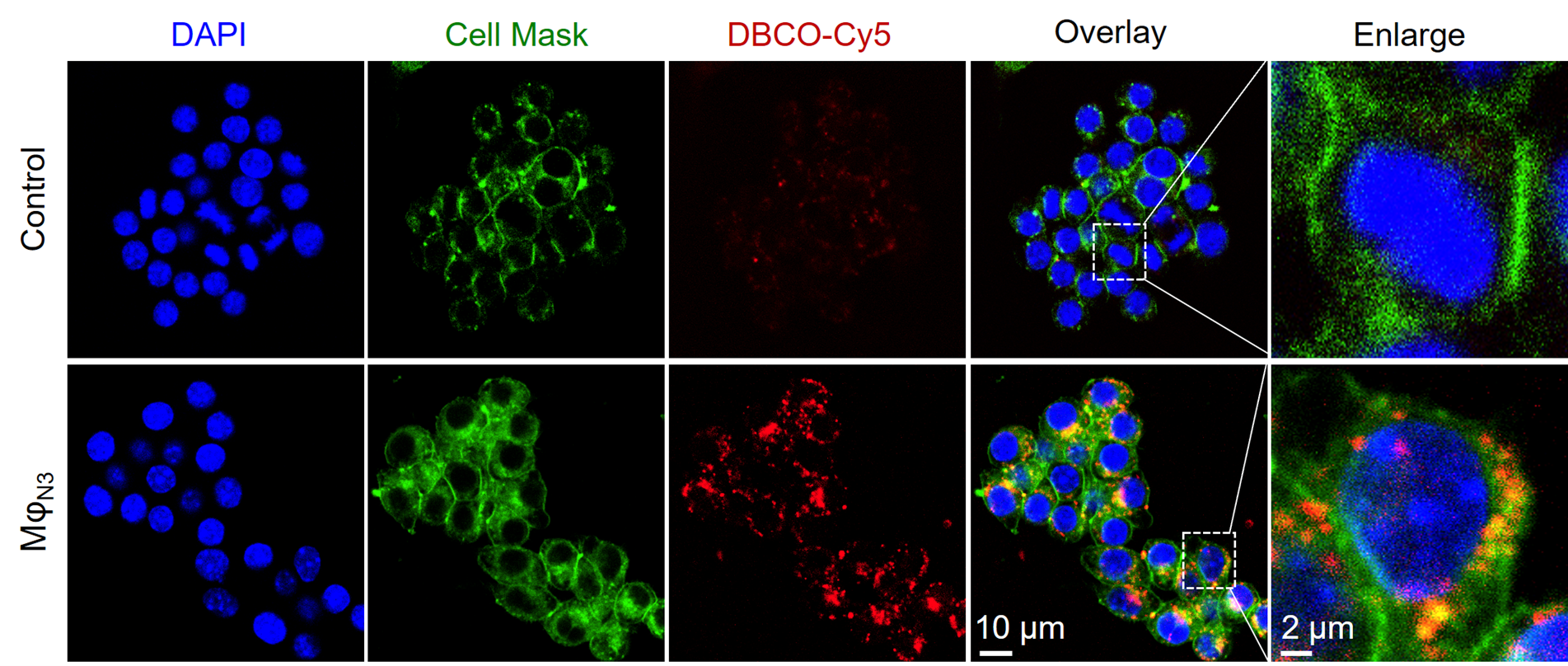


Figure S3 Laser confocal microscopy analysis of the co-localization of Mφ_N3_ cell membranes with DBCO-Cy5. Scale bar: 10 μm; scale bar for enlarged images: 2 μm.


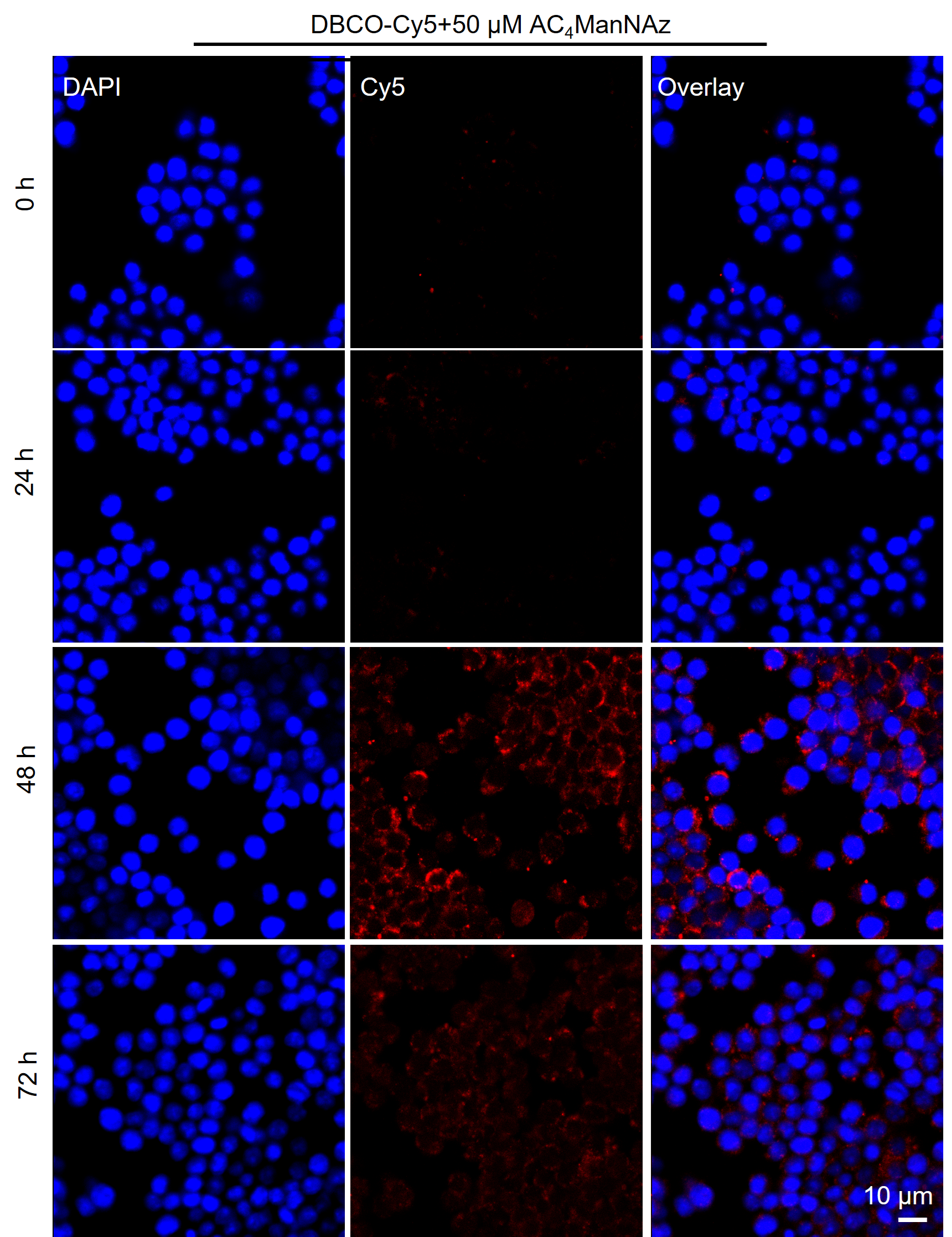


Figure S4 Laser confocal observation of the effects of AC_4_ManNAz incubation for 0, 24, 48, and 72 h on metabolic labeling of RAW264.7 cells, scale bar: 10 μm.


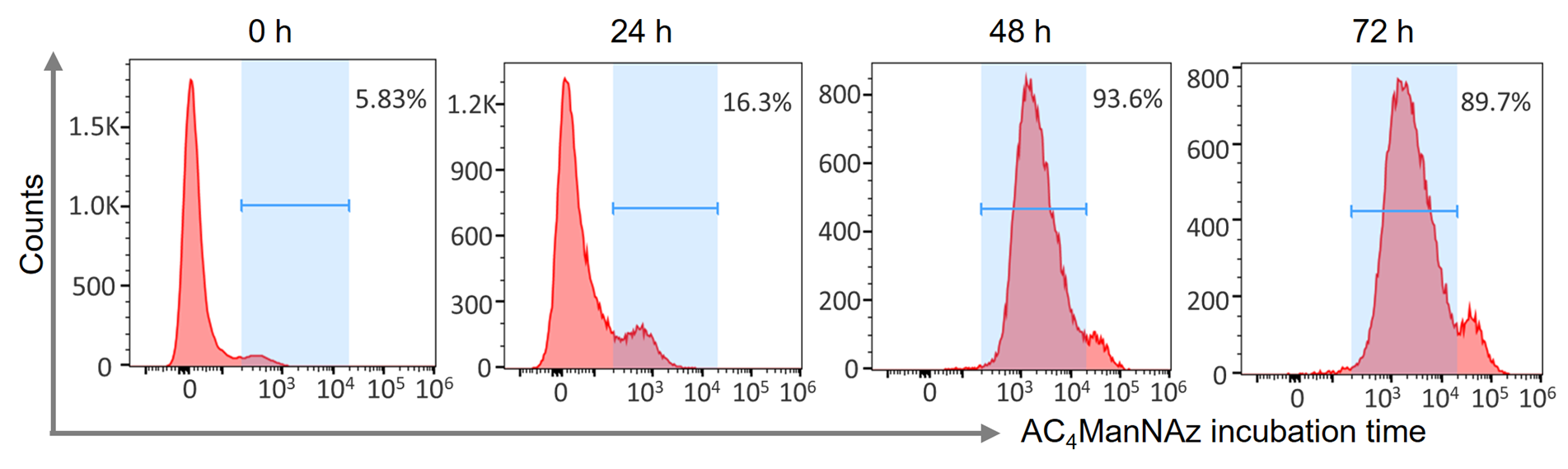


Figure S5 Flow cytometry detection of metabolic labeling effects on RAW264.7 cells after incubation with AC_4_ManNAz for 0, 24, 48, and 72 h.


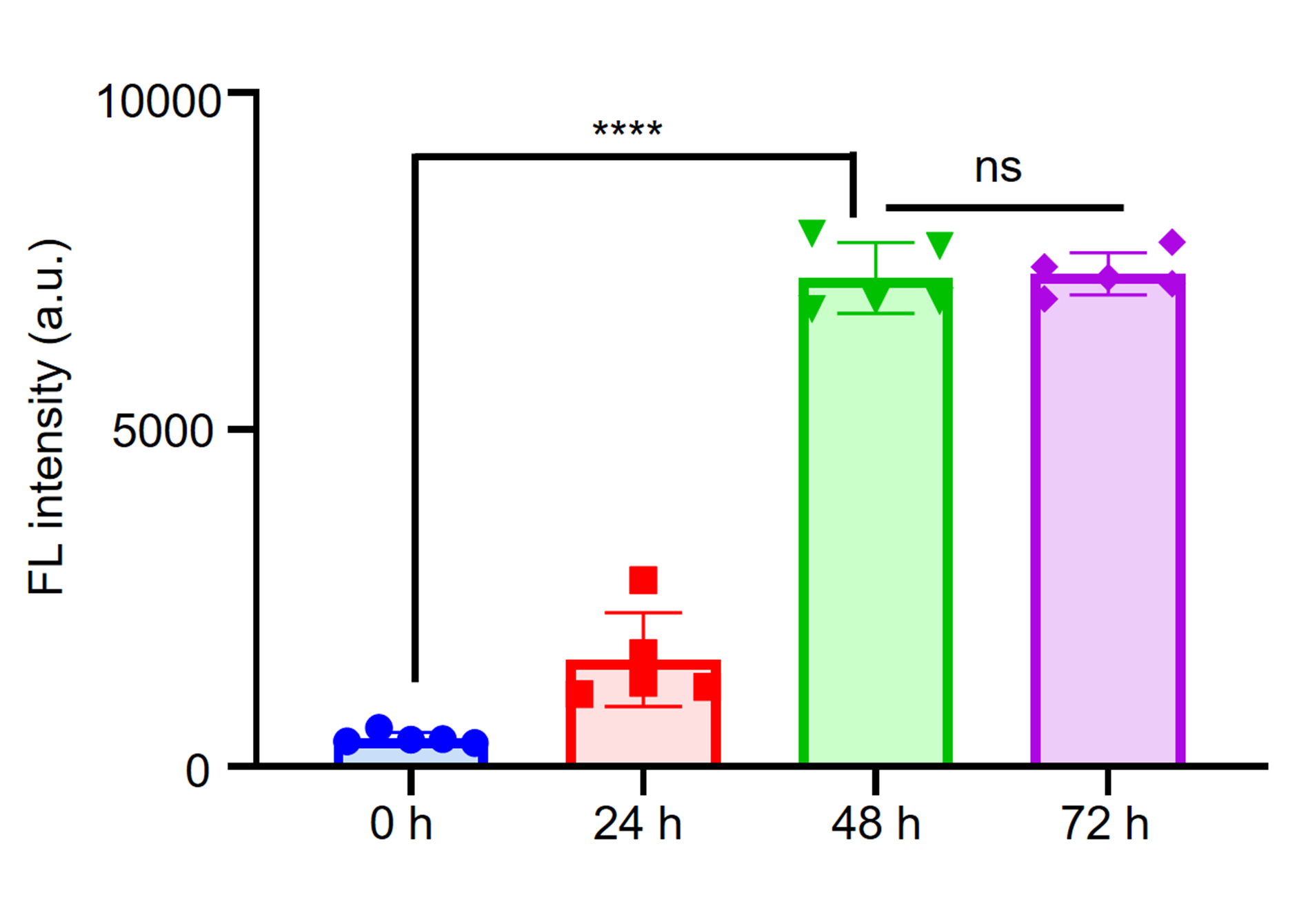


Figure S6 Evaluation of the effects of AC_4_ManNAz incubation time on metabolic labeling of RAW264.7 cells using a fluorescence microplate reader. All data are presented as mean ± standard deviation, n = 5; ns indicates no statistical significance, *****P* < 0.0001. For normally distributed datasets, statistical analyses included Student's t-test (for two-group comparisons) or one-way ANOVA with Dunnett’s *post-hoc* test.


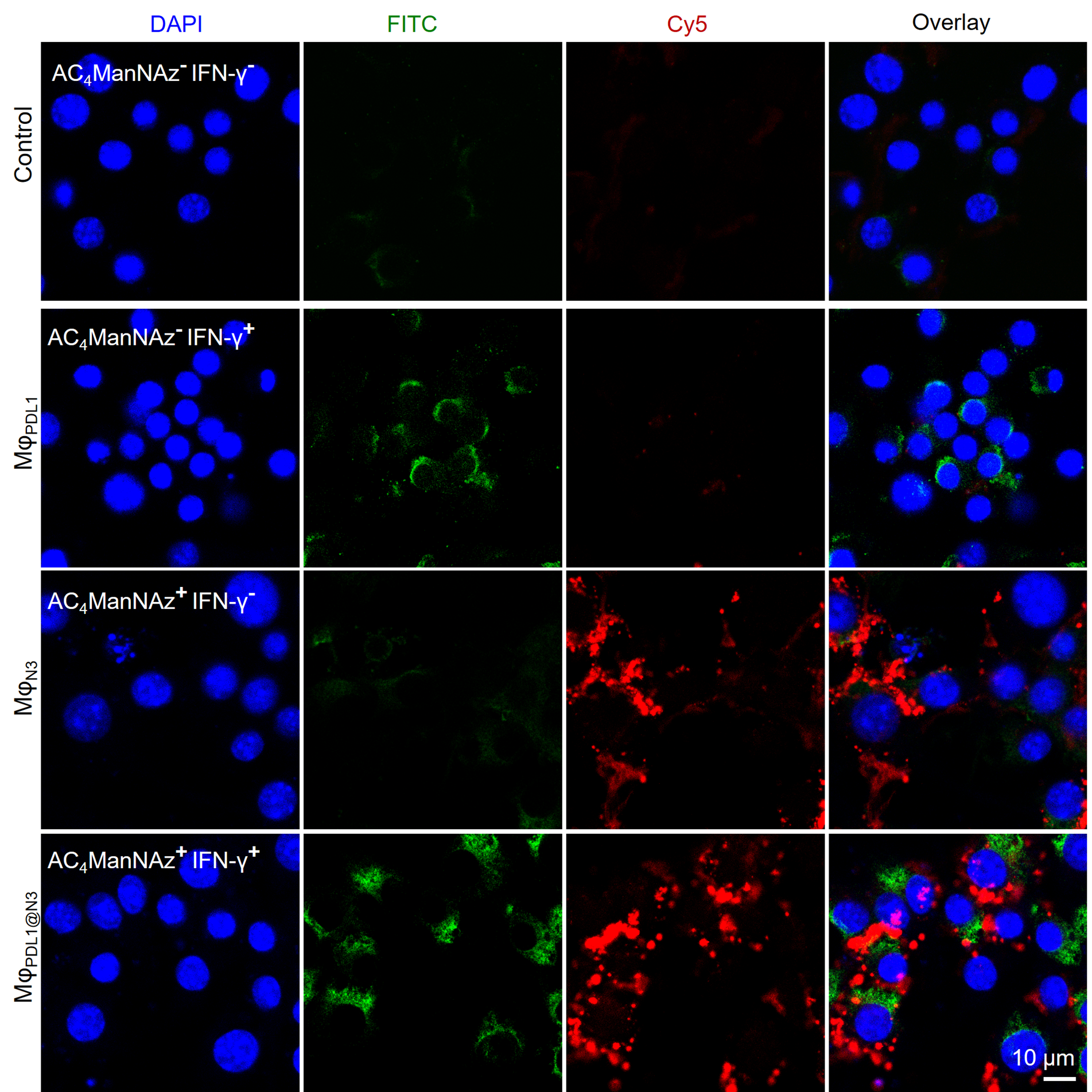


Figure S7 Induction of Mφ_PD-L1@N3_ construction by co-treatment with 100 ng/mL IFN-γ and 50 μM AC_4_ManNAz, visualized by laser confocal microscopy, scale bar: 10 μm.


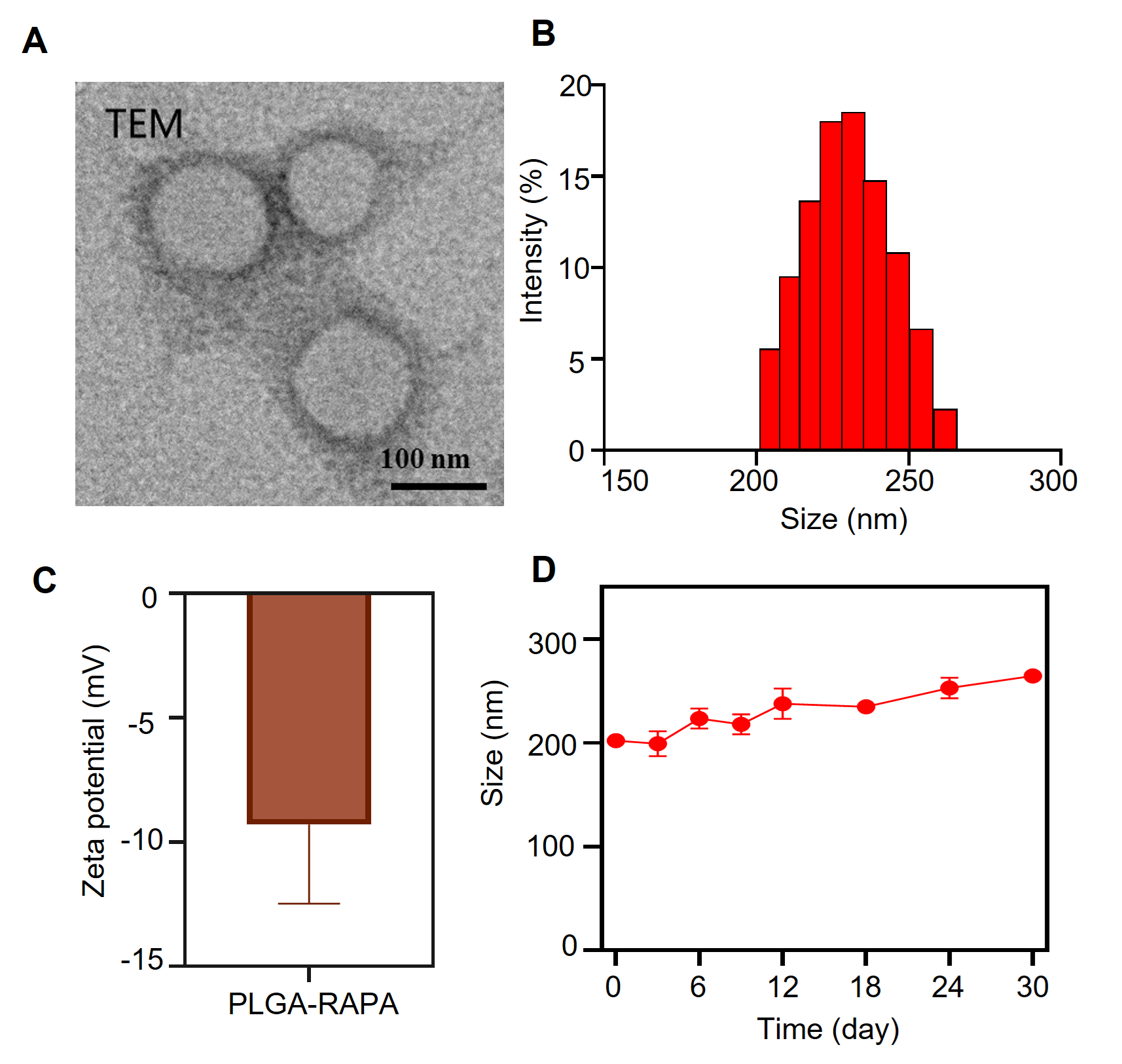


Figure S8 Transmission electron microscopy images (A), particle size distribution (B), Zeta potential (C), and particle size changes over 30 days (D) of RAPA NPs, scale bar: 100 nm, n = 3.


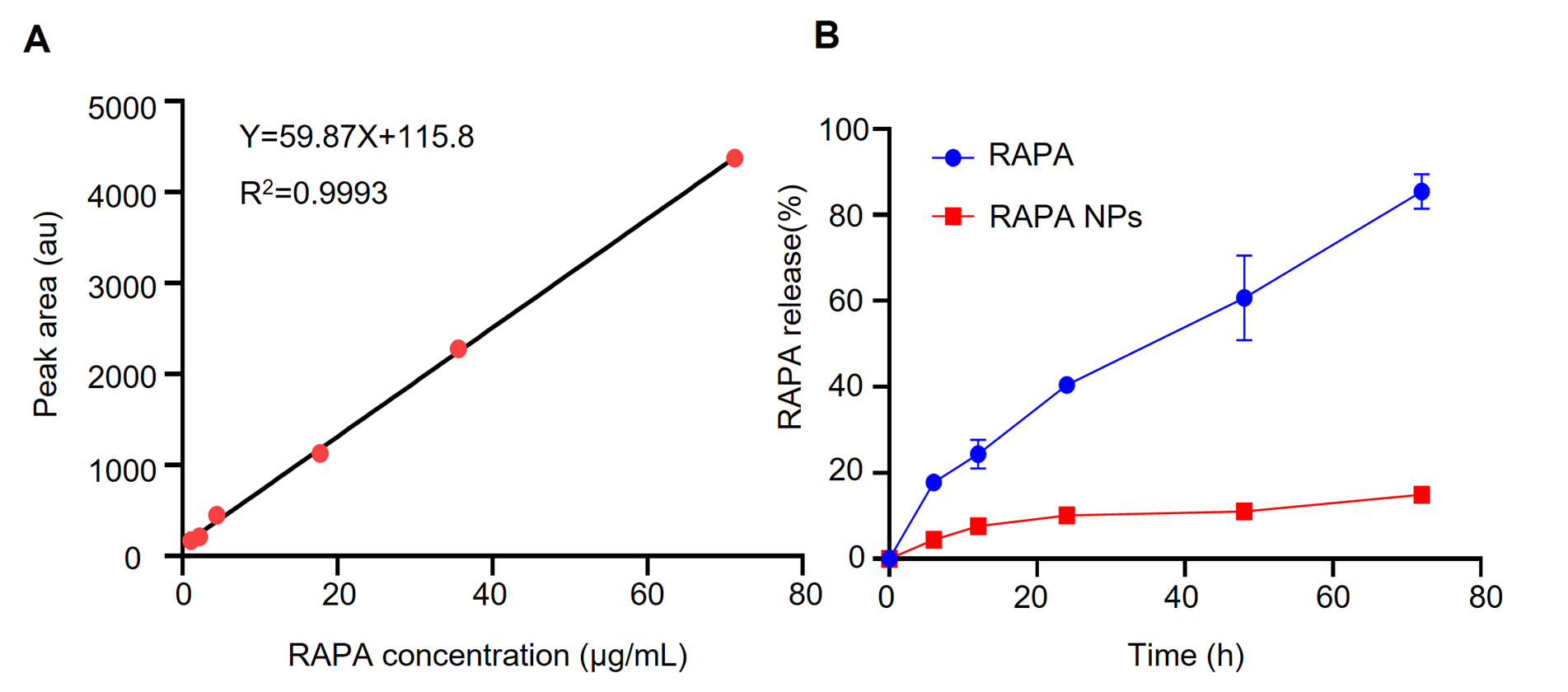


Figure S9 Standard curve of RAPA (A) and drug release profile curve (B), n=3.


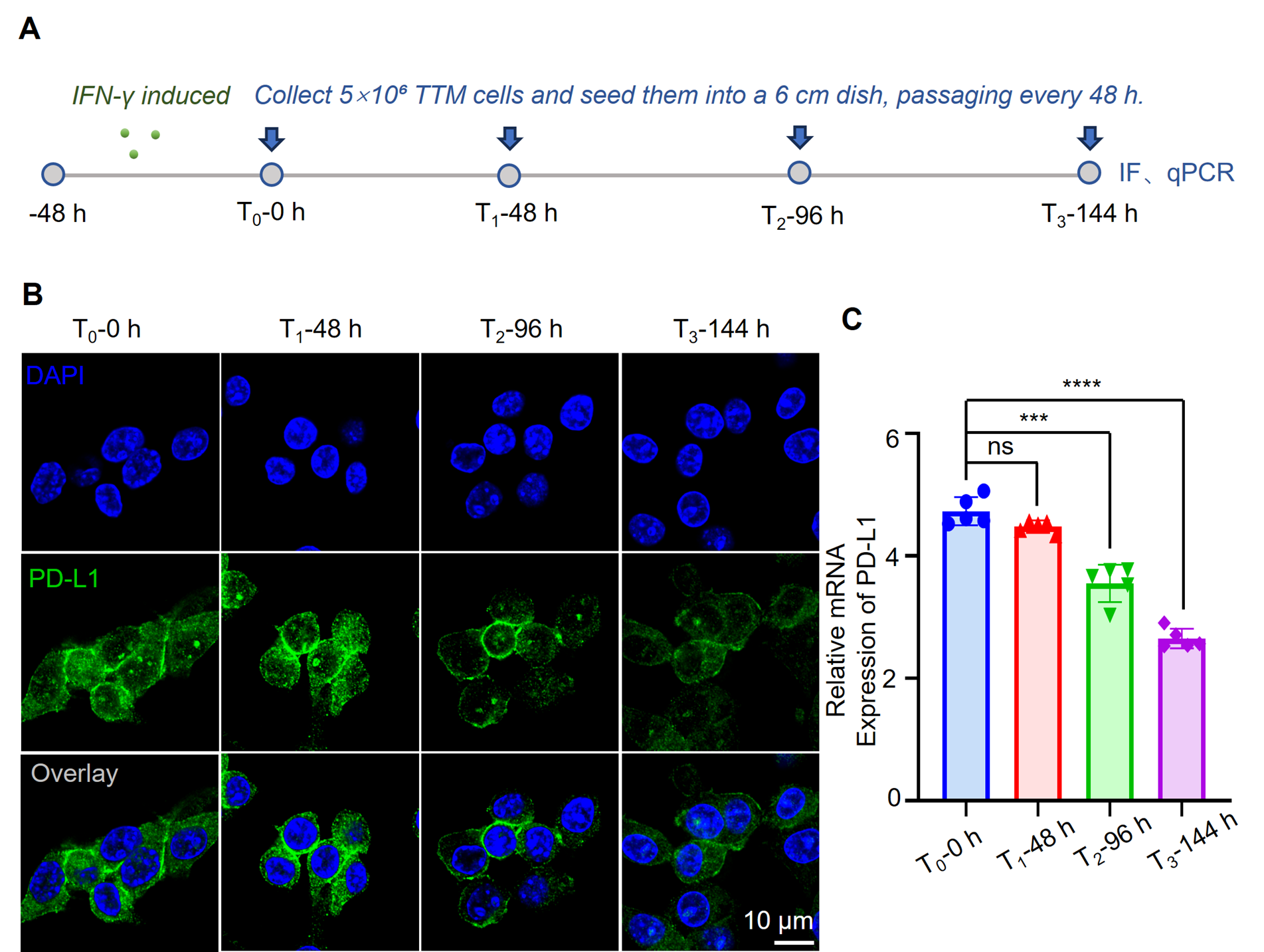


**Figure S10** Assessment of PD-L1 expression dynamics in TTM cells during serial passaging. (A) Experimental timeline showing cell collection at T_0_ (0 h), T_1_ (48 h), T_2_ (96 h), and T_3_ (144 h) passages. (B) Immunofluorescence analysis and (C) qPCR quantification of PD-L1 expression levels at each passage time point. All data are presented as mean ± SEM. n=5, ns represents no statistically significant difference, ****P* < 0.001, *****P* < 0.0001. Repeated-measures one-way ANOVA with Dunnett’s *post-hoc* test for panels (C).


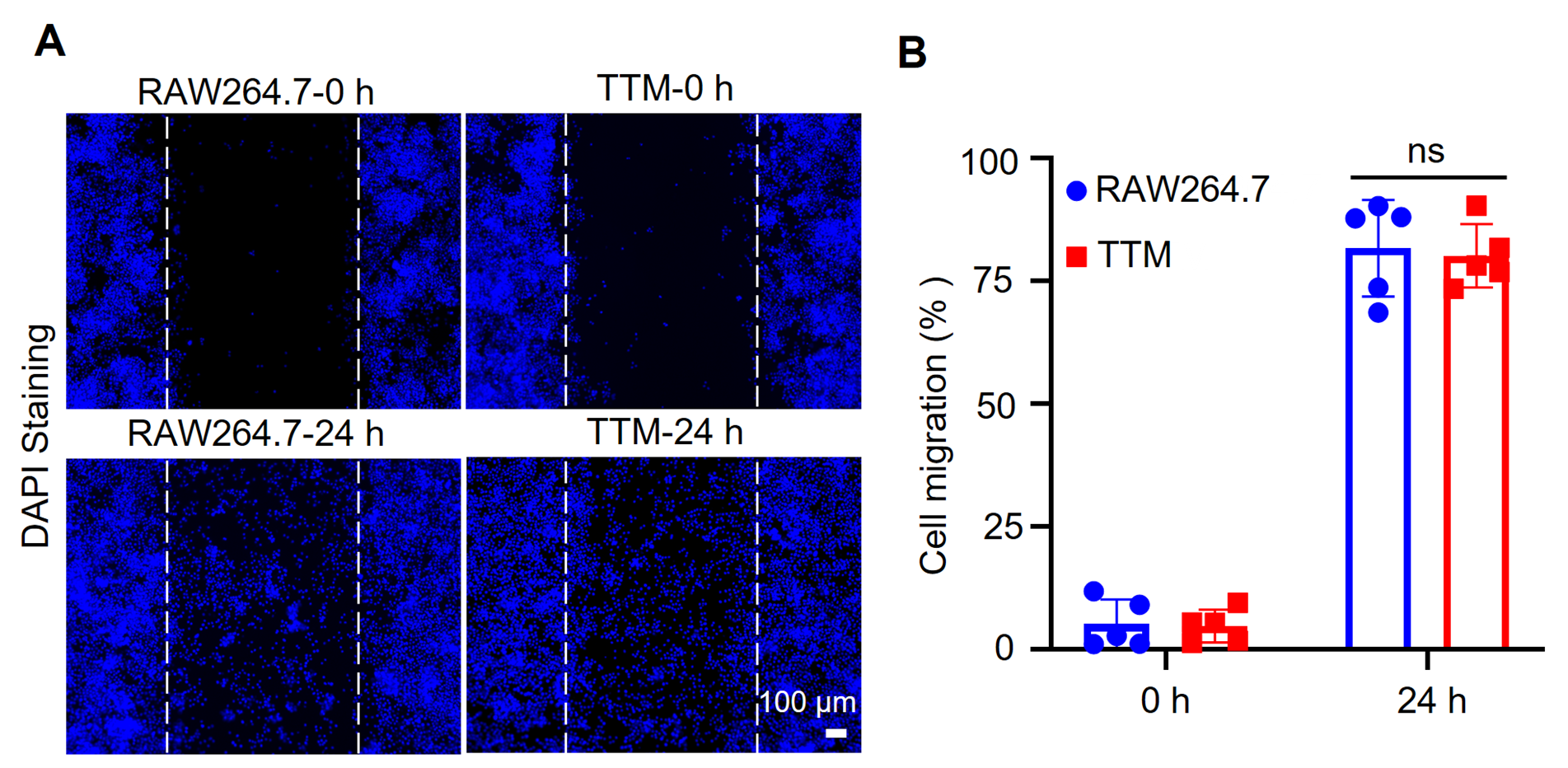


**Figure S11** Assessment of migratory capacity of RAW264.7 and TTM cells via scratch assay. (A) Cells were stimulated with 10 ng/mL MCP-1 to mimic an inflammatory microenvironment in vitro. Cell migration was visualized at 0 h and 24 h post-induction using DAPI staining and fluorescence microscopy. Scale bar=100 μm. (B) Quantitative analysis of cell migration rate was performed using the edge detection method in ImageJ. Data are presented as mean ± SEM. Two-tailed unpaired t test with Welch’s correction for (B). n = 5, ns indicates no statistically significant difference.


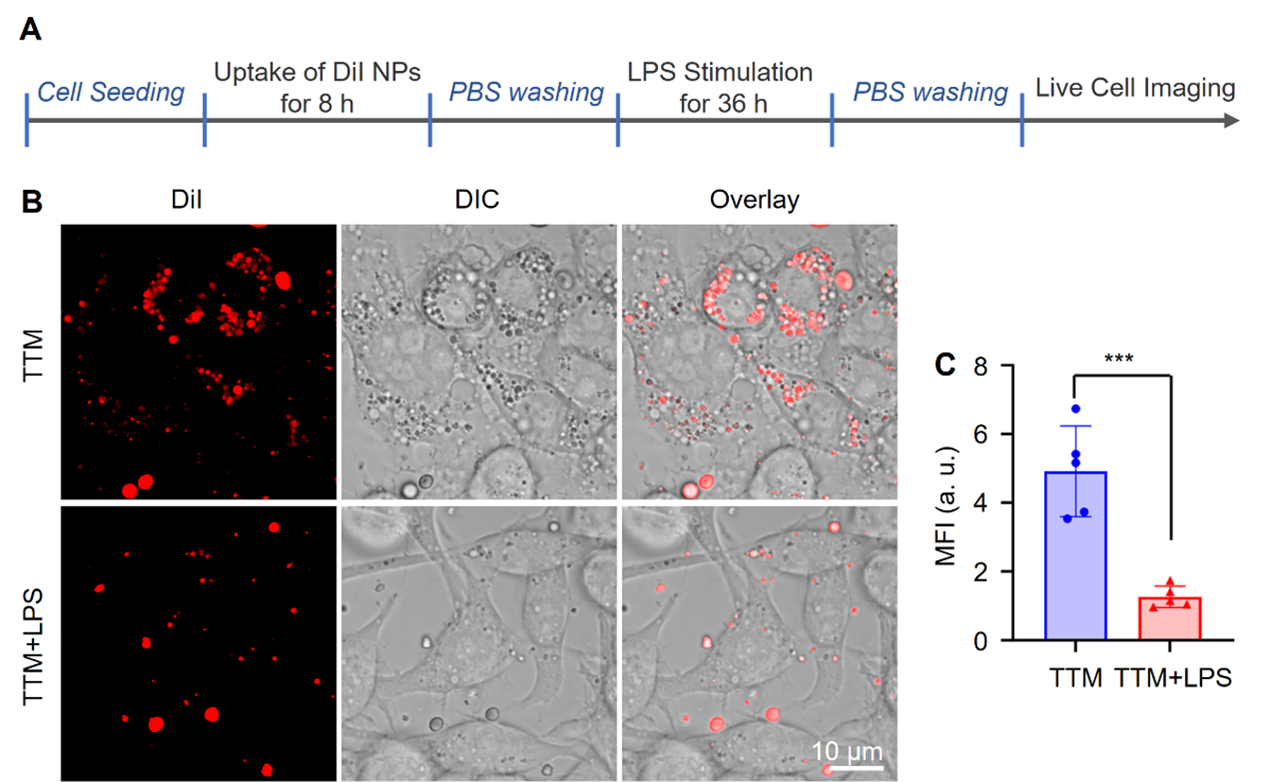


**Figure S12** Evaluation of the release capability of DiI-NP following LPS stimulation of TTM cells. (A-B) After LPS stimulation, the cells were rinsed with PBS to remove any free drug, and the intracellular nanoparticle loading was further examined using laser confocal microscopy. Scale bar=10 μm. (C) Quantification of the intracellular DiI fluorescence by measuring the average fluorescence intensity using the ImageJ software. Data are presented as mean ± SEM. n=5, ****P* < 0.001. Two-tailed unpaired t test with Welch’s correction for (C).


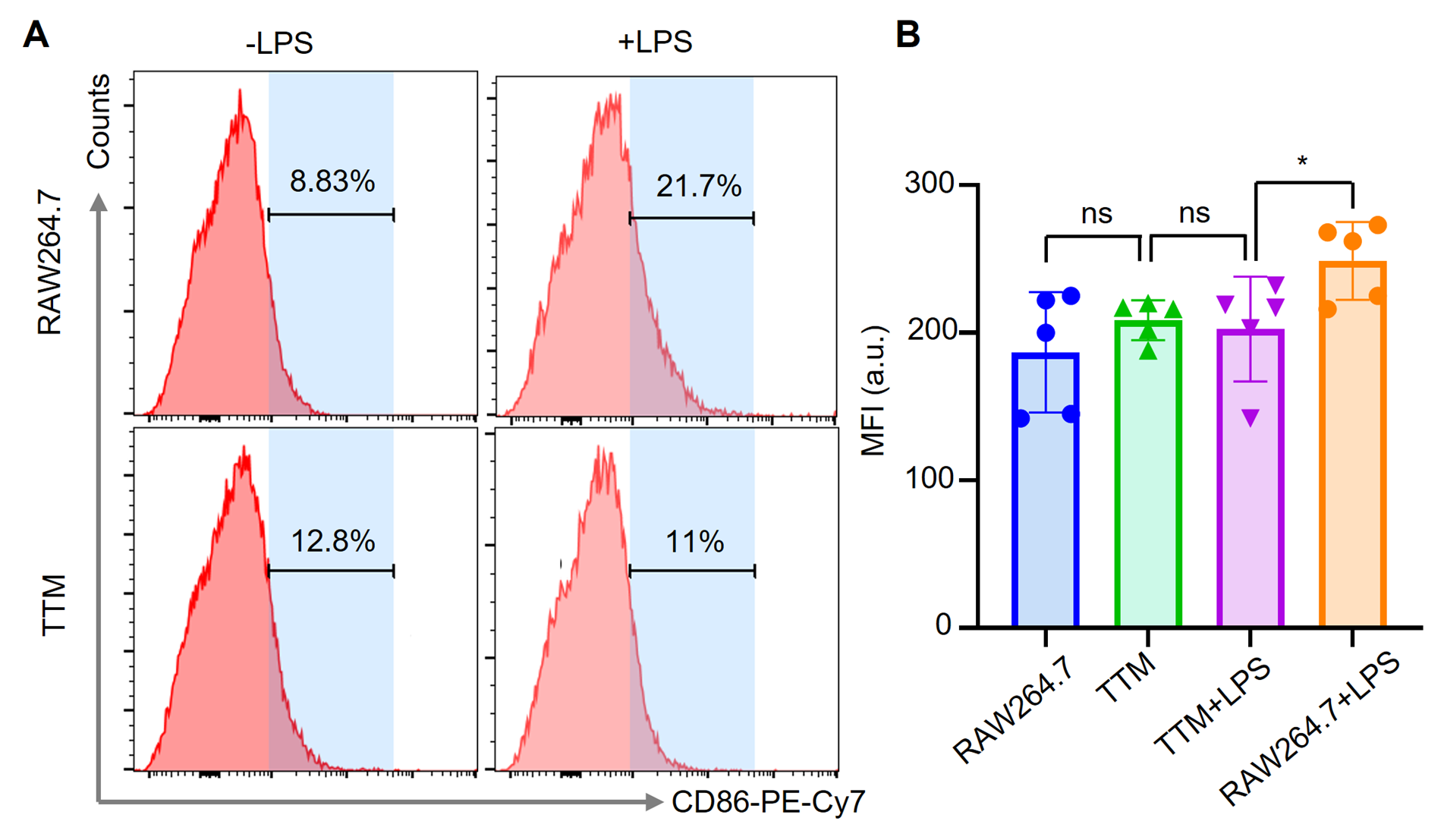


**Figure S13** Flow cytometric analysis of the effect of LPS stimulation on the M1 phenotype of TTM cells. (A) Histogram analysis of CD86 expression in M1 cells. (B) The mean fluorescence intensity (MFI) of PE-Cy7-labeled CD86. All data are expressed as mean ± SEM. n=5, 'ns' indicates no statistical difference. Statistical significance was defined as **P* < 0.05. For normally distributed datasets, statistical analyses included Student's t-test (for two-group comparisons) or one-way ANOVA with Dunnett’s *post-hoc* test.


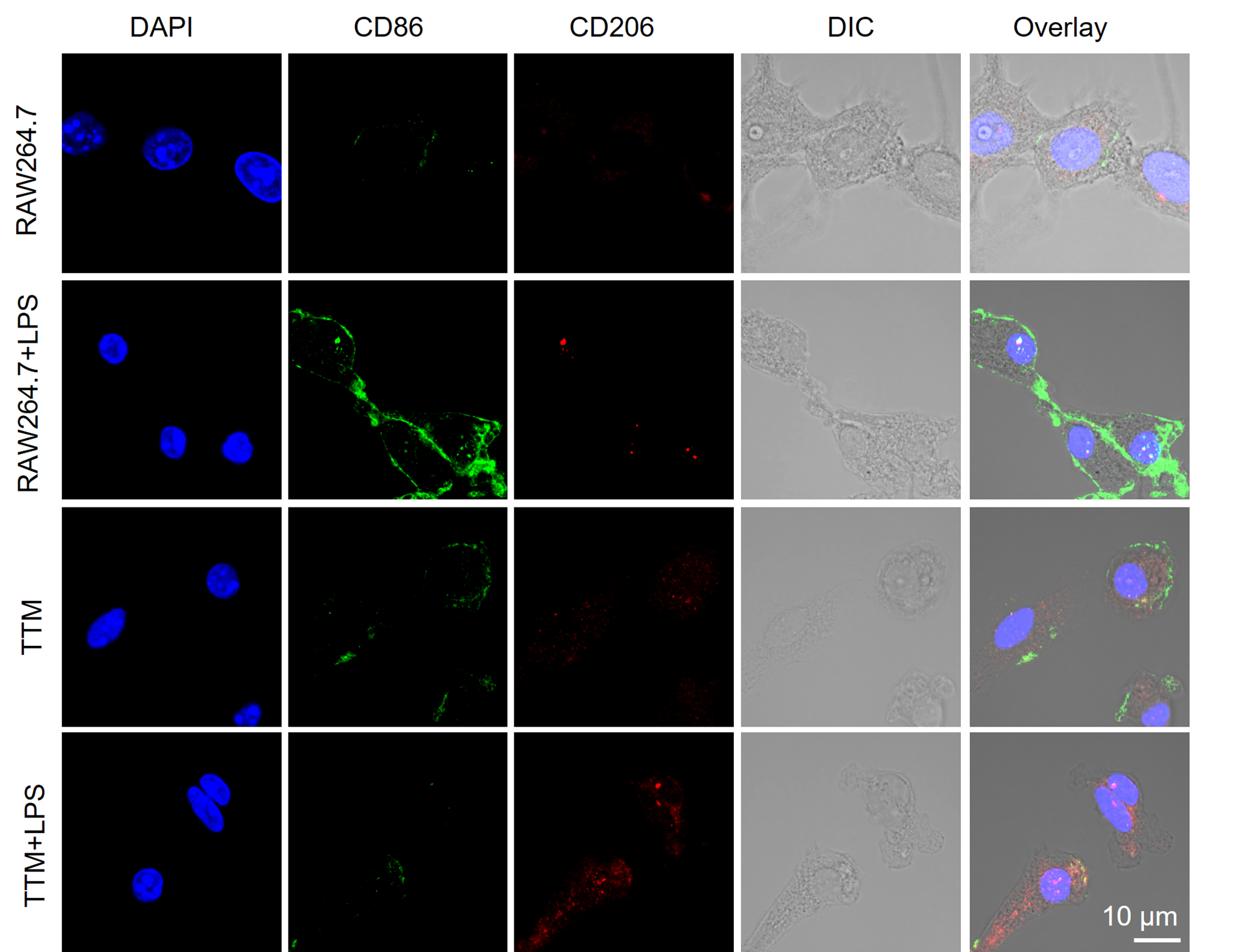


**Figure S14** Immunofluorescence analysis of macrophage polarization markers in TTM cells, showing CD86 (M1) and CD206 (M2). Scale bar: 10 μm.


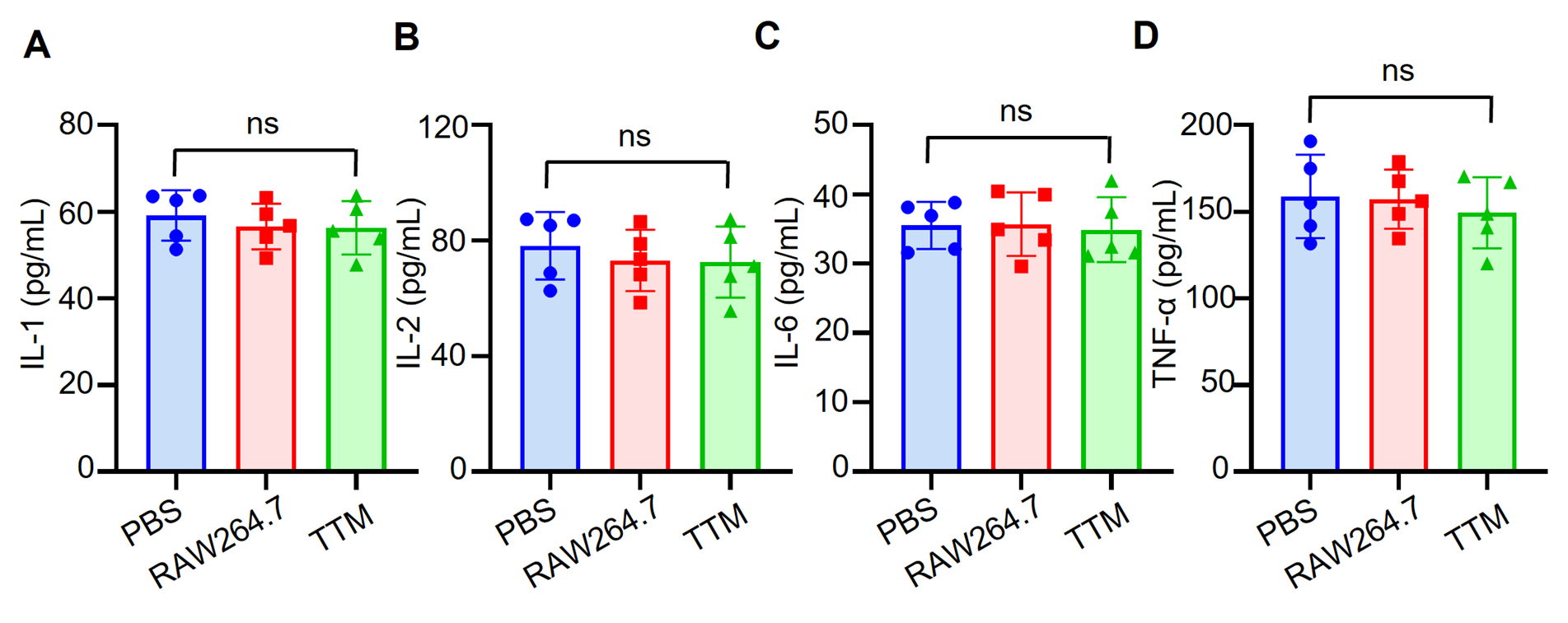


**Figure S15** Elisa detection of mouse serum IL-1 (A), IL-2 (B), IL-6 (C), and TNF-α (D) levels. n=5, 'ns' indicates no statistical difference. Data are presented as mean ± SEM. Repeated-measures one-way ANOVA with Dunnett’s *post-hoc* test.


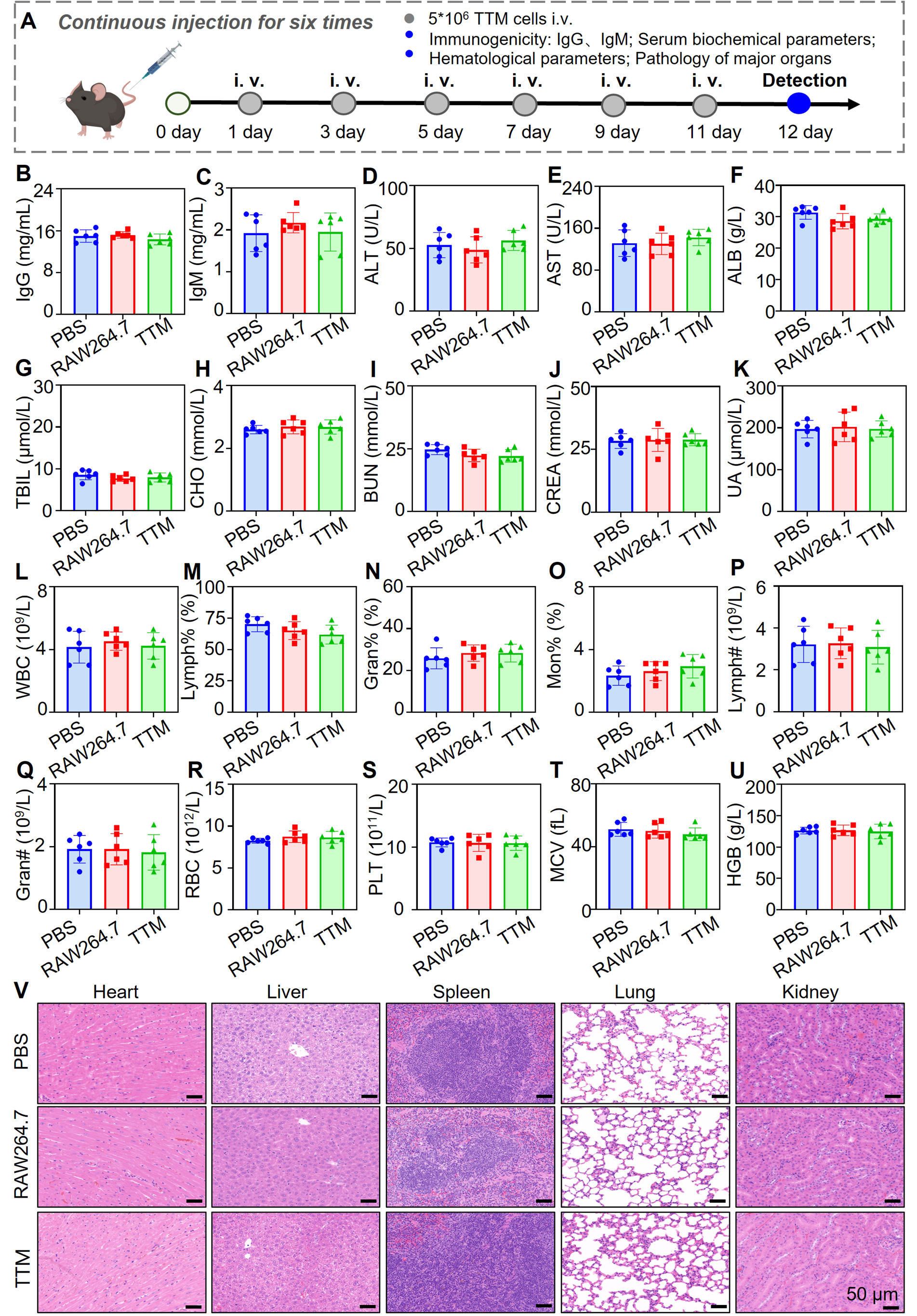


**Figure S16** **Extended safety evaluation after 6 injections.** (A) C57BL/6J mice received intravenous injections via tail vein of either PBS, 5 × 10^6^ RAW264.7 cells, or 5 × 10^6^ **TTM** cells every 48 h for a total of 6 administrations. On Day 12, blood and organs were collected for safety assessment; (B-C) Serum IgG and IgM levels (ELISA); (D-H) Liver function parameters: Alanine aminotransferase (ALT), aspartate aminotransferase (AST), albumin (ALB), total bilirubin (TBIL), and total cholesterol (CHO); (I-K) Kidney function parameters: Blood urea nitrogen (BUN), creatinine (CREA), and uric acid (UA); (L–U) Hematological parameters, including white blood cell, lymphocyte, granulocyte, monocyte, red blood cell, platelet counts, mean corpuscular volume, and hemoglobin concentration. (V) Representative H&E staining of heart, liver, spleen, lung, and kidney. Scale bar: 50 μm. n = 6 mice per group. Individual scatter points represent independent biological replicates, with data presented as mean ± SEM. These quantitative data presented showed no statistically significant differences between groups (*P* > 0.05). Repeated-measures one-way ANOVA with Dunnett’s *post-hoc* test for panels (B-U).


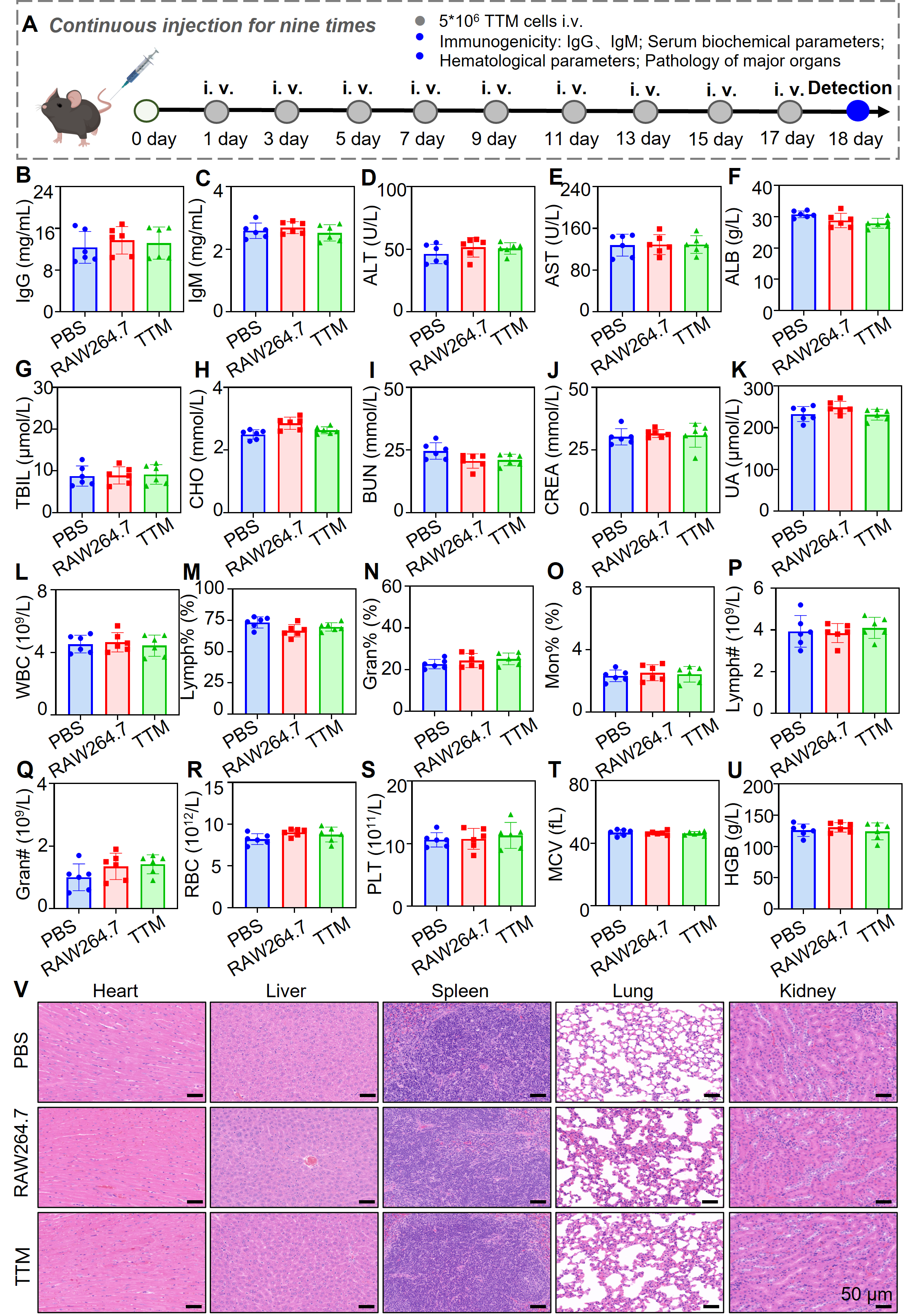


**Figure S17** **Extended safety evaluation after 9 injections.** (A) C57BL/6J mice received intravenous injections via tail vein of either PBS, 5 × 10^6^ RAW264.7 cells, or 5 × 10^6^ **TTM** cells every 48 h for a total of 9 administrations. On Day 18, blood and organs were collected for safety assessment; (B-C) Serum IgG and IgM levels (ELISA); (D-H) Liver function parameters: Alanine aminotransferase (ALT), aspartate aminotransferase (AST), albumin (ALB), total bilirubin (TBIL), and total cholesterol (CHO); (I-K) Kidney function parameters: Blood urea nitrogen (BUN), creatinine (CREA), and uric acid (UA); (L–U) Hematological parameters, including white blood cell, lymphocyte, granulocyte, monocyte, red blood cell, platelet counts, mean corpuscular volume, and hemoglobin concentration. (V) Representative H&E staining of heart, liver, spleen, lung, and kidney. Scale bar: 50 μm. n = 6 mice per group. Individual scatter points represent independent biological replicates, with data presented as mean ± SEM. These quantitative data presented showed no statistically significant differences between groups (*P* > 0.05). Repeated-measures one-way ANOVA with Dunnett’s *post-hoc* test for panels (B-U).


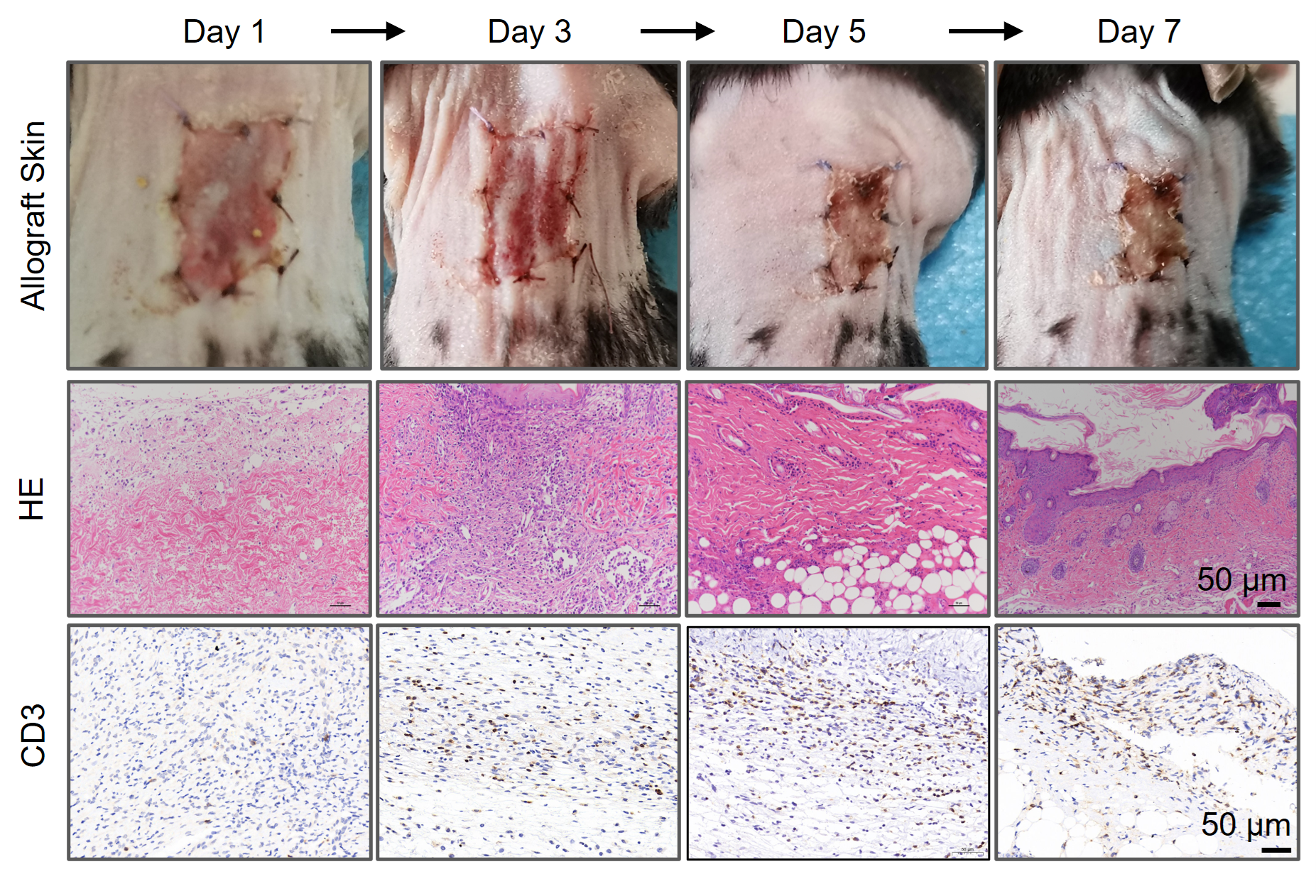


**Figure S18** Observations of allograft mouse skin and histological immunohistochemical staining of the graft tissue after transplantation for 1, 3, 5, and 7 d. Scale bar: 50 μm.


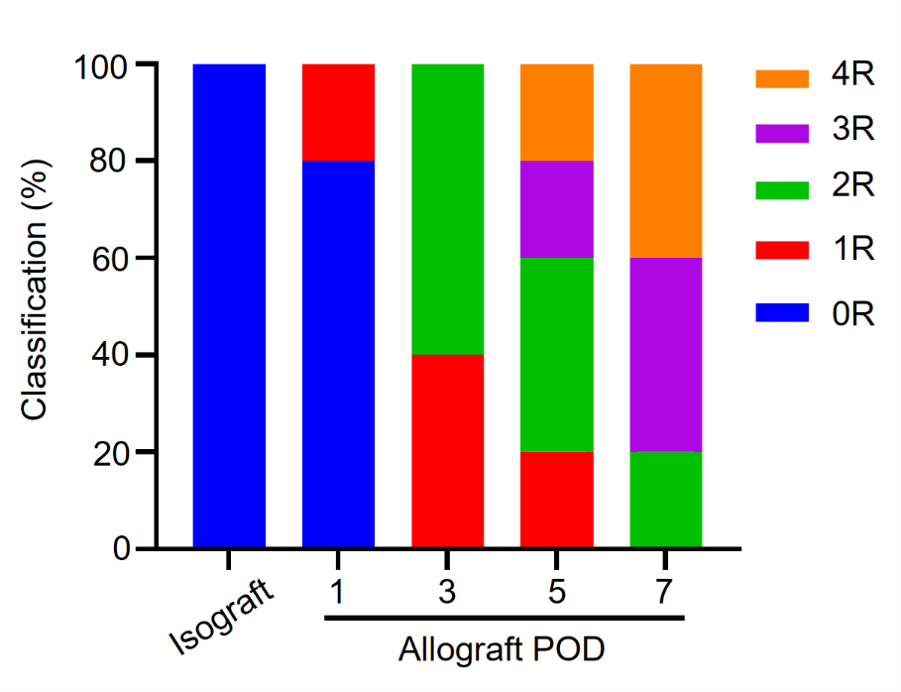


**Figure S19** Assessment of skin rejection grades in isograft and allograft mice based on the Banff classification. The allograft mice were evaluated at postoperative day (POD) 1, 3, 5 and 7. n=5.


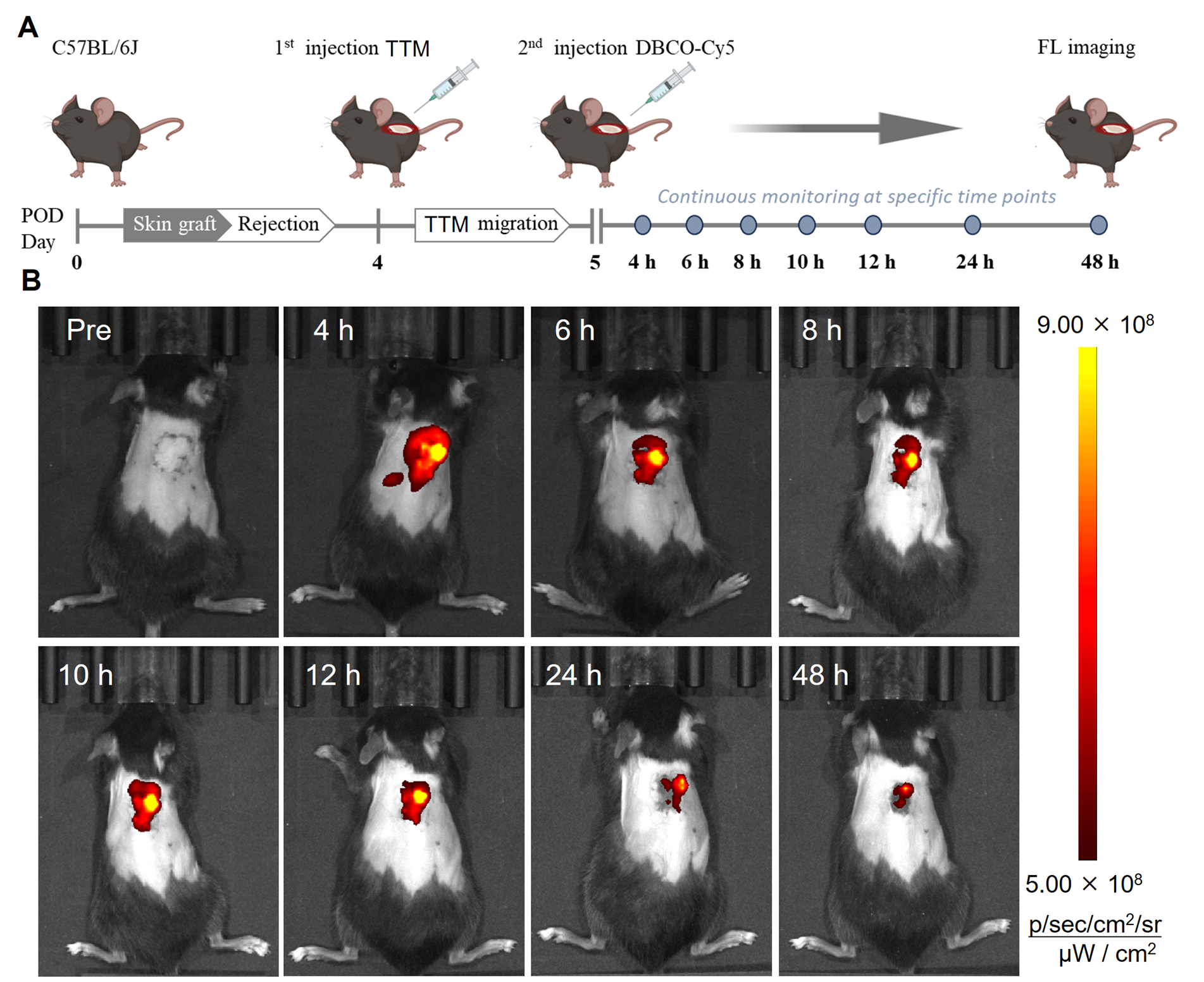


Figure S20 On day 4 post-operation (POD 4), transplant recipient mice were intravenously injected with TTM, followed by intravenous injection of DBCO-Cy5 24 h later. Real-time imaging was conducted at multiple time points thereafter. Panel (A) illustrates a schematic of real-time in vivo imaging monitoring the migration of TTM. Panel (B) presents real-time fluorescence imaging at 4, 6, 8, 10, 12, 24, and 48 h following DBCO-Cy5 injection. n=5.


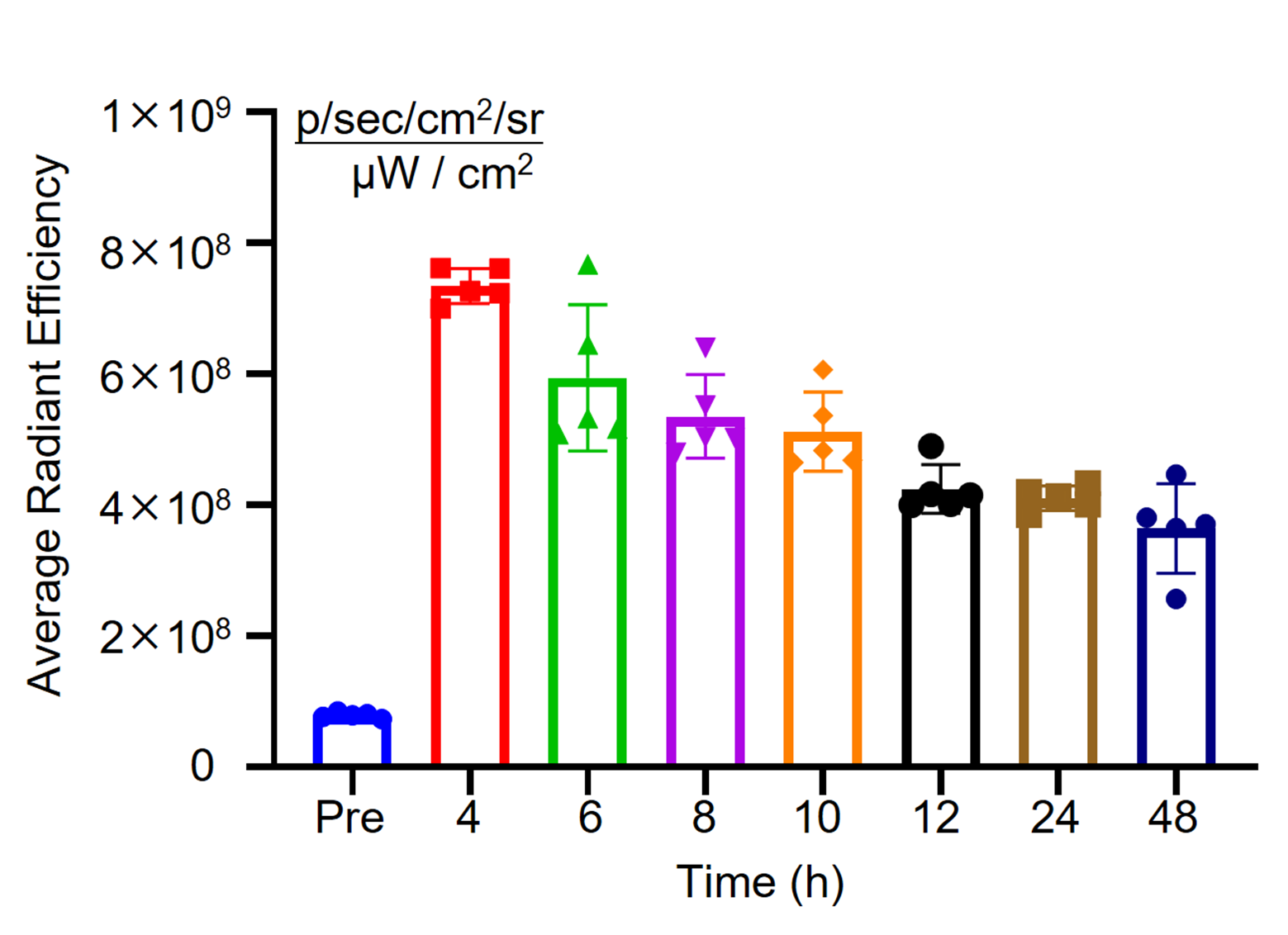


**Figure S21** Quantitative statistical analysis of the allograft’s fluorescence intensity at 4 h, 6 h, 8 h, 10 h, 12 h, 24 h, and 48 h after DBCO-Cy5 injection (corresponding to **Figure S20B**). Data represent n=5 biological replicates.


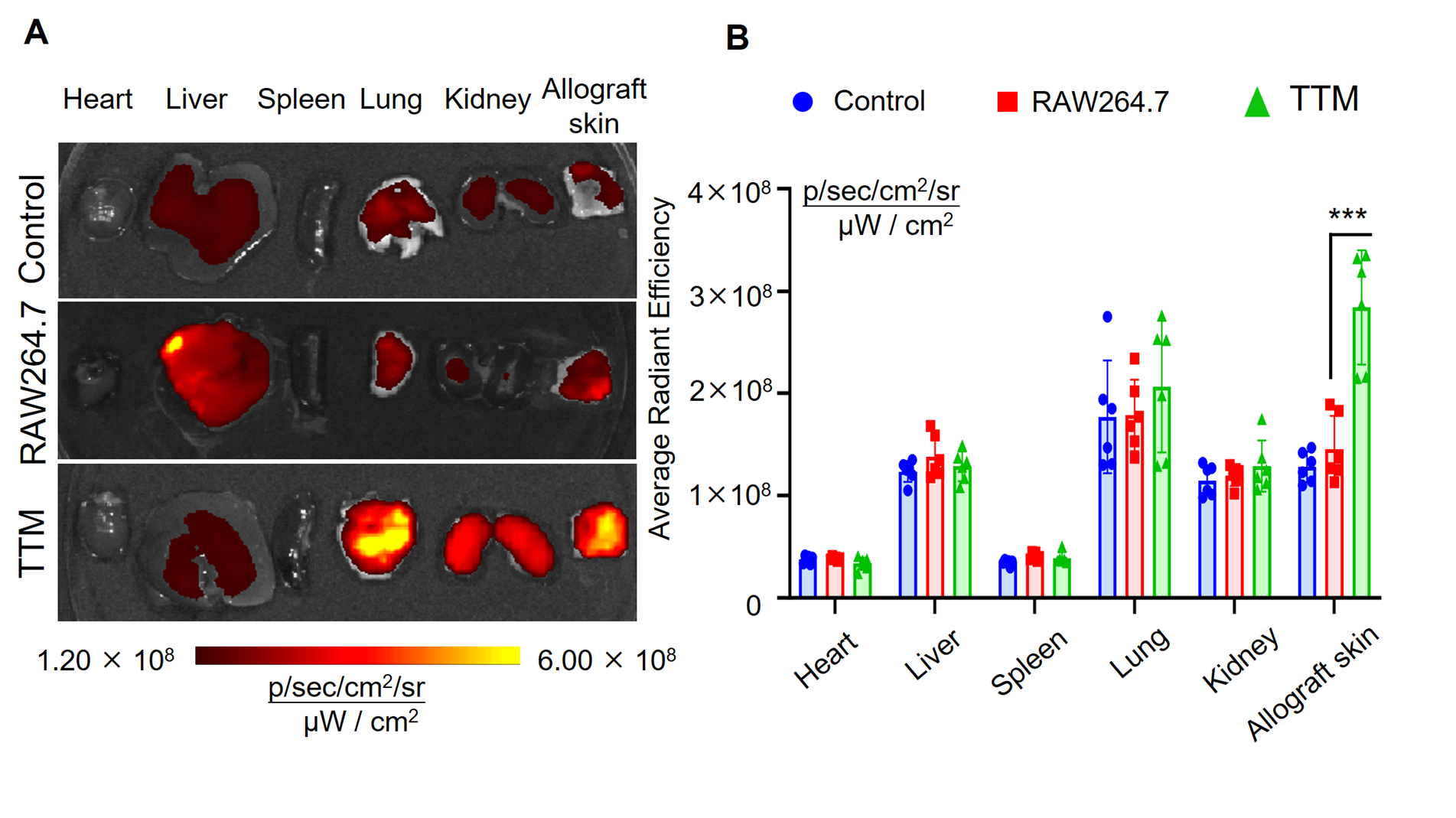


**Figure S22** (A) TTM was intravenously injected on day 4 post-transplantation (POD 4), followed by DBCO-Cy5 injection 24 h later. 6 h after the DBCO-Cy5 reaction, the major organs of the mice were collected for fluorescence imaging (POD 5). (B) And the fluorescence intensity was conducted, n=6, ****P* < 0.001. Individual scatter points represent independent biological replicates, with data presented as mean ± SEM. Two-tailed unpaired t test with Welch’s correction for (B).


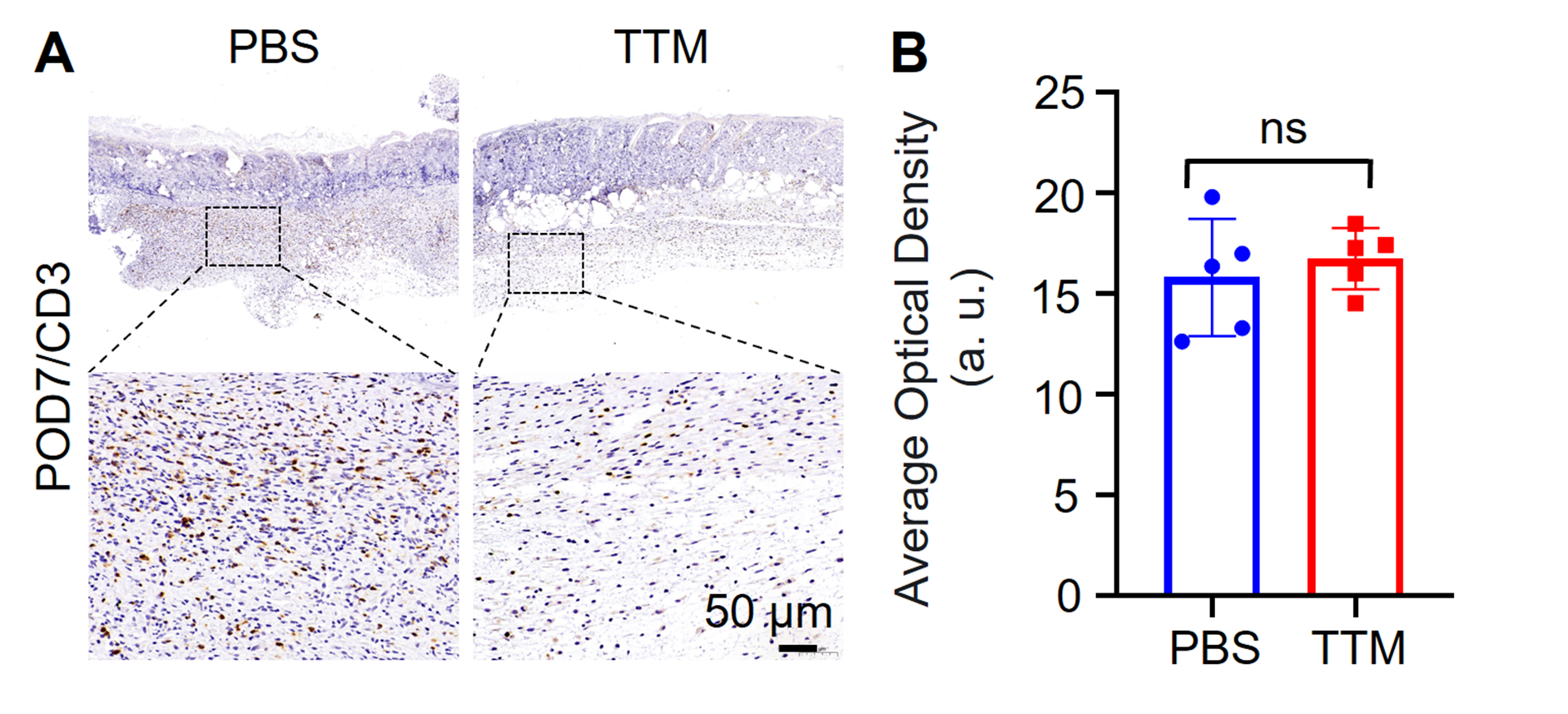


**Figure S23** (A) CD3 immunohistochemical images of the grafts on day 7 post-transplantation after PBS or **TTM** cell tail vein injection. Scale bar = 50 μm. (B) Detection of the average optical density of CD3-positive areas in the grafts, n=5. ns indicates no statistically significant difference. Data presented as mean ± SEM. Two-tailed unpaired t test with Welch’s correction for (B).


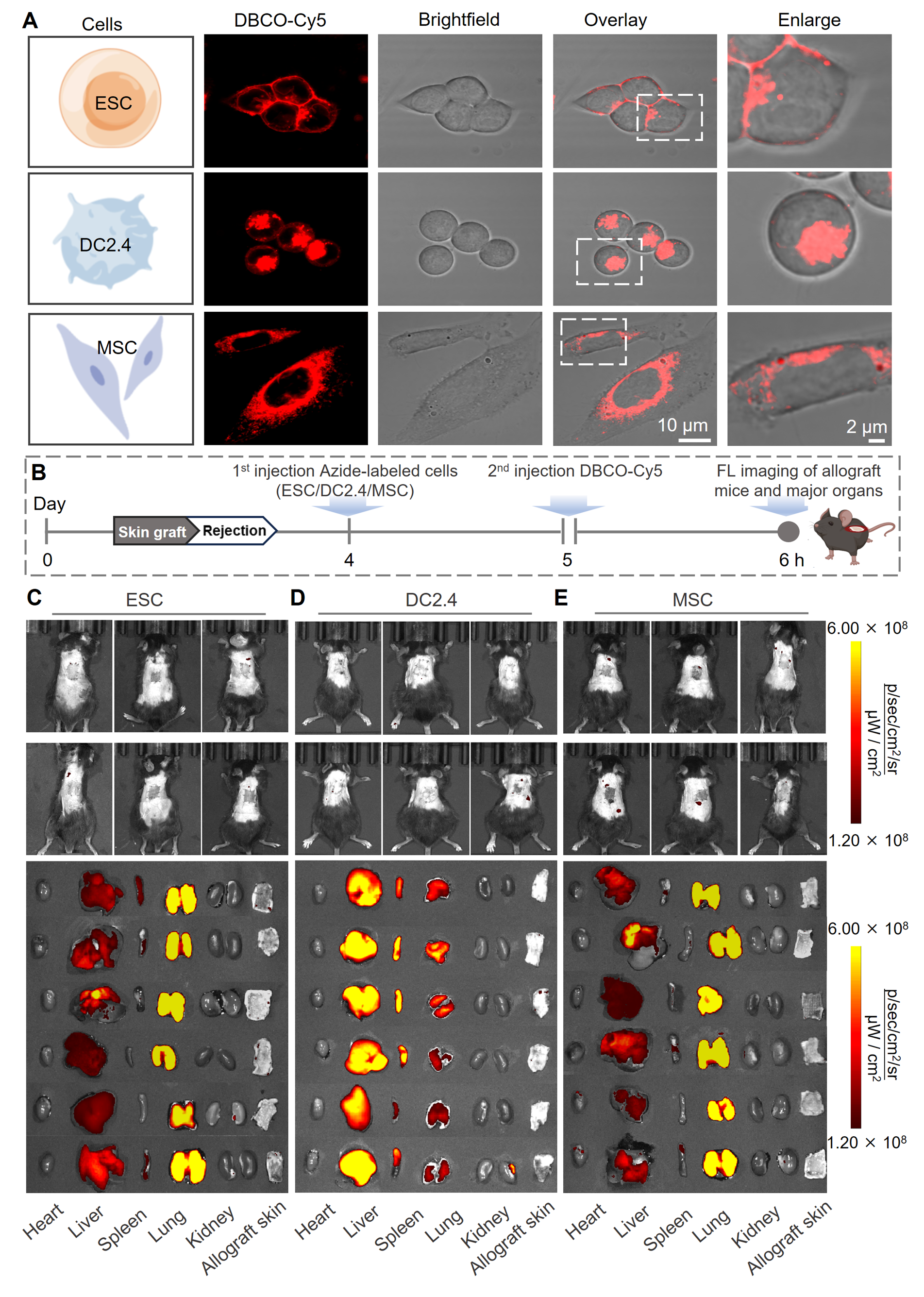


**Figure S24** **Evaluation of biodistribution specificity across different cell types.** (A) In vitro labeling of ESC cells, DC2.4 cells, and MSC cells with -N_3_ (red) observed by laser confocal microscopy. (B) Skin allograft model mice were injected with ESC cells (C), DC2.4 cells (D), and MSC cells (E) on the 4th day post-transplantation, followed by DBCO-Cy5 injection on the 5th day post-transplantation. In vivo imaging and fluorescence imaging of major organs were performed 6 h after injection. The intravenous injection dosage was 5×10^6^ cells per mouse. n=6. The cell elements involved in Figure S24(A) were created with [biogdp.com](http://biogdp.com/).


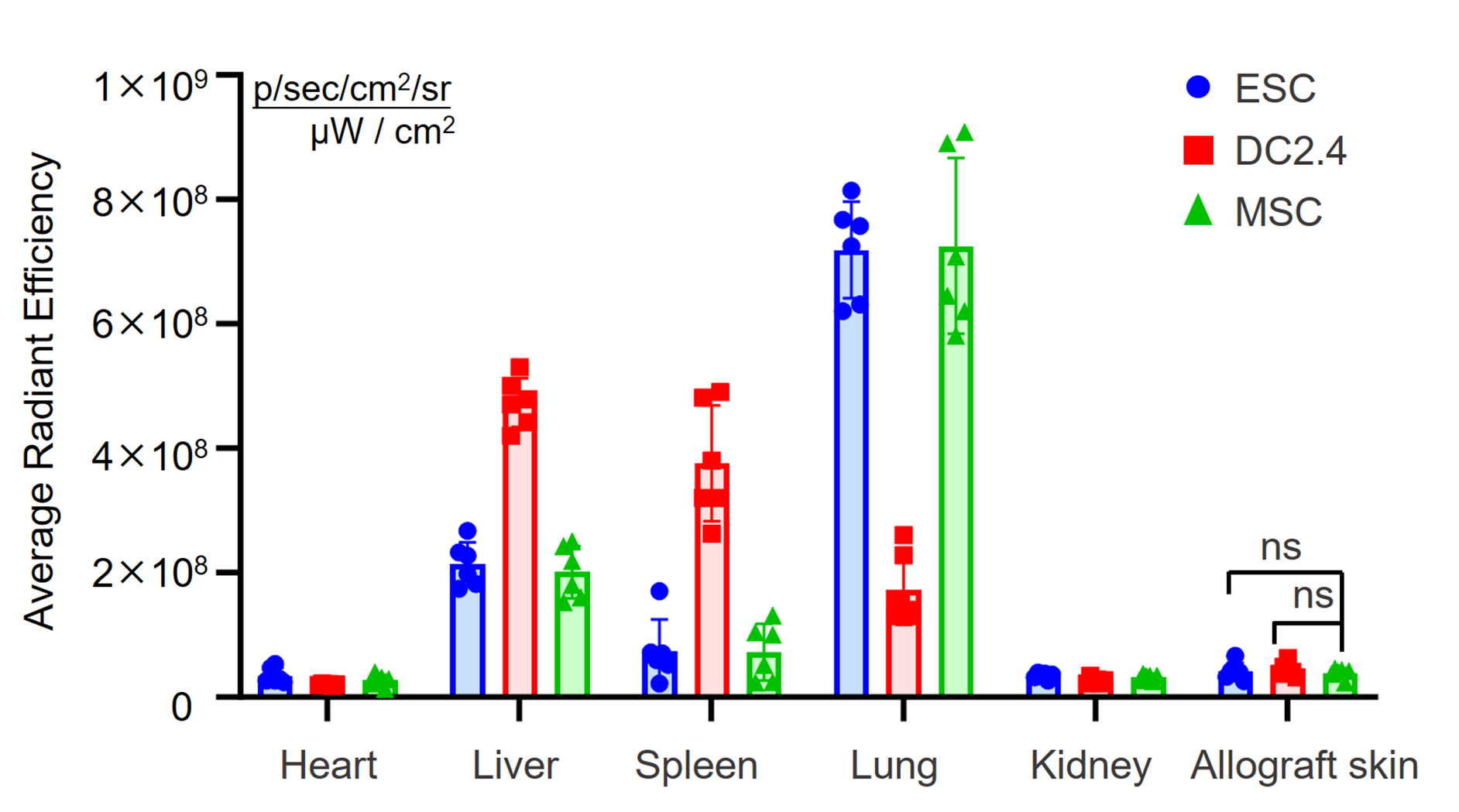


**Figure S25** Quantitative analysis of organ biodistribution. Fluorescence intensity quantification of major organs (liver, spleen, lung, kidney) and allograft skin tissue from the ESC, DC2.4, and MSC groups. Data are presented as mean ± SD (n=6). "ns" indicates no statistical significance between control groups in graft accumulation. Data are presented as mean ± SEM. Normally distributed quantitative data were analyzed using Student's t-test (for two-group comparisons) or one-way ANOVA with Dunnett's *post-hoc* test.


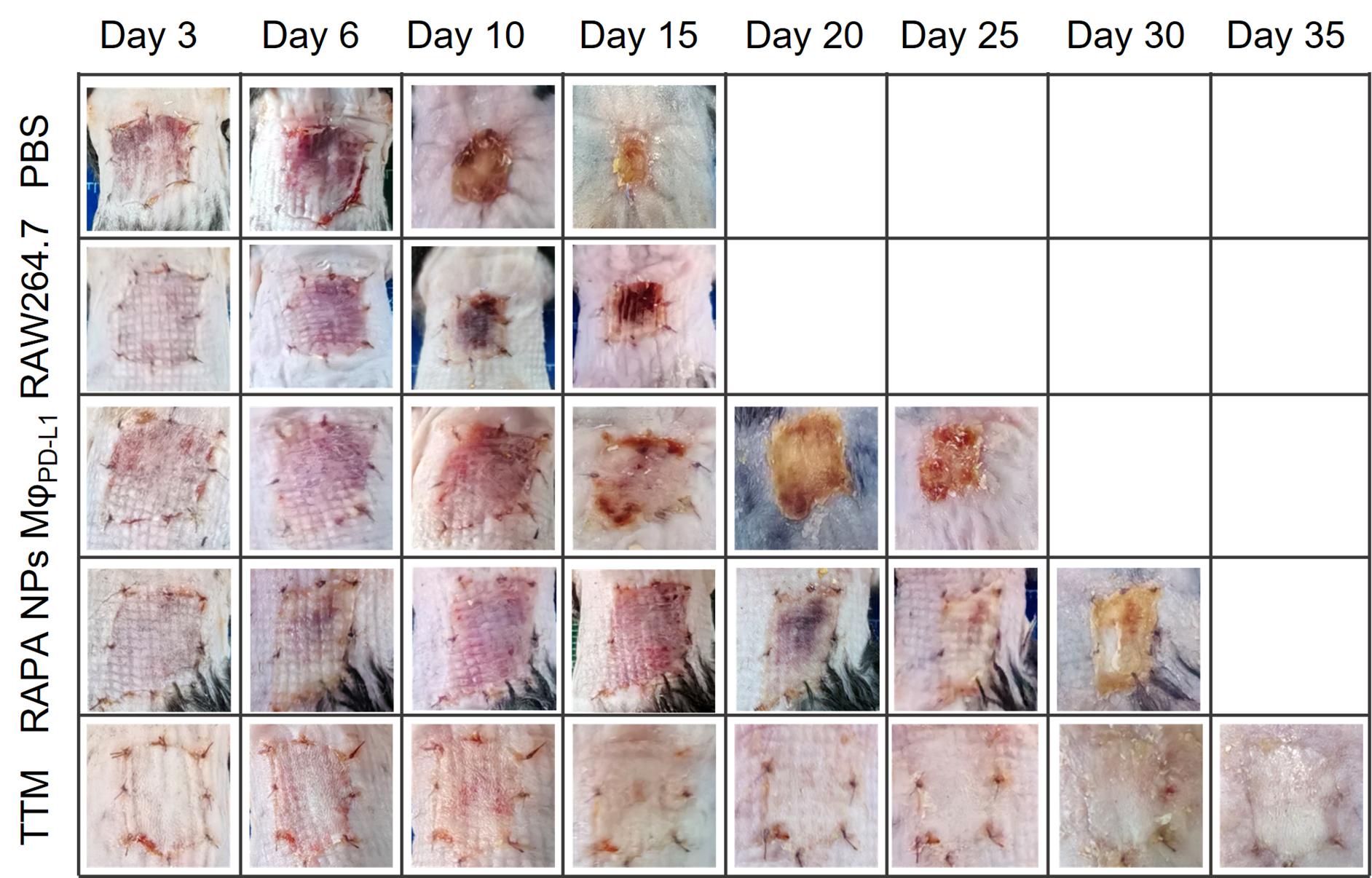


Figure S26 Images of the allografts subjected to different treatment modalities on days 3, 6, 10, 15, 20, 25, 30 and 35 post-skin transplantation.


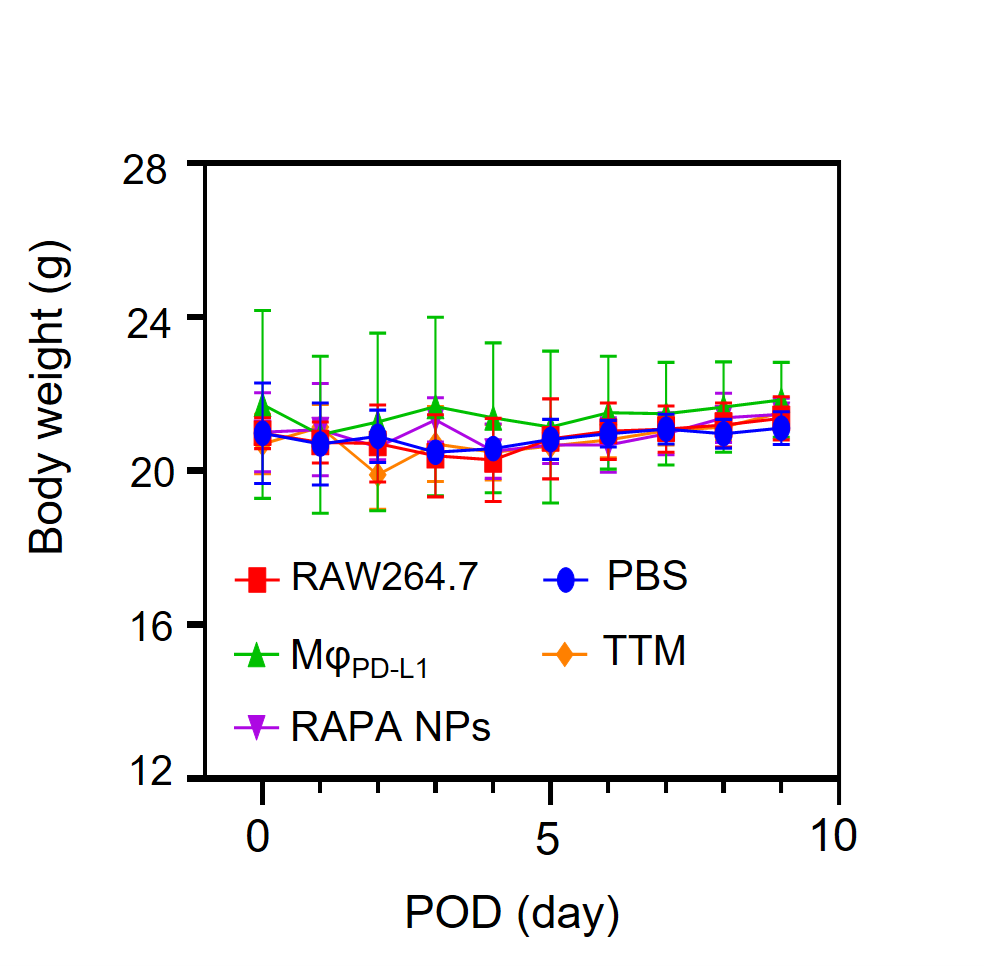


**Figure S27** The monitoring of body weight changes in allograft mice from postoperative day (POD) 0 to POD 9. n=5.


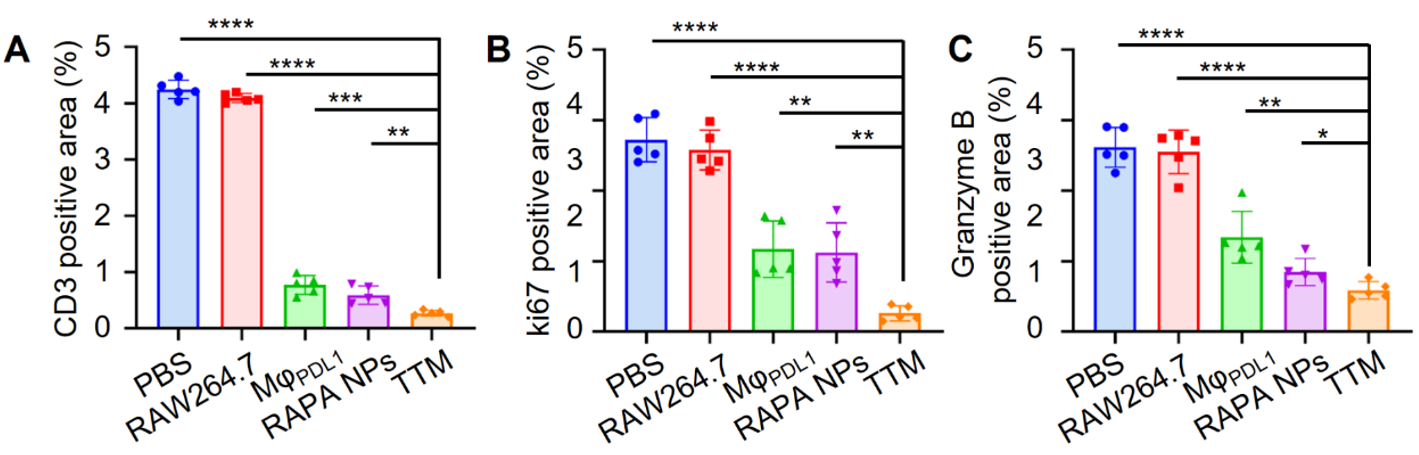


**Figure S28** On the 9th day post-transplantation, immunohistochemical analysis was performed on the allografts, and quantitative analysis of the positive areas for CD3 (A), Ki67 (B), and Granzyme B (C) in the images was conducted. n = 5. Corresponding images are presented in **Figure 6D-F**. All data are expressed as mean ± SEM. n=5, statistical significance was defined as **P* < 0.05, ***P* < 0.01, ****P* < 0.001, *****P* < 0.0001. For normally distributed datasets, statistical analyses included Student's t-test (for two-group comparisons) or one-way ANOVA with Dunnett’s *post-hoc* test.


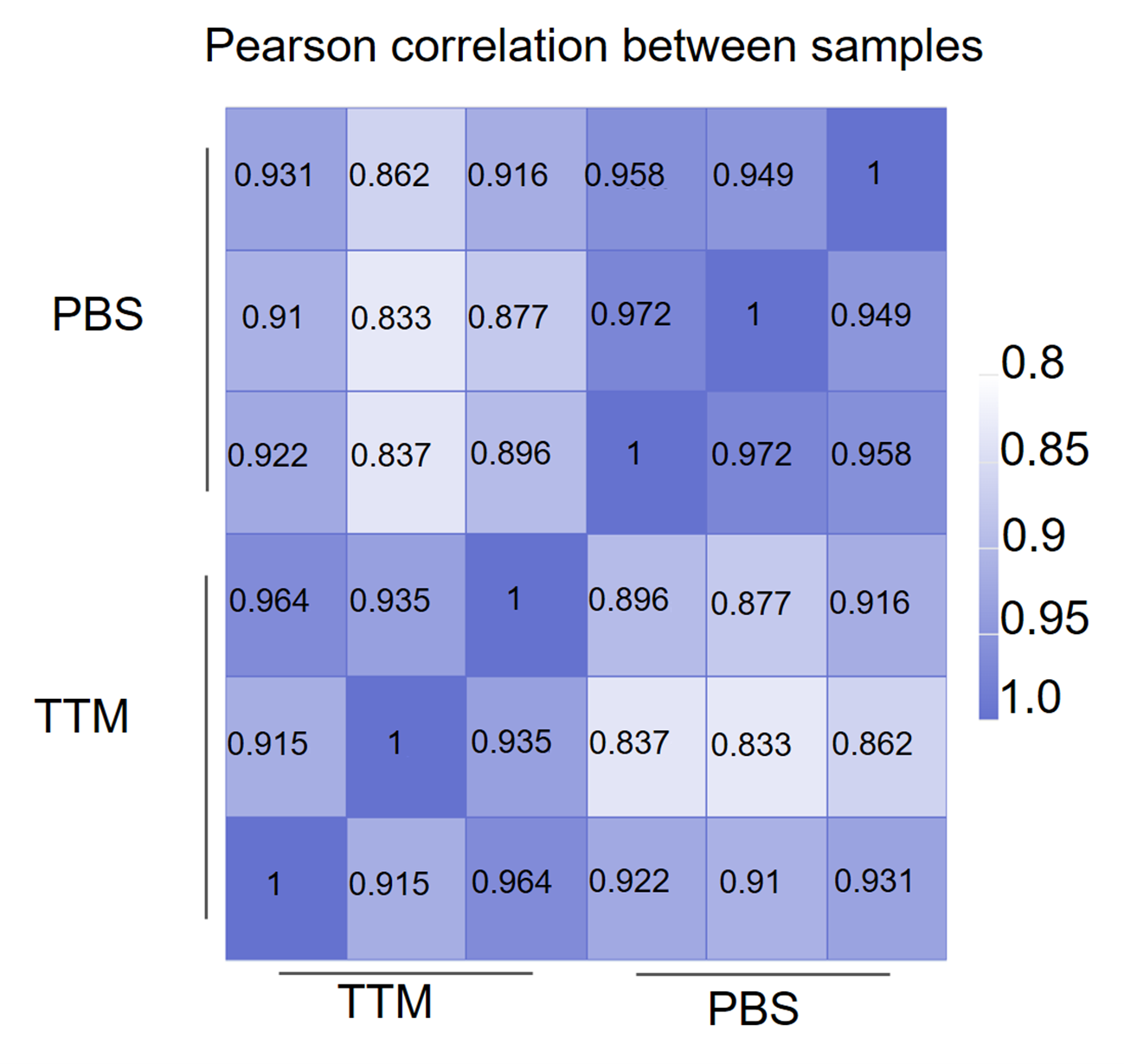


**Figure S29** RNA sequencing analysis was conducted on the grafts at day 9 post-allogeneic transplantation to assess the correlation between PBS and TTM group grafts. n=3.


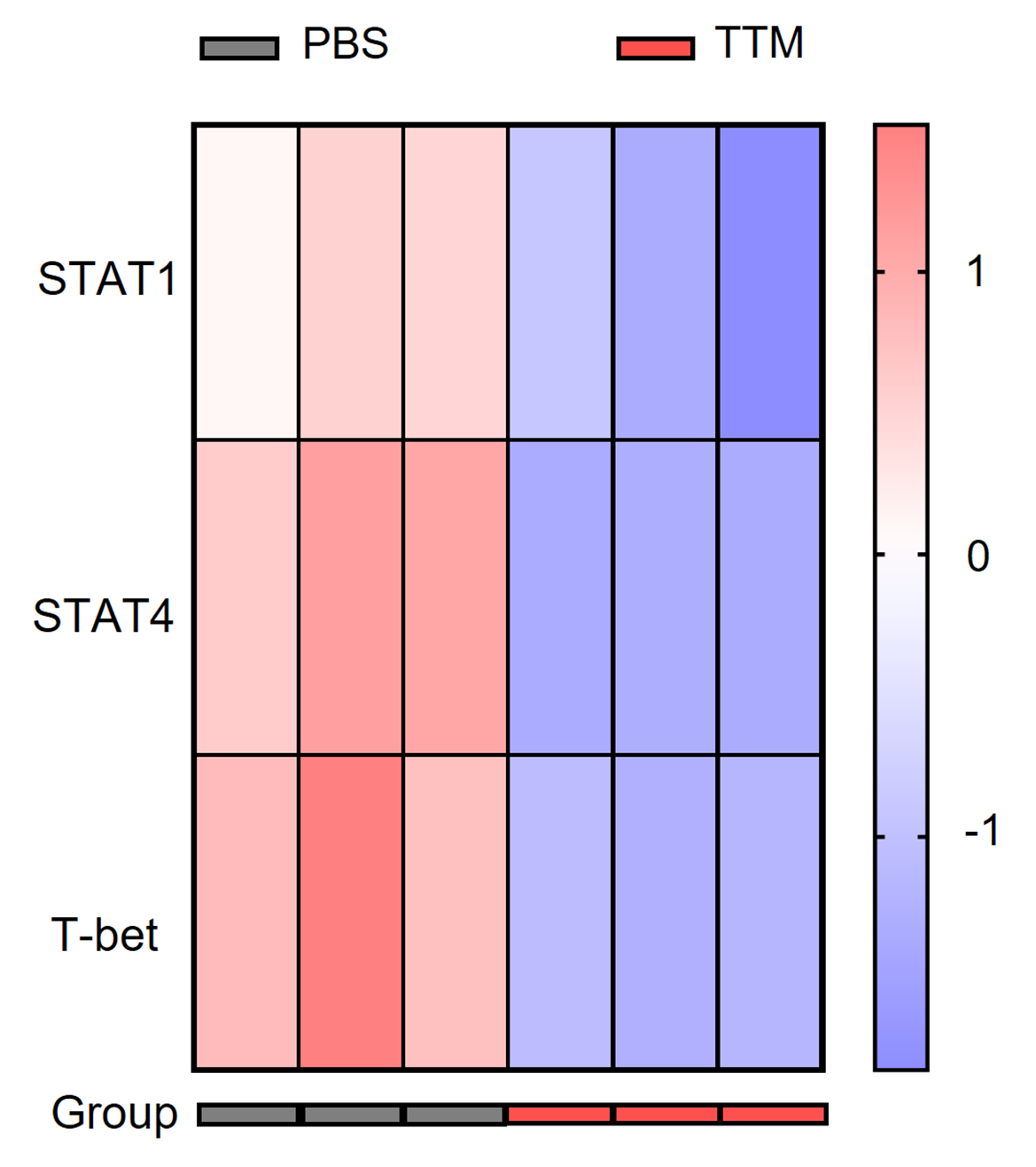


**Figure S30** Heatmap analysis of STAT1, STAT4, and T-bet gene expression levels in graft skin tissues. n=3.


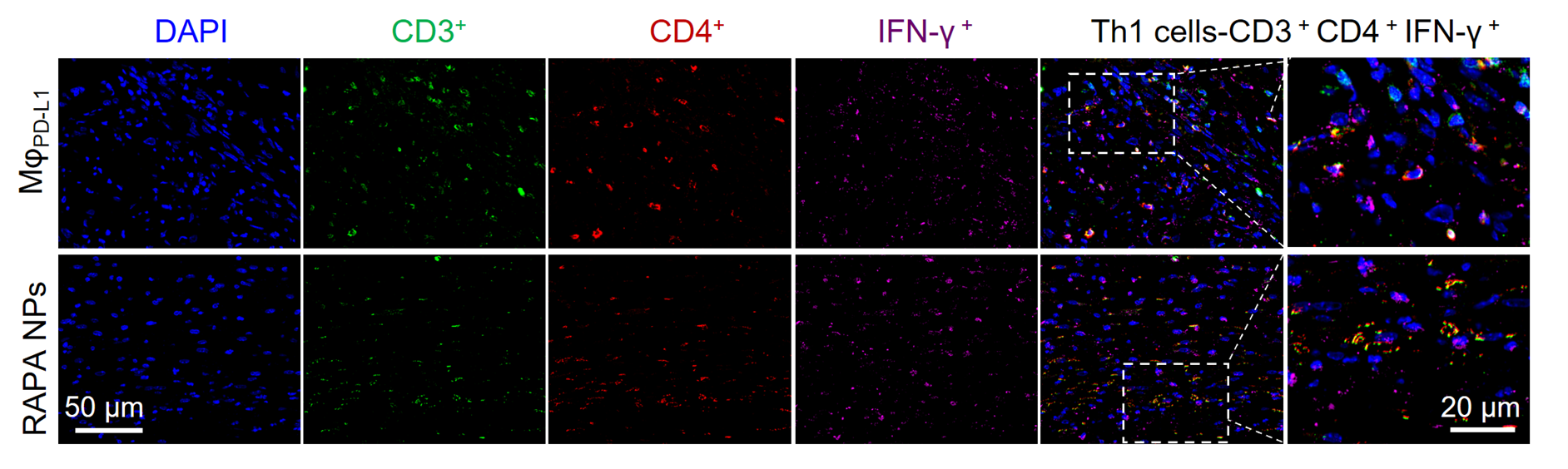


**Figure S31** Immunofluorescence images reveal Th1 cell infiltration in the grafts of the MφPD-L1 and RAPA NPs groups, as observed through triple staining with CD3 (green), CD4 (red), and IFN-γ (purple). The combined CD3⁺CD4⁺IFN-γ⁺ cells indicate the Th1 population. Scale bar=50 μm.

**
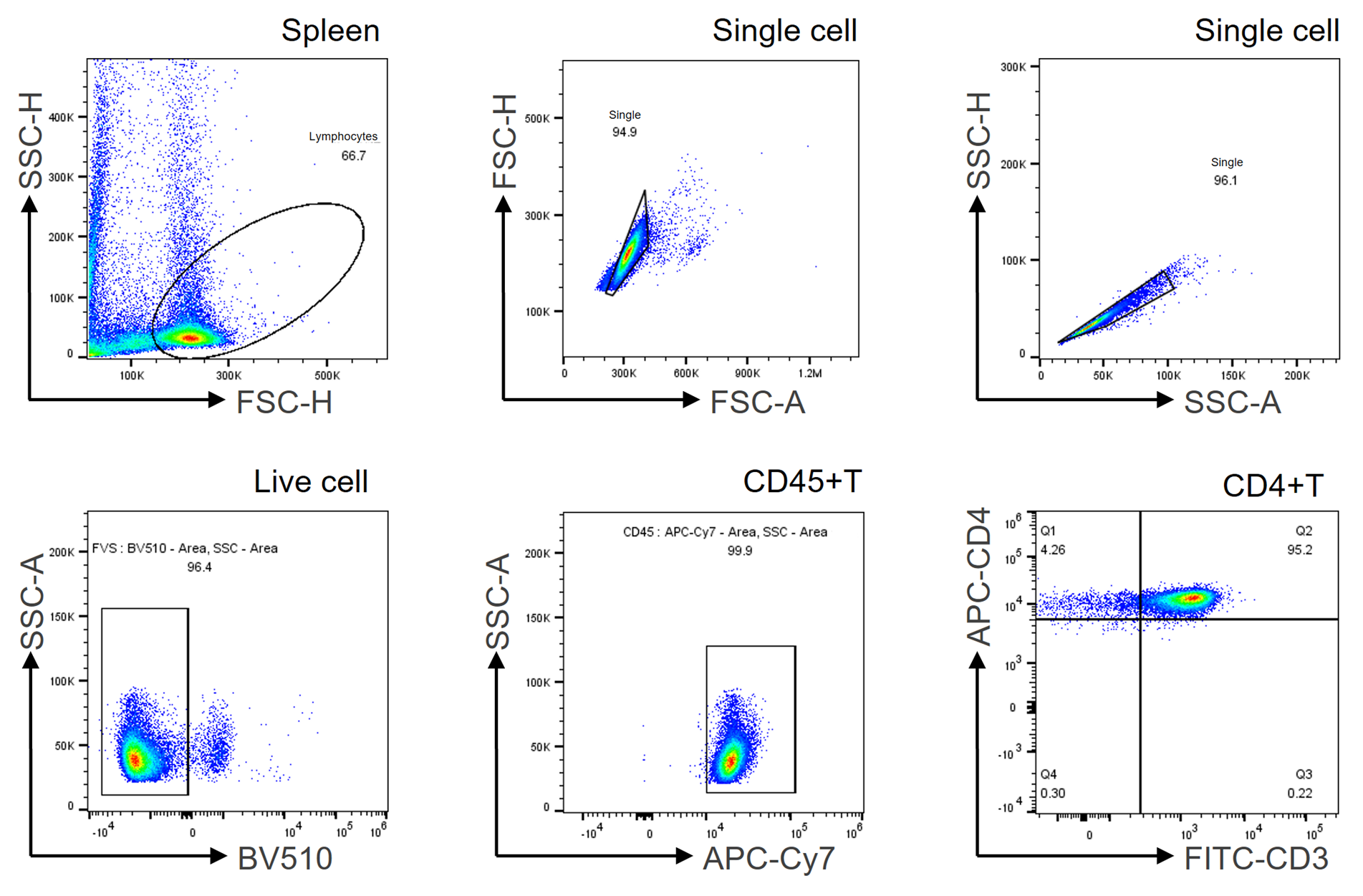
**

**Figure S32** Gating strategy for flow cytometry to select CD4^+^ T cells from C57BL/J6 mouse spleen, n=5. The CD4^+^ T cells used in Figure 7 were established based on this gating strategy.

**
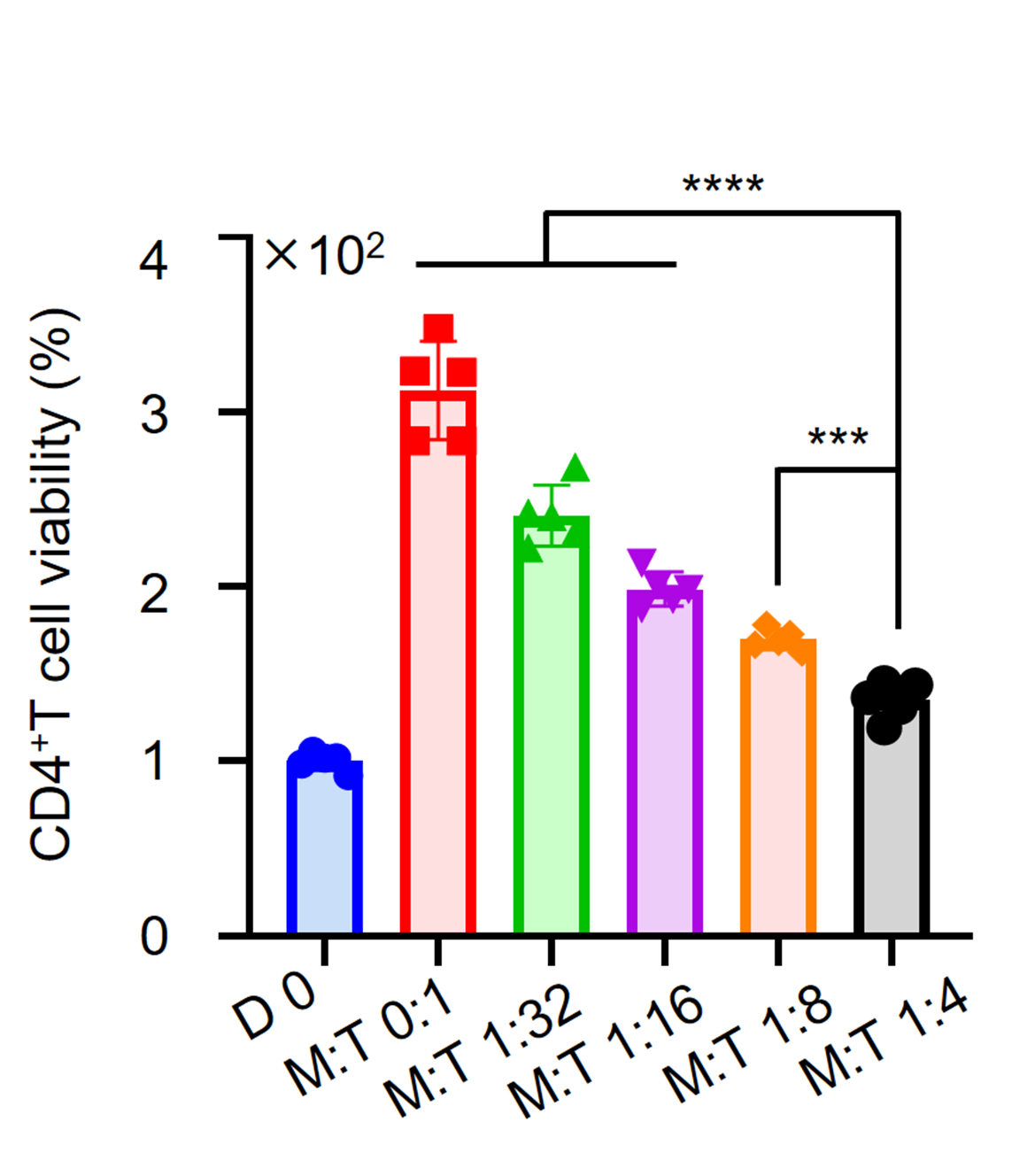
**

**Figure S33 TTM** cells were co-cultured with CD4^+^ T cells at ratios of 0:1, 1:4, 1:8, 1:16, and 1:32 (M:T). Cell viability of CD4^+^ T cells was assessed using the CCK8 assay after incubation. D0 represents CD4^+^ T cells without CD3 and CD28 antibody activation, while all other CD4^+^ T cells underwent antibody induction. All data are expressed as mean ± SEM. n=5, statistical significance was defined as ****P* < 0.001, *****P* < 0.0001. For normally distributed datasets, statistical analyses included Student's t-test (for two-group comparisons) or one-way ANOVA with Dunnett’s *post-hoc* test.


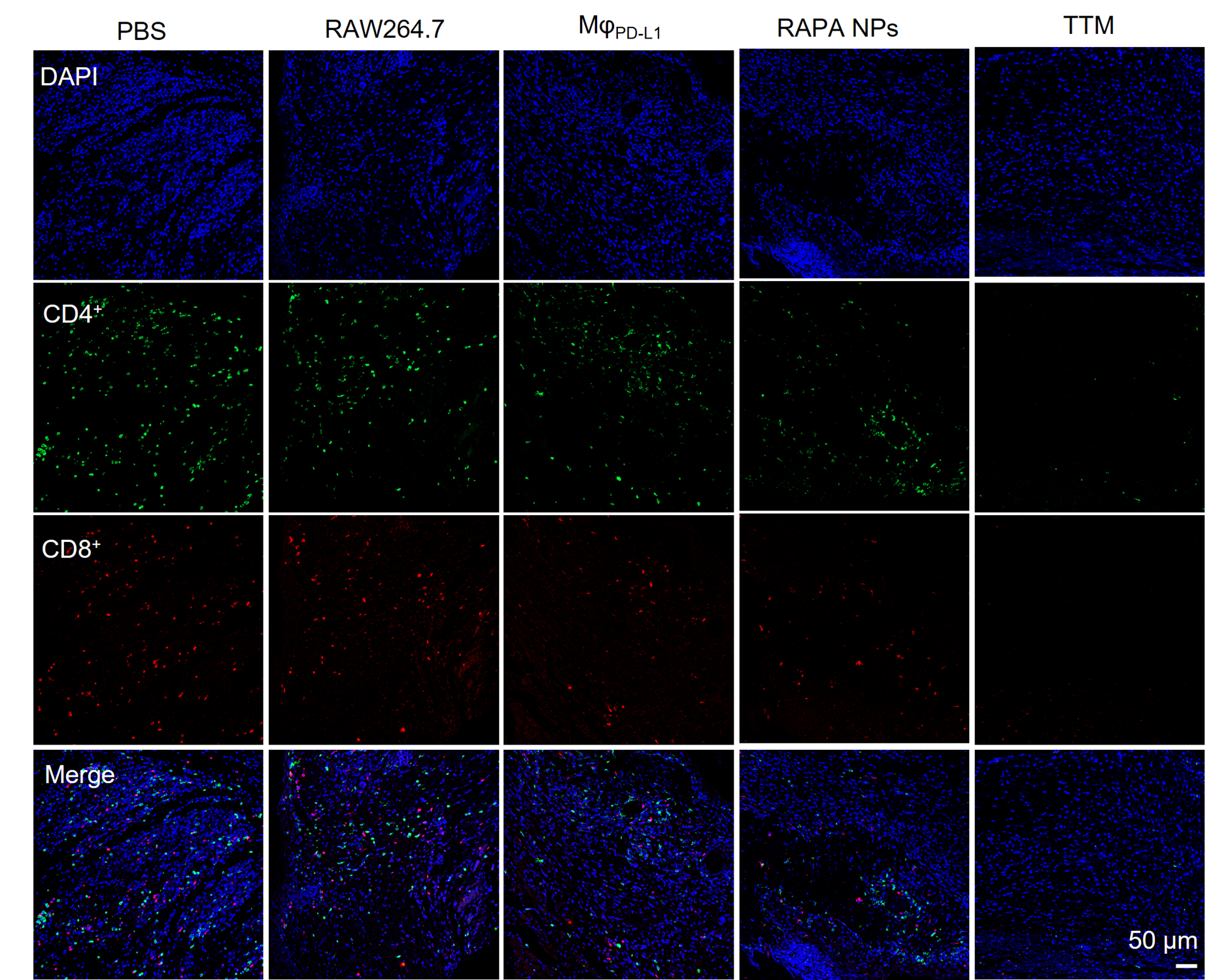


**Figure S34** On day 9 post-allogeneic transplantation, immunofluorescence was used to detect the content of CD8^+^ T cells in the grafts. Scale bar: 50 μm. n=5.


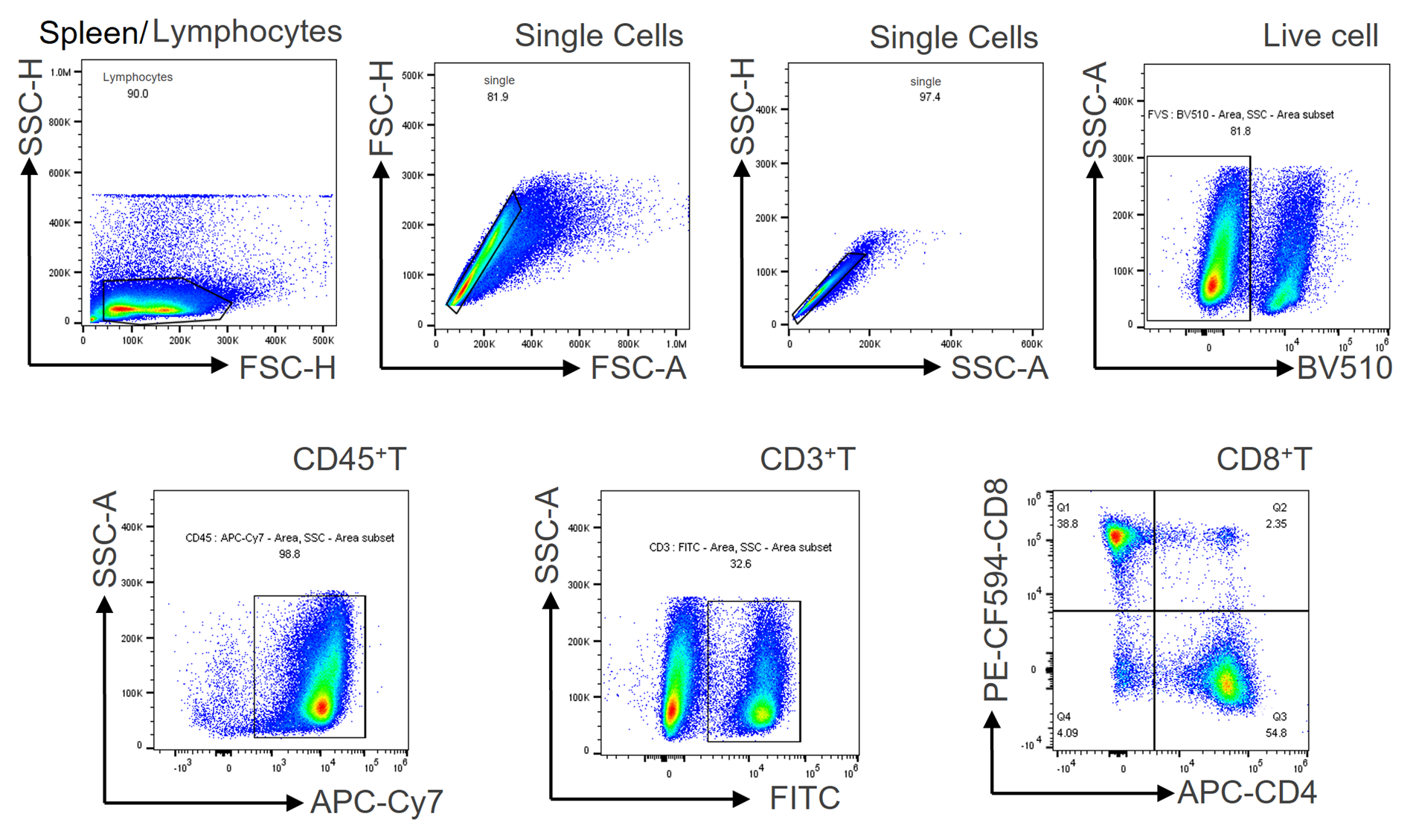


Figure S35 Gating strategy for spleen-derived CD8^+^T cells in Figure 8A, with gating performed under the same voltage conditions for all groups (PBS, RAW264.7, Mφ_PD-L1_, RAPA NPs, and TTM).

**
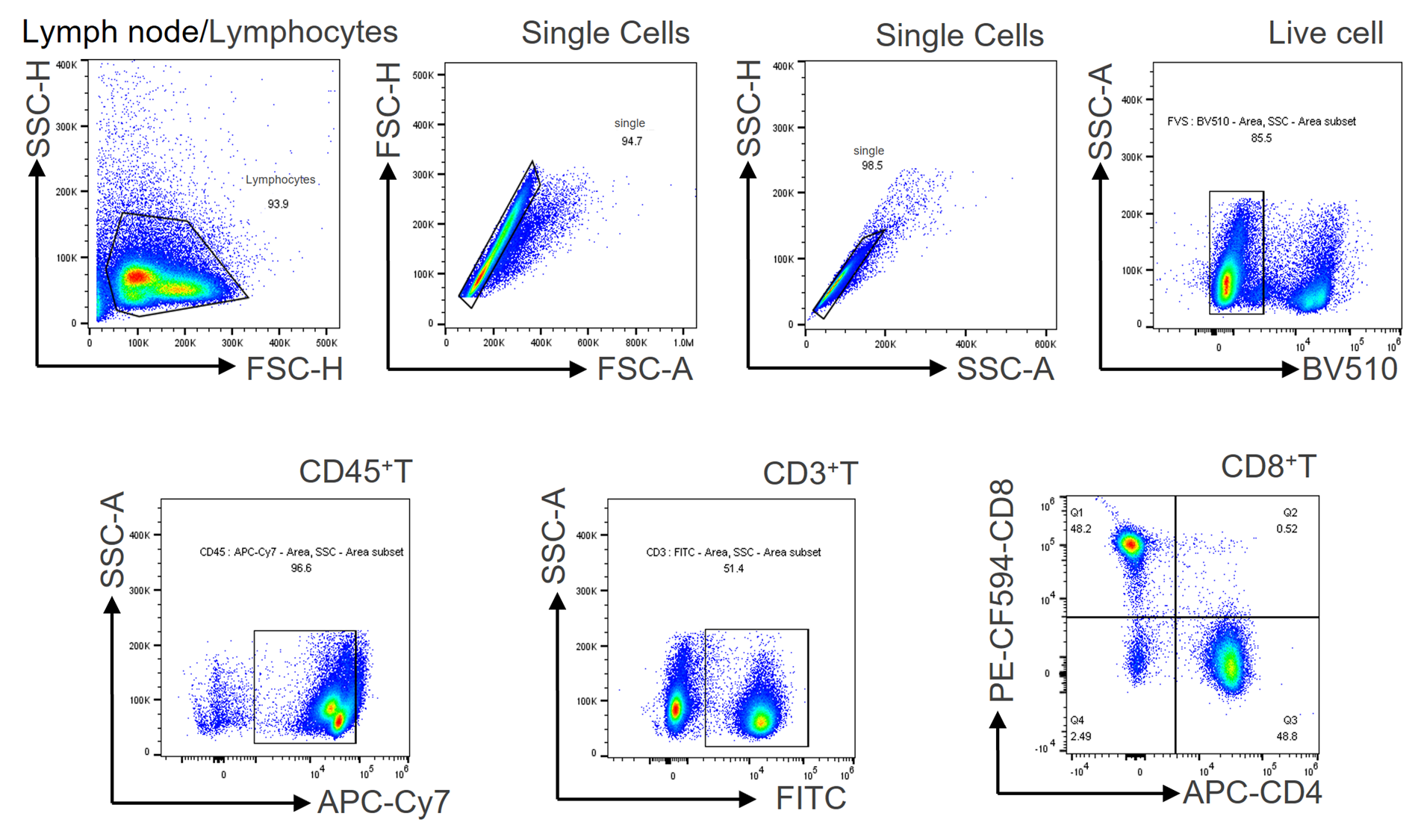
**

**Figure S36** Gating strategy for lymph node-derived CD8^+^T cells in **Figure 8C**, with gating performed under the same voltage conditions for all groups (PBS, RAW264.7, Mφ_PD-L1_, RAPA NPs, and TTM).


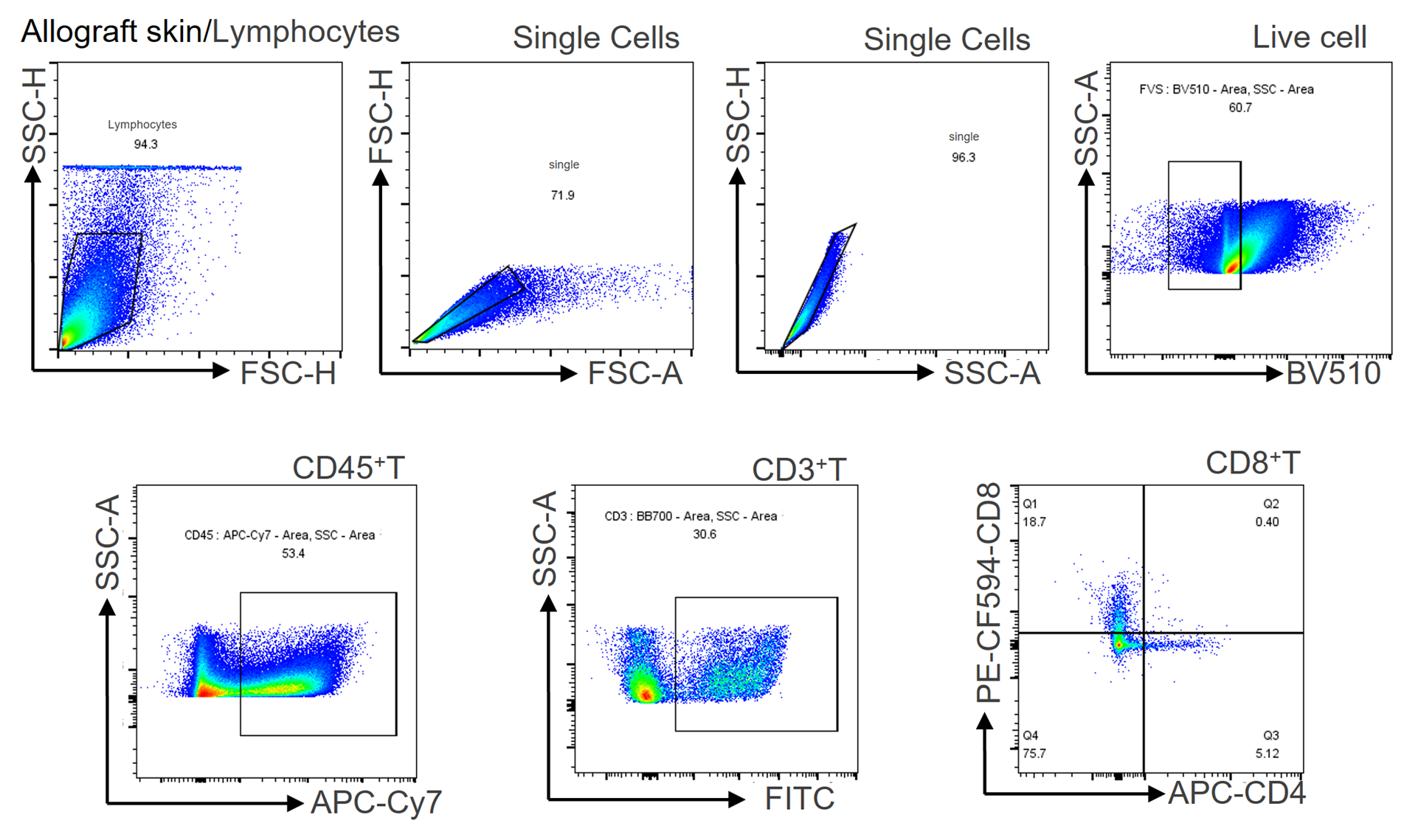


**Figure S37** Gating strategy for allograft skin-derived CD8^+^T cells in **Figure 8E**, with gating performed under the same voltage conditions for all groups (PBS, RAW264.7, Mφ_PD-L1_, RAPA NPs, and TTM).

**
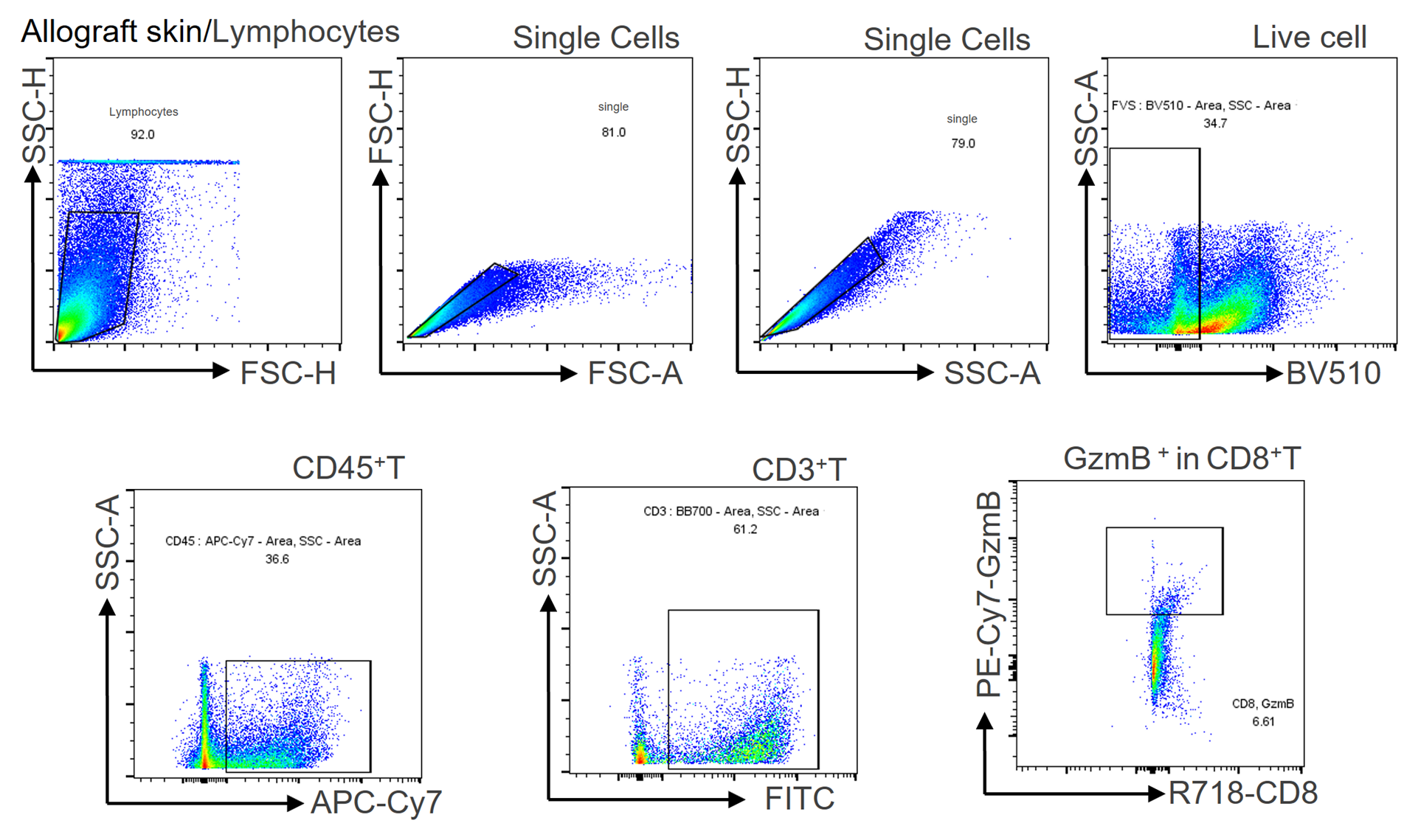
**

**Figure S38** Gating strategy for allograft skin-derived CD8⁺IFN-γ⁺T cells in **Figure 8G**, with gating performed under the same voltage conditions for all groups (PBS, RAW264.7, Mφ_PD-L1_, RAPA NPs, and TTM).

**
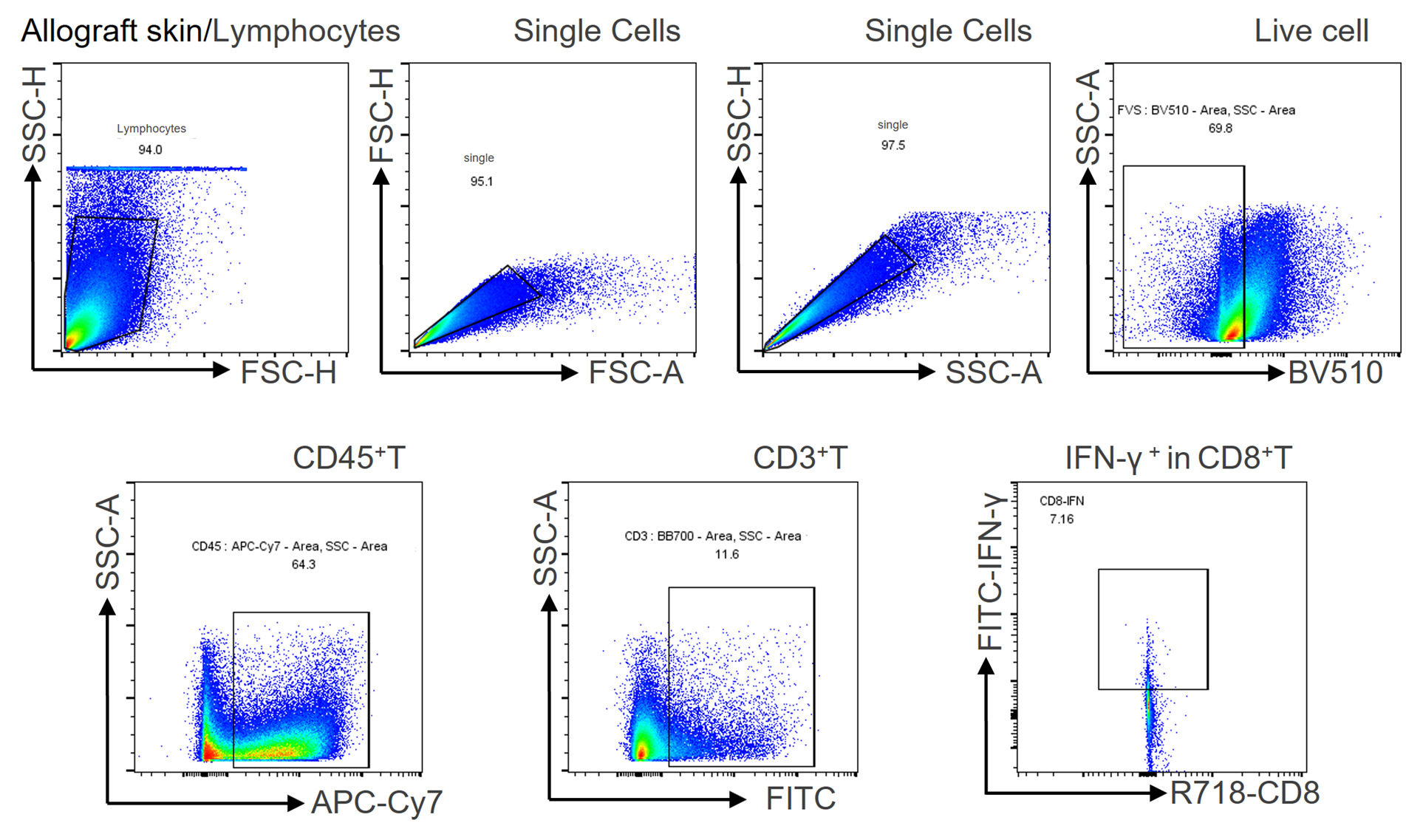
**

**Figure S39** Gating strategy for allograft skin-derived CD8⁺GzmB⁺T cells in **Figure 8I**, with gating performed under the same voltage conditions for all groups (PBS, RAW264.7, Mφ_PD-L1_, RAPA NPs, and TTM).


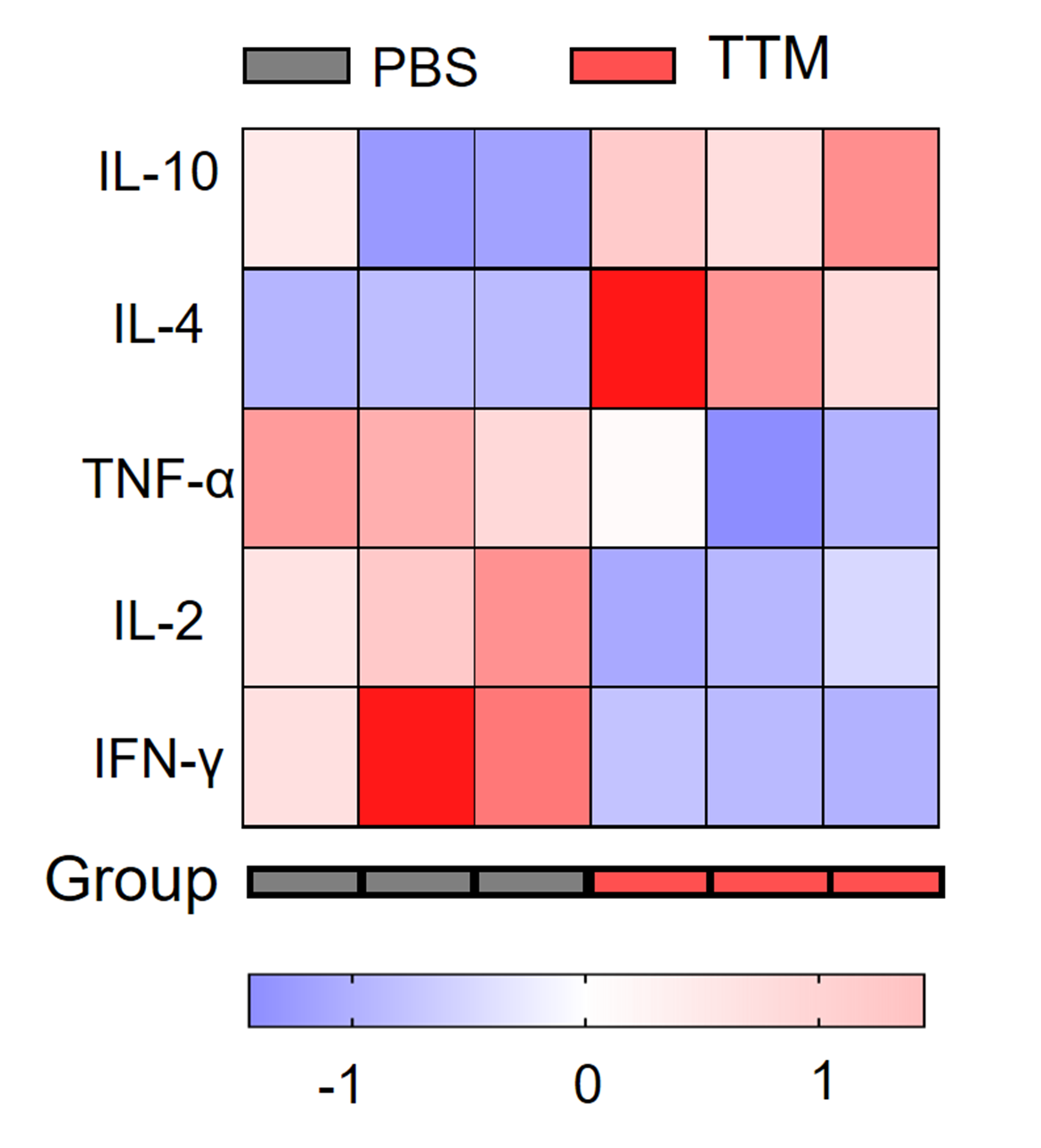


**Figure S40** Heatmap analysis was used to describe the gene expression levels of IL-10, IL-4, IL-2, IFN-γ, and TNF-α in the allografts on day 9 post-transplantation. n=3.


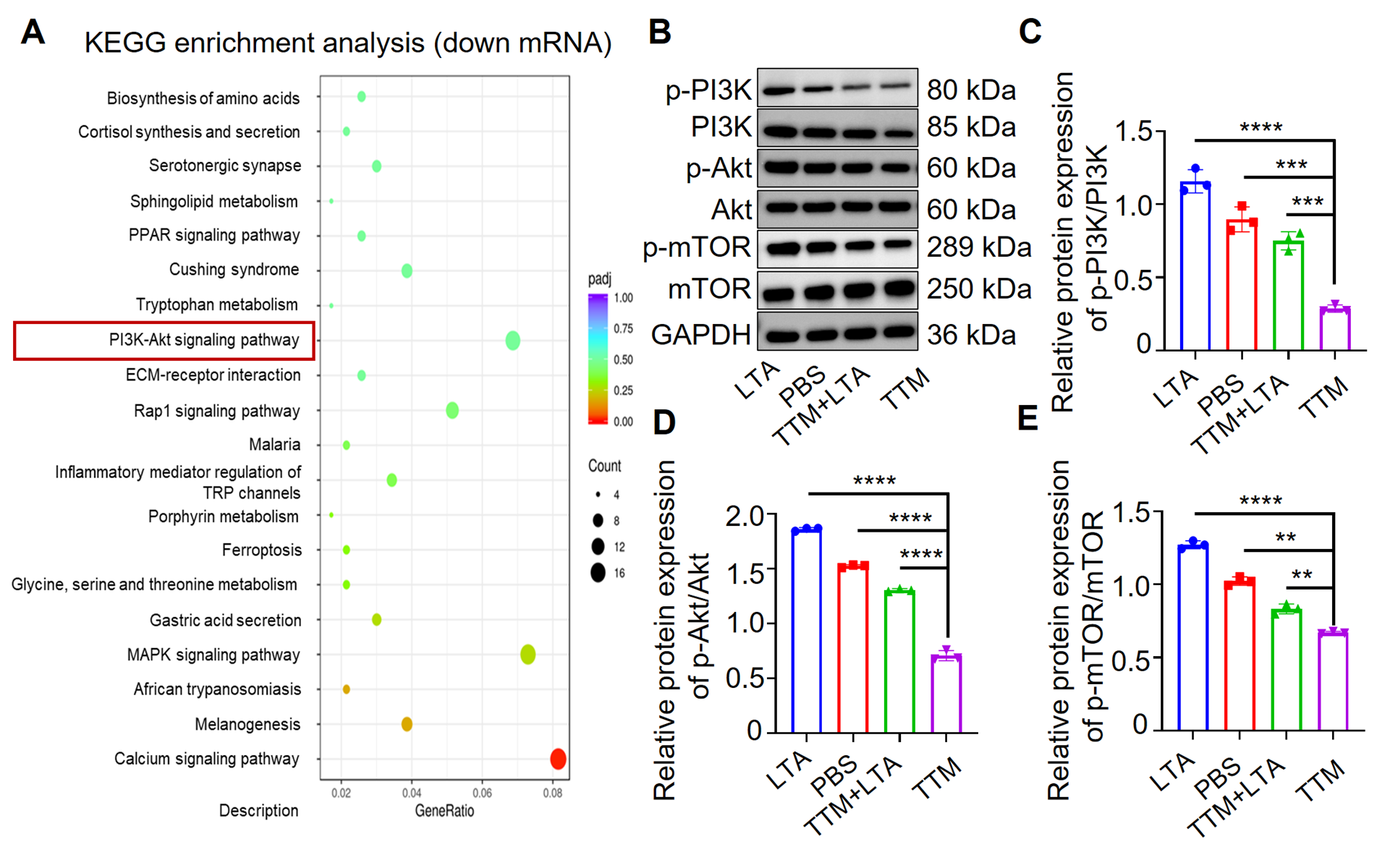


**Figure S41** TTM inhibits complement activation, which subsequently interferes with the PI3K/Akt pathway to regulate mTOR activity. (A) KEGG enrichment analysis of top 20 significantly downregulated genes in the allografts of the TTM group compared to the RAPA NPs group. padj<0.05. Red box indicates significantly enriched pathways. (B-E) Subsequently, groups were established as follows: Lipoteichoic acid (LTA), PBS, TTM, and TTM + LTA. LTA was used as a complement activator and administered to mice via oral gavage (8ug/μL,100 μL,7 days) to further investigate the therapeutic effects of TTM through a rescue experiment. (B) Western blot analysis of protein expression levels of p-PI3K, PI3K, p-Akt, Akt, p-mTOR, and mTOR in allograft skin on the 9th day after transplantation. (D-E) Corresponding quantitative analysis of the protein expression levels for the corresponding bands. n=3, with statistical significance denoted as ***P* < 0.01, ****P* < 0.001, *****P* < 0.0001. All quantitative data are expressed as mean ± SEM. For normally distributed datasets, statistical analyses included Student's t-test (for two-group comparisons) or one-way ANOVA with Dunnett’s *post-hoc* test.

**
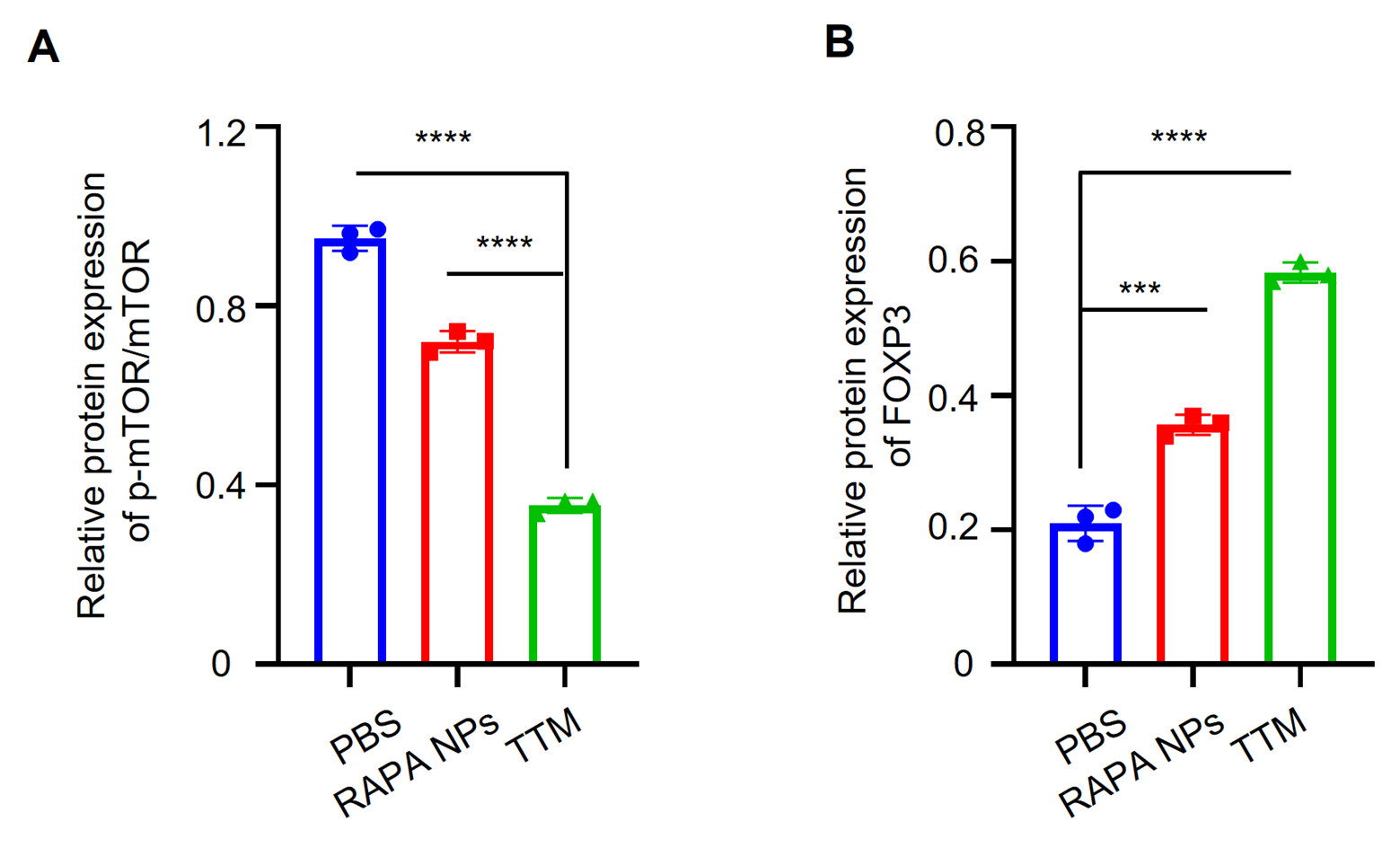
**

**Figure S42** Quantitative analysis of the p-mTOR, mTOR, and Foxp3 protein bands in **Figure 9B**. ****P* < 0.001, *****P* < 0.0001. All data are expressed as mean ± SEM. n=3, statistical significance was defined as **P* < 0.05, ***P* < 0.01, ****P* < 0.001, *****P* < 0.0001. Statistical analysis for normally distributed datasets utilized Student's t-test (for two-group comparisons) or one-way ANOVA with Dunnett’s *post-hoc* test.


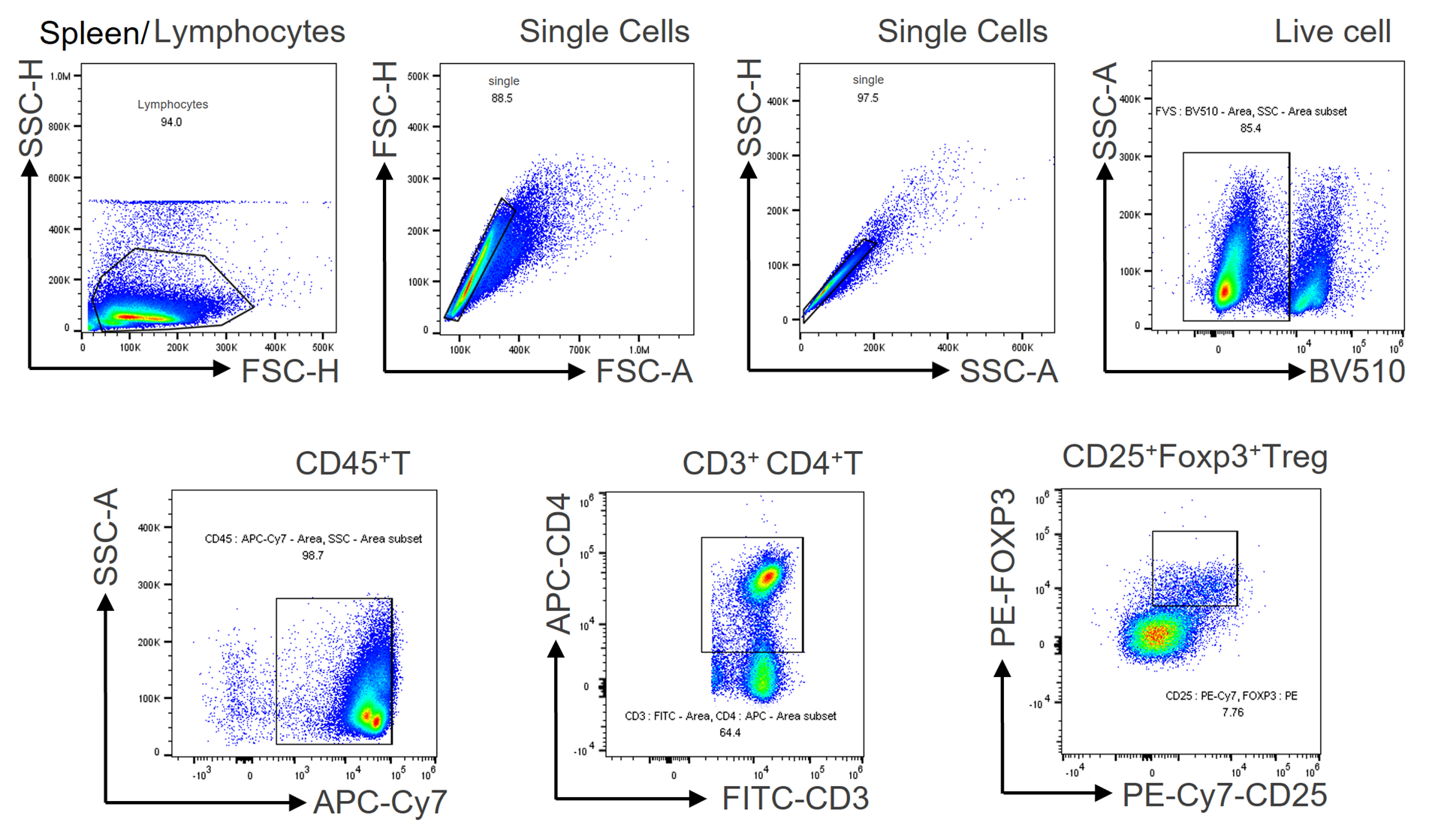


**Figure S43** Gating strategy for spleen-derived CD25^+^Foxp3^+^Treg cells in **Figure 9E**, with gating performed under the same voltage conditions for all groups (PBS, RAW264.7, Mφ_PD-L1_, RAPA NPs, and TTM).


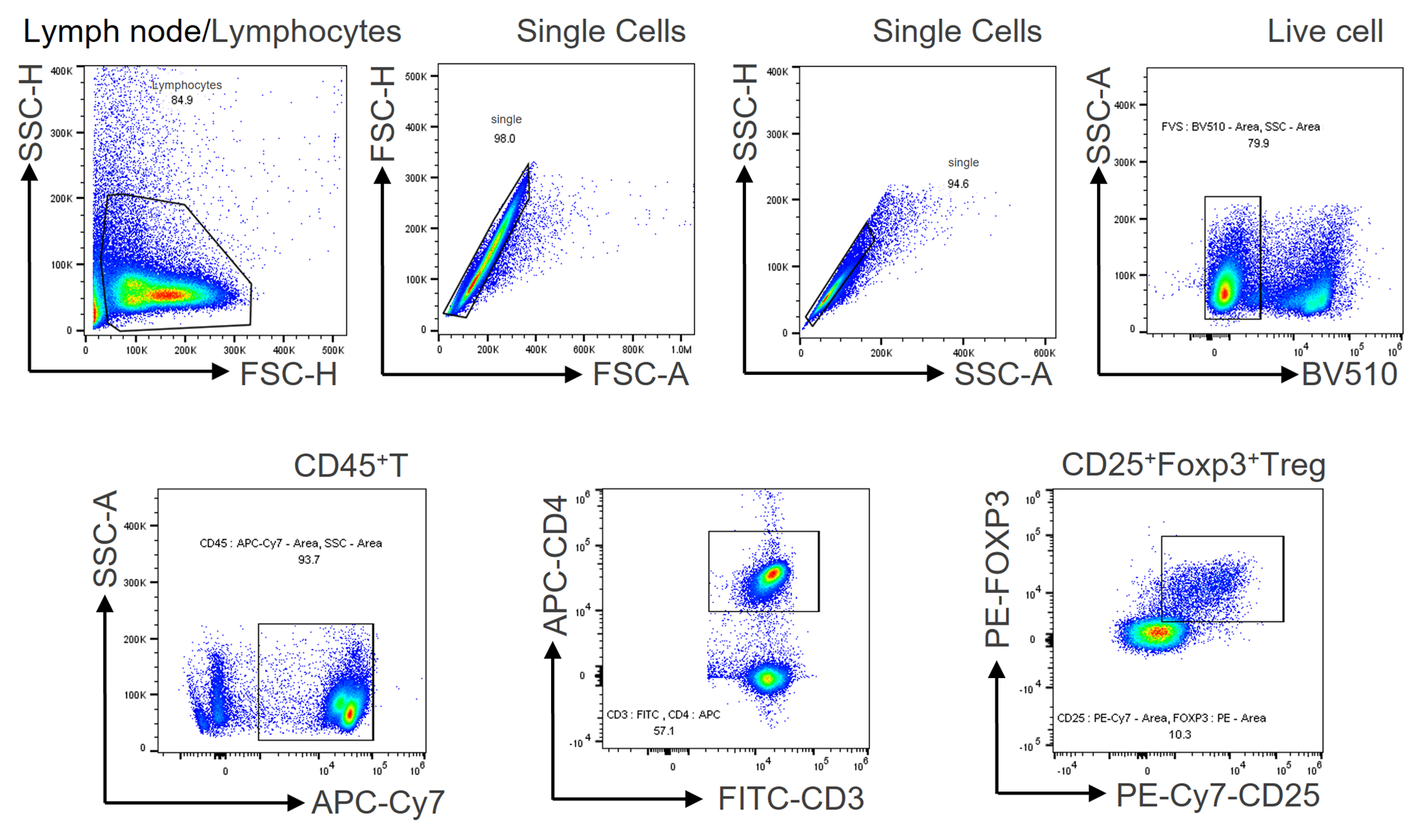


Figure S44 Gating strategy for lymph node-derived CD25^+^Foxp3^+^Treg cells in Figure 9G, with gating performed under the same voltage conditions for all groups (PBS, RAW264.7, Mφ_PD-L1_, RAPA NPs, and TTM).

**
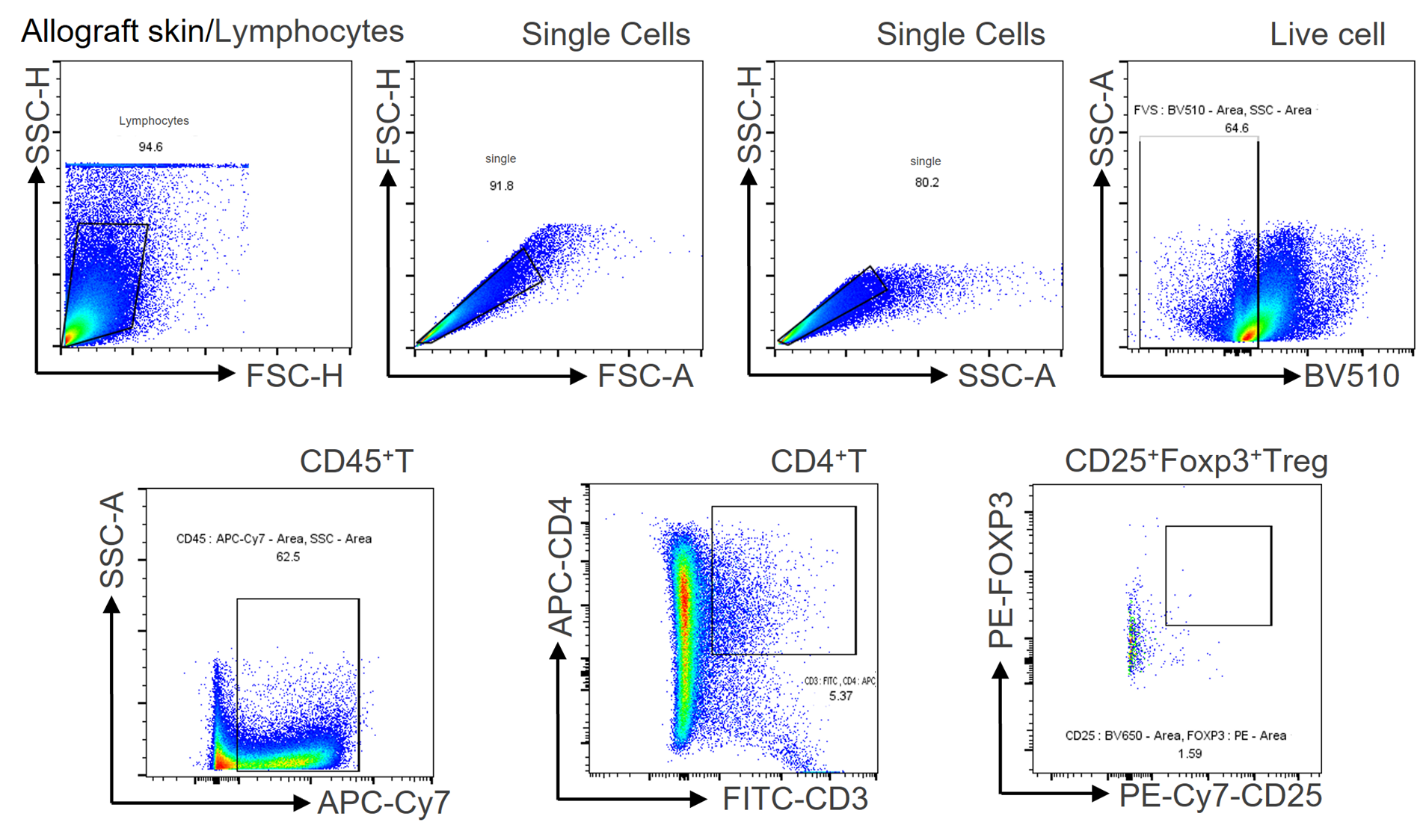
**

**Figure S45** Gating strategy for allograft skin-derived CD25^+^Foxp3^+^Treg cells in **Figure 9I**, with gating performed under the same voltage conditions for all groups (PBS, RAW264.7, Mφ_PD-L1_, RAPA NPs, and TTM).


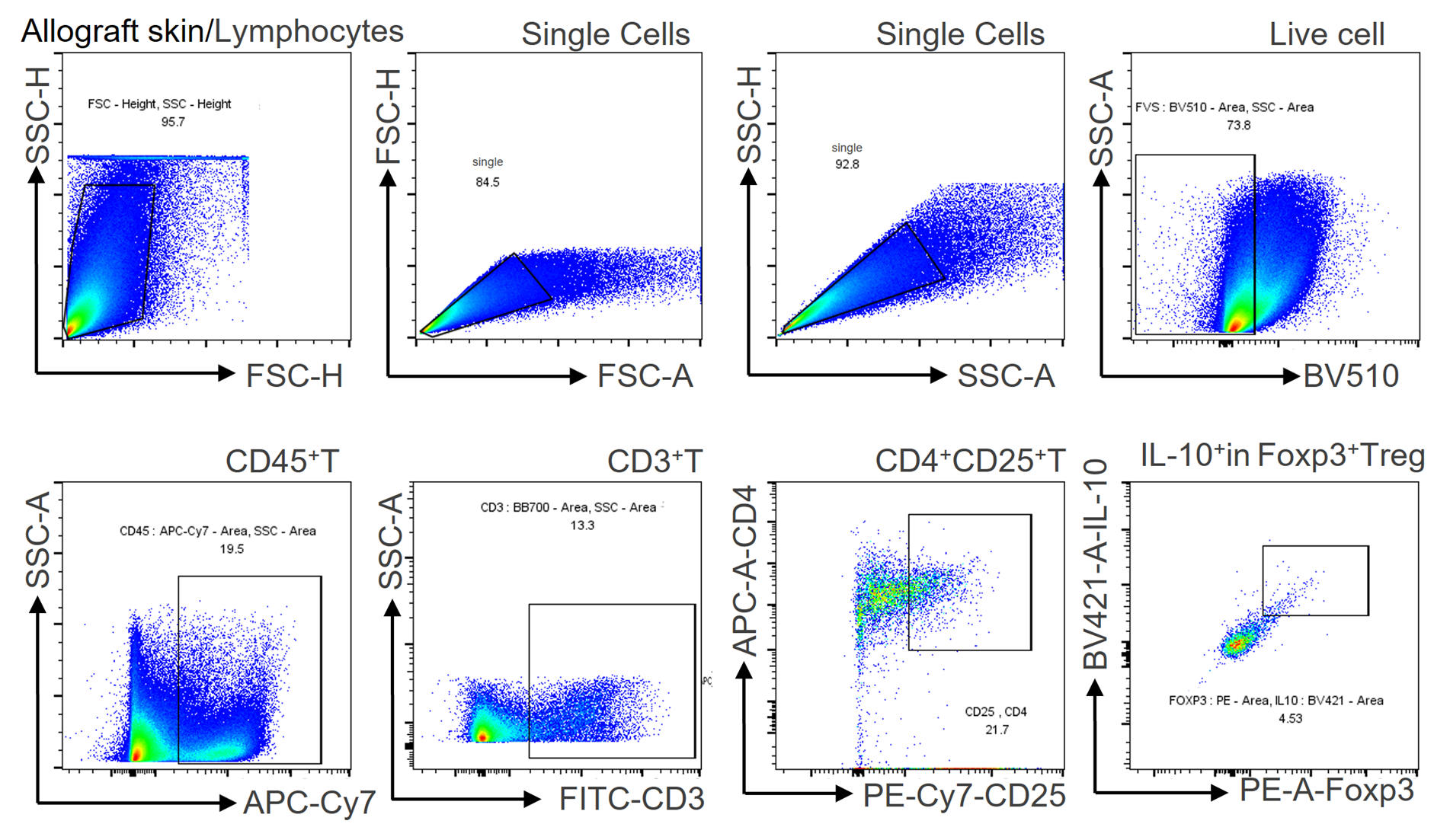


**Figure S46** Gating strategy for allograft skin-derived IL-10^+^ Treg cells in **Figure 9K**, with gating performed under the same voltage conditions for all groups (PBS, RAW264.7, Mφ_PD-L1_, RAPA NPs, and TTM).

**Supporting Tables**

**Table S1.** Detailed information on antibody reagents.

| **Antibodies** | **Source** | | **Identifier** |
| --- | --- | --- | --- |
| Anti-PD-L1 | | Abcam | Cat# ab213480 |
| Anti-CSF-1-R | | Abcam | Cat# ab254357 |
| Anti-CD68 | | Abcam | Cat# ab125212 |
| Anti-MCP1 | | Abcam | Cat# ab214819 |
| Anti-CD3 | | Abcam | Cat# ab135372 |
| Anti-CD4 | | Abcam | Cat# ab133616 |
| Anti-CD8 | | Abcam | Cat# ab217344 |
| Anti-Ki67 | | Abcam | Cat# ab15580 |
| Anti-Granzyme B | | Abcam | Cat# ab255598 |
| Anti-FOXP3 | | Abcam | Cat# ab215206 |
| CD206/MRC1 (E6T5J) XP^®^ Rabbit mAb | | Cell Signaling Technology | Cat# 24595 |
| Anti-PI 3 Kinase p85 alpha | | Abcam | Cat# ab191606 |
| Anti-AKT2 | | Abcam | Cat# ab179463 |
| Recombinant Anti-AKT2 (phospho S473) antibody | | Abcam | Cat# ab192623 |
| Anti-mTOR | | Abcam | Cat# ab134903 |
| FITC-labeled Goat Anti-Rabbit IgG (H+L) | | Beyotime Biotechnology | Cat# A0562 |
| Ultra-LEAF purified anti-mouse CD3 | | Biolegend | Cat# 100256 |
| Ultra-LEAF purified anti-mouse CD28 | | Biolegend | Cat# 102132 |
| PE-Cy7 rat anti-mouse CD86 | | BD Biosciences | Cat# 560582 |
| APC-Cy7 rat anti-mouse CD45 | | BD Biosciences | Cat# AM08044AC7-N |
| Rat Anti-Mouse CD16/CD32 (Mouse BD Fc Block™) | | BD Biosciences | Cat# 553141 |
| BD Horizon™ BV421 Rat Anti-Mouse IL-10 | | BD Biosciences | Cat# 563276 |
| BD Pharmingen™ FITC Rat Anti-Mouse IFN-γ | | BD Biosciences | Cat# 554411 |
| Granzyme B Monoclonal Antibody (NGZB), PE-Cyanine7 | | Thermo Fisher Scientific | Cat# 25-8898-82 |

**Table S2.** Primer sequence of MCP-1, CSF-1 and CD68.

| Primers | Sequence (5’ to 3’) |
| --- | --- |
| Mouse actin F | CATTGCTGACAGGATGCAGAAGG |
| Mouse actin R | TGCTGGAAGGTGGACAGTGAGG |
| Mouse MCP-1 F | CTAGACCACACTCCCGTCCT |
| Mouse MCP-1 R | ACAGTGTGCAGCAGTCAACA |
| Mouse CSF-1 F | TGGATGCCTGTGAATGGCTCTG |
| Mouse CSF-1 R | GTGGGTGTCATTCCAAACCTGC |
| Mouse CD68 F | GGGGCTCTTGGGAACTACAC |
| Mouse CD68 R | GTACCGTCACAACCTCCCTG |

**Table S3.** Primer sequence of C3a, C5a, C3ar, C5ar, CCL5, CCR5.

| Primers | Sequence (5’ to 3’) |
| --- | --- |
| Mouse GAPDH F | TGCCCCCATGTTTGTGATG |
| Mouse GAPDH R | TGTGGTCATGAGCCCTTCC |
| Mouse C3a F | TCGGAAGTGTTGTGAGGAT |
| Mouse C3a R | GTTGCAGCAGTCTATGAAGG |
| Mouse C5a F | AGAAATGCTGCTATGACGGA |
| Mouse C5a R | TTCTTTTCGGATCTTGTTCG |
| Mouse C3ar F | TAACCAGATGAGCACCACCA |
| Mouse C3ar R | TGTGAATGTTGTGTGCATGG |
| Mouse C5ar F | GATGCCACCGCCTGTATAGT |
| Mouse C5ar R | ACGAAGGATGGAATGGTGAG |
| Mouse CCL5 F | ATGAAGATCTCTGCAGCTGCCCTC |
| Mouse CCL5 R | TGCTGGTGTAGAAATACTCCTTGAC |
| Mouse CCR5 F | GTCTACTTTCTCTTCTGGACTCC |
| Mouse CCR5 R | CCAAGAGTCTCTGTTGCCTGCA |

**Supporting movies**

**Movie S1.** The drug release dynamics of engineered macrophage cells (**TTM**) were monitored utilizing the Leica MICA live cell imaging system.

**Movie S2.** LPS was used to induce an inflammatory stimulus to simulate **TTM** cell activation, followed by monitoring the dynamics of exocytosis and the release of DiI NPs using the Leica MICA live cell imaging system.
